# The selective and sequential chemical recycling of commodity polymers with imino-indole zinc(ii) catalysts

**DOI:** 10.1039/d6gc03650c

**Published:** 2026-08-13

**Authors:** Jack A. Stewart, Matthew J. Cullen, Matthew G. Davidson, Matthew D. Jones

**Affiliations:** a Department of Chemistry, University of Bath Bath BA2 7AY UK mj205@bath.ac.uk

## Abstract

Chemical recycling of plastic waste will play a key role in both decoupling the plastics economy from fossil feedstocks and addressing growing global plastic pollution. Real-world plastic waste streams, however, are complex and heterogeneous, which presents a serious challenge to established chemical recycling methods. Here, we present a series of zinc complexes that show high activity for the chemical recycling of poly(lactic acid) (PLA), bisphenol A polycarbonate (BPA-PC), and poly(ethylene terephthalate) (PET). Crucially, the most active of these catalysts is capable of selectively deconstructing a mixed feed of these polymers in succession with selectivity governed by careful modulation of reaction temperature. This sequential approach allows for the products of each reaction to be isolated in high yields without the need for complex separations. Further, high activity is demonstrated for the direct depolymerization of PLA to lactide. This work demonstrates the feasibility of sequential chemical recycling of mixed plastic waste as a tool for a more circular plastics economy.

## Abstract 2

We report four zinc imino-indole complexes that are highly active for the chemical recycling of key plastics. The catalyst is capable of sequentially degrading a three-component mixture of commodity plastics, showing >99% selectivity in each step.

## Graphical abstract

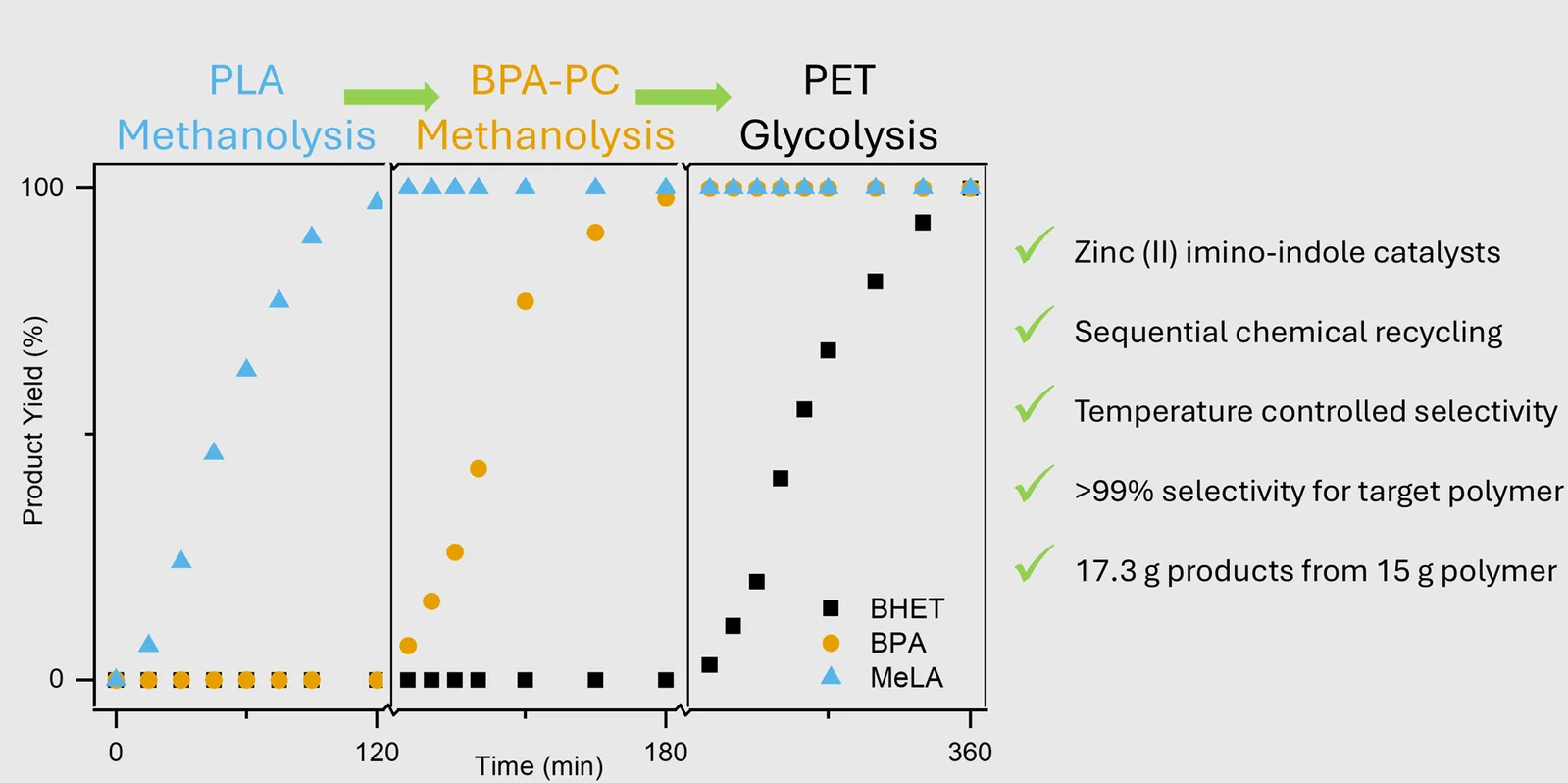

**Green foundation**

1. This work demonstrates the exceptional selectivity that can be achieved using zinc complexes in neat alcohol for selective polymer recycling; this is a key aspect of making chemical recycling tolerant of mixed-waste feedstocks and reducing the need for intensive chemical separations.

2. We have demonstrated the sequential degradation of a three-component polymer mix with >99% selectivity in each step. We have also demonstrated that we can scale this process to produce 17.3 g of total product from 15 g of waste polymer.

3. Further work is required to demonstrate this process at a larger scale, which would also allow for a detailed Life Cycle Assessment (LCA) and Techno-Economic Analysis (TEA) to be conducted.

## Introduction

Plastics are highly valued for their versatility and exceptional properties across various applications and have found use in almost every aspect of modern life.^1,2^ Despite the undeniable benefits plastics have given to society, there are serious consequences to the continued extraction of fossil resources for the polymer industry and the volume of waste that is produced from these materials.^3–5^ Approximately 8% of fossil resources that are extracted today are used as feedstock for plastic production resulting in an annual output of plastic in excess of 400 million tons.^6,7^ These levels of production and consumption are estimated to result in 509 million tons of plastic waste accumulating in marine environments by 2060.^6^ This illustrates the urgent need to develop a more sustainable future for the polymer industry.

Chemical recycling is a crucial aspect of a circular approach to plastics.^8,9^ It refers to a range of processes wherein plastic waste is broken down chemically into repolymerizable monomers (known as depolymerization, closed-loop recycling or chemical recycling-to-monomer (CRM)) or other small molecules (degradation, upcycling or open-loop recycling).^10–14^ In contrast to the mechanical recycling of plastic waste, which carries an unavoidable loss of material properties, closed-loop chemical recycling has the potential for nearly unlimited cycles of high-value material.^15,16^ Conversely, open-loop recycling seeks to retain or enhance the value of the original material through upgrading to value-added chemicals. Polymers containing heteroatoms in the backbone are ideal candidates for chemical recycling as the carbonyl-carbons are susceptible to nucleophilic attack.^17^ Well established examples include polyesters such as poly(lactic acid) (PLA)^18–24^ and poly(ethylene terephthalate) (PET);^25–27^ polycarbonates such as poly(bisphenol A carbonate) (BPA-PC);^24–28^ and polyurethanes (PU).^29,30^

The development of active and selective catalysts is a crucial component of research in this area, and zinc complexes are particularly effective for the chemical recycling of polyoxygenates.^18,20,23,28,31–33^ This has been attributed to an optimal degree of Lewis acidity in a recent comparative study.^34^ Numerous imino-phenolate zinc complexes have been reported for the open-loop recycling of PLA to give methyl lactate (MeLA), a promising green solvent and platform molecule. The most successful was capable of full PLA conversion in 30 minutes at 50 °C.^18^ An amino-phenolate example catalyzed the alcoholysis of PLA, various polycarbonates and PET to bis-hydroxyethyl terephthalate (BHET).^28^ Imino-pyrrole ligands have been used with zinc^24^ and with magnesium for PLA methanolysis and closed-loop depolymerization to lactide.^35^ The latter process was developed by the Williams group as a method for producing pure lactide from waste PLA using Sn(Oct)_2_ and a non-volatile alcohol to significantly enhance transesterification to monomer.^36^ The Herres-Pawlis group have reported PLA alcoholysis with guanidine^37^ and bisguanidine {NN}^22^ zinc(ii) catalysts. These examples all use pure samples of a single polymer, however the reality of plastic waste is that far more complex mixtures are inevitable, and all forms of recycling must account for this.^38^

Established mechanical recycling processes do not readily tolerate mixed waste streams and therefore require expensive and time-consuming sorting to ensure feedstock purity.^39^ Chemical recycling can have similar limitations, and accordingly, there has been a recent focus on designing selective chemical recycling processes for the degradation of mixed polymer waste to reduce the need for sorting.^40^ Wang *et al.* highlighted the important distinction between “one pot” and sequential recycling.^41^ The former implies the complete depolymerization of all components in the mixture, followed by a process of separation. The latter denotes a stepwise reaction where each polymer is fully converted, and products removed before the next polymer is targeted, significantly reducing the complexity of separation. This typically involves a change in reaction conditions, such as temperature modulation, to target each polymer in the mixture.^41^ Saito *et al.* also used temperature to control selectivity and were able to sequentially convert a four-component mixture (BPA-PC/PU/PET/polyamide) using a salt of the common organic superbase TBD.^42^ A recent report from the Herres-Pawlis group also employed an organocatalyst to sequentially convert PLA, poly-ε-caprolactone (PCL) and PET, showing excellent selectivity through temperature control.^43^ The selectivity of sequential chemical recycling can also be controlled through catalyst choice. One example is the three-component system reported by the Dove group where PLA, BPA-PC and PET were selectively depolymerized through careful selection of catalyst and solvent choice tailored for each plastic.^44^ Polyoxymethylene (POM) has also shown selectivity with BPA-PC and PET using the appropriate choice of catalyst and solvent.^45^ A “one-pot” example of PLA/BPA-PC degradation used a functionalized ionic liquid to facilitate effective separation of the respective products.^46^ Ma and co-workers described a process for analyzing and quantifying real-life mixtures of nine plastics using in-line NMR and converting particular components of the mixture in sequence to obtain valuable chemicals.^47^ They were ultimately able to recover 12.4 g of valuable chemicals from 20 g of plastic waste, representing an important step towards the valorization of real-world plastic waste.

We recently reported low-temperature chemical recycling of PLA and BPA-PC in methanol, and the sequential methanolysis of these two polymers with selectivity modulated by temperature.^24^ Low levels of BPA-PC consumption in the PLA methanolysis step persisted, leading to low but significant levels of product contamination. Herein, we report a new class of ligands with similar coordination profiles and increased steric bulk with the aim of developing a three-component sequential system that is fully selective for PLA, BPA-PC and PET at different temperatures using a single catalyst.

To this end, four air-stable zinc complexes based on 7-formyl indole derived ligands are reported and applied to the co-solvent-free alcoholysis of PLA, BPA-PC and PET including reactions in air and with different alcohols. The sequential degradation of PLA with BPA-PC is optimized and expanded to include PET with the goal of obtaining kinetic data and isolating products through the production of robust zinc catalysts that are capable of exceptional selectivity in mixed-plastic waste streams.

## Experimental

### Chemicals and equipment

All chemicals were obtained commercially from Sigma-Aldrich and used as received with the exception of PLA cups and PET bottles which were commercially available and produced by Vegware® and Coca-Cola® respectively. All ligands were prepared using standard literature procedures in air. All metal complexes were synthesized under an inert atmosphere (argon) using standard Schlenk line techniques, dry solvents, and oven-dried glassware. Complexes were stored in air.

Electrospray ionization mass spectrometry (ESI-MS) was recorded for all ligands using Agilent 6545 ESI quadrupole time-of-flight, fitted with liquid chromatography module. Each spectrum was recorded in positive loop injection mode. Sample was dissolved in methanol at approximately 1 µg mL^−1^.

Crystallographic data for two complexes were collected on a SuperNova or Excalibur, EOS detector diffractometer using Cu-Kα (*λ* = 1.54184 Å) or Mo-Kα (*λ* = 0.71073 Å) radiation, all recorded at 150(2) K. Both structures were solved by direct methods and refined on all *F*^2^ data using the SHELXL-2014 suite of programs.

### General procedures

#### Typical ligand synthesis

7-Formyl indole (1.452 g, 1 equiv., 10 mmol) and diamine (10 mmol) were added to a round bottomed flask with MeOH (30 mL). The reaction was stirred at room temperature for 24 h and the solution went from colorless to yellow. The solvent was removed *in vacuo* giving yellow oils as products, 1–4H (70–86%).

#### Typical complex synthesis

Ligands 1–4H (2 equiv., 4 mmol) were dried *in vacuo* for two hours and dissolved in anhydrous toluene (10 mL) in a Schlenk flask. Zn(Et)_2_ (2 mL, 1 equiv., 2 mmol) was added dropwise and the solution was stirred overnight before removal of toluene *in vacuo* to give a yellow solid. The crude product was crystallized from varying amounts of toluene and hexane (12–49%).

#### Typical PLA alcoholysis in THF procedure

A Young's ampoule containing PLA (0.25 g, Vegware™ PLLA cup, *M*_n_ = 45 510 g mol^−1^), was loaded with metal complex (8 wt%, 0.02 g). The polymer was dissolved in THF (4 mL) with heating and stirring assisting dissolution. The flask was then submerged in a preheated oil bath (80 °C) to which MeOH (1 mL, 7 eq. with respect to ester group) was added. Aliquots were taken for ^1^H NMR (CDCl_3_) analysis of the methine region. For kinetics experiments, aliquots were taken at the appropriate timepoints and analysed as described above.

#### Typical BPA-PC methanolysis in THF procedure

A Young's ampoule containing BPA-PC (0.25 g, pellets, *M*_n_ ≈ 45 000 g mol^−1^) was loaded with metal complex (4 wt%, 0.01 g). The polymer was dissolved in THF (4 mL) with heating and stirring assisting dissolution. The flask was then submerged in a preheated oil bath (75 °C) to which MeOH (17.5 eq. with respect to carbonate groups) was added. Aliquots were taken for ^1^H NMR (CDCl_3_) of the methyl region. BPA was recrystallised from water and dried to a constant weight to determine isolated yields.

#### Typical co-solvent-free PLA methanolysis procedure

A Young's ampoule containing PLA (0.25 g, Vegware™, PLLA cup, *M*_n_ = 45 510 g mol^−1^), was loaded with metal complex (1–16 wt%, 0.0025–0.04 g). The flask was then submerged in a preheated oil bath (60–130 °C) to which MeOH (2 mL, 14 eq. with respect to ester group) was added. When the PLA had been fully consumed, an aliquot was taken for ^1^H NMR (CDCl_3_) analysis of the methine region. Reactions in air were performed in a round bottom flask with a reflux condenser attached using the conditions described above. For reactions using ethanol, the 14 : 1 molar ratio of alcohol to ester group was maintained.

#### Typical co-solvent-free BPA-PC methanolysis procedure

A Young's ampoule containing BPA-PC (0.25 g, pellets: *M*_n_ ≈ 45 000 g mol^−1^) was loaded with metal complex (4 wt%, 0.01 g). The flask was then submerged in a preheated oil bath (80–130 °C) to which MeOH (2 mL, 50 eq. with respect to carbonate group) was added. When the polymer had been fully consumed, an aliquot was taken for ^1^H NMR (CDCl_3_). Aliquots were taken for ^1^H NMR (CDCl_3_) of the methyl region. BPA was recrystallised from water and dried to a constant weight to determine isolated yields.

#### Typical PET glycolysis procedure

A Young's ampoule containing PET (0.25 g of carbonated drinks bottle) was loaded with metal complex (8 wt%, 0.02 g). Ethylene glycol (EG) (27.5 eq., 1.5 mL) was added, and the flask was submerged in a pre-heated oil bath at 180 °C. When full disappearance of the PET was observed, water was added, and the mixture was filtered. BHET crystallized from the mixture and was collected, dried at 100 °C *in vacuo* for 4 hours and weighed to obtain isolated yields. When required, an aliquot was taken and analysed with a 1,3,5-trimethoxybenzene internal standard to obtain spectroscopic BHET yield.

#### Typical mixed-plastic optimization reaction

A Young's ampoule containing PLA (0.125 g, Vegware™ PLLA cup, *M*_n_ = 45 510 g mol^−1^) and BPA-PC (0.125 g, pellets, *M*_n_ ≈ 45 000 g mol^−1^) was loaded with metal complex (2–16 wt% with respect to PLA, 0.0025–0.02 g). The flask was then submerged in a preheated oil bath (70–130 °C) to which MeOH (2 mL, 14 equiv. with respect to ester group) was added. When the PLA had been fully consumed, the reaction was filtered to remove unreacted BPA-PC, and a suitable solvent was added to the filtrate to ensure dissolution of the mixture. An aliquot was taken for ^1^H NMR (CDCl_3_) analysis of the methine region for MeLA and the methyl region for BPA. Unreacted BPA-PC was weighed and dried *in vacuo* to determine mass loss.

#### Typical three-component sequential chemical recycling experiment

24 individual reactions were carried out. Each Young's ampoule containing PLA (0.1 g, Vegware™ PLLA cup, *M*_n_ = 45 510 g mol^−1^), BPA-PC (0.1 g pellets, *M*_n_ ≈ 45 000 g mol^−1^) and bottle-grade PET (0.1 g *M*_n_ ≈ 40 000 g mol^−1^) was loaded with metal complex (8 wt% with respect to PLA, 8 mg, 1.26 mol% relative to ester linkages) and MeOH (1.6 mL, *n*_MeOH_ : *n*_ester_ = 14 : 1).

#### PLA methanolysis

The reaction was heated to 70 °C for the allotted time. Unreacted polymer was filtered, separated by type, dried and weighed to determine mass loss of PLA, BPA-PC and PET. An aliquot was taken for ^1^H NMR (CDCl_3_) analysis of the methine region for MeLA and the methyl region for BPA.

#### BPA-PC methanolysis

The reaction was heated to 70 °C for two hours then heated to 100 °C for the allotted time. Unreacted polymer was filtered, separated by type, dried and weighed to determine mass loss of BPA-PC and PET. An aliquot was taken for ^1^H NMR (CDCl_3_) analysis of the methyl region for BPA.

#### PET glycolysis

The reaction was heated to 70 °C for two hours then heated to 100 °C for one hour. After this time, volatiles were removed and EG (1.2 mL, *n*_MeOH_ : *n*_ester_ = 20.6 : 1) added. The reaction was heated to 180 °C for the allotted time. Unreacted polymer was filtered, dried and weighed to determine mass loss of PET. An aliquot was taken for ^1^H NMR (CDCl_3_) analysis with an internal standard to quantify BHET yield.

#### Large scale three-component chemical recycling

A Young's ampoule containing PLA (5 g, Vegware™ PLLA cup, *M*_n_ = 45 510 g mol^−1^), BPA-PC (5 g pellets, *M*_n_ ≈ 45 000 g mol^−1^) and bottle-grade PET (5 g *M*_n_ ≈ 40 000 g mol^−1^) was loaded with metal complex (4 wt% with respect to PLA, 200 mg, 0.63 mol% relative to ester linkages) and MeOH (40 mL, *n*_MeOH_ : *n*_ester_ = 14 : 1). The reaction was heated to 70 °C for three hours after which unreacted methanol (27.32 g, 93% recovery) was removed through room-temperature vacuum distillation. Methyl lactate (9.21 g, 50 mol% solution in MeOH, 97% yield) was collected through vacuum distillation at 70 °C.

Recovered methanol was added back to the reaction flask and the mixture was heated to 100 °C for one hour. After this time, unreacted PET was collected *via* filtration, and the reaction mixture was distilled to give a solution of DMC in MeOH (20.96 g) and a red solid which was recrystallised from water to give BPA (4.03 g, 88% yield).

Unreacted PET, EG (30 mL, *n*_MeOH_ : *n*_ester_ = 20.6 : 1) and metal complex (8 wt% with respect to PET, 400 mg, 3.30 mol% relative to ester linkages) were added to the Young's Ampoule. The reaction was heated to 180 °C for two hours. When full disappearance of the PET was observed, water was added, and the mixture was filtered. BHET crystallized from the mixture and was collected, dried at 100 °C *in vacuo* for 4 hours and weighed to obtain isolated yield (6.17 g, 93%). An aliquot was taken and analysed with a 1,3,5-trimethoxybenzene internal standard to obtain spectroscopic BHET yield.

## Results and discussion

### Polymer methanolysis in THF

**Fig. 1:**
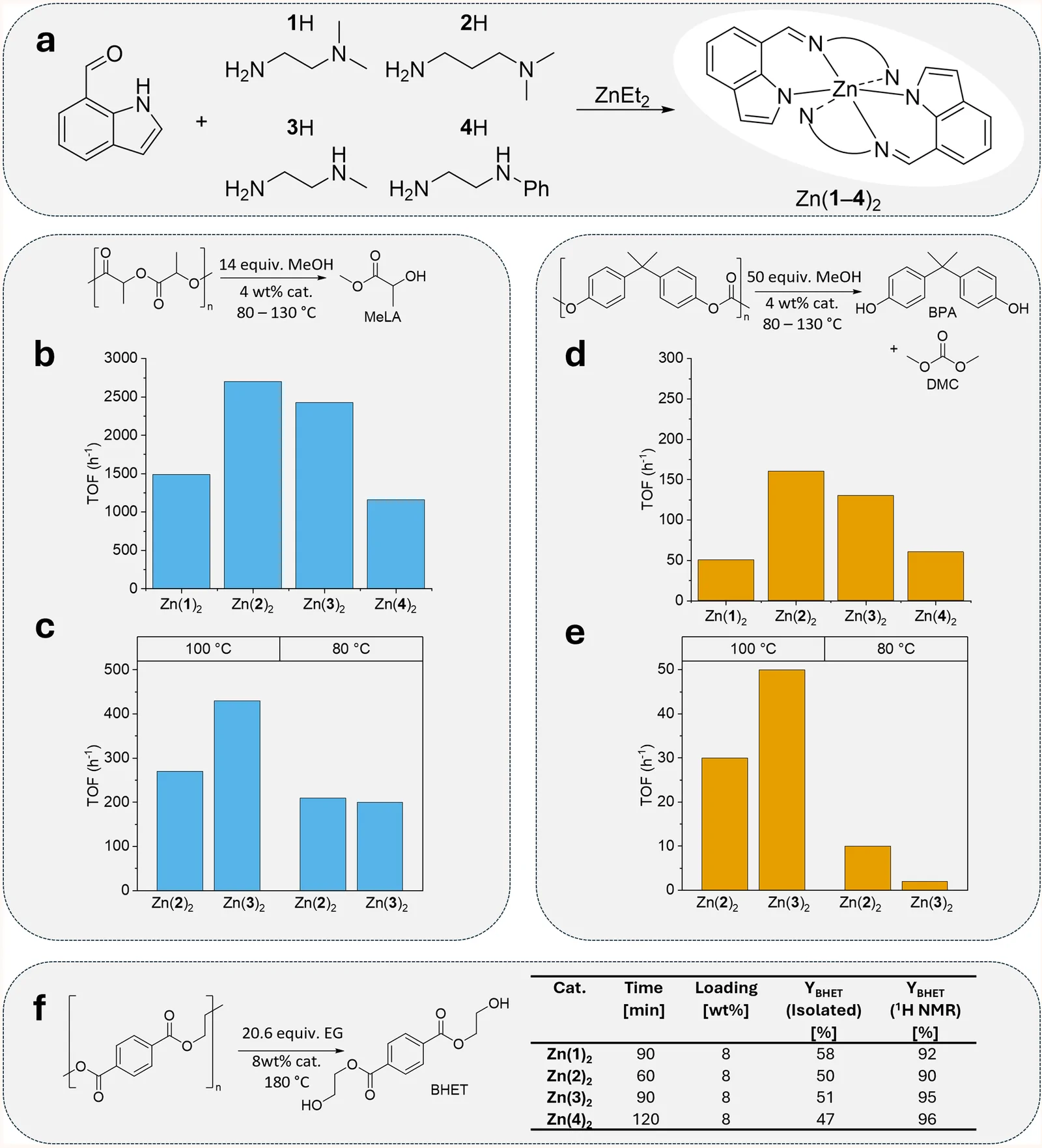
(a) Structure of Zn(1–4)_2_. (b) PLA methanolysis in neat methanol with Zn(1–4)_2_ at 130 °C for 4–6 min. Conditions: 0.25 g of PLLA cup (*M*_n_ = 45 510 g mol^−1^), MeOH (2 mL, *n*_MeOH_ : *n*_ester_ = 14 : 1), 4 wt% cat. loading (10 mg, 0.49–0.63 mol% relative to ester linkages). (c) PLA methanolysis in neat methanol with Zn(2–3)_2_ at 80–100 °C for 21–60 min. Conditions: 0.25 g of PLLA cup (*M*_n_ = 45 510 g mol^−1^), MeOH (2 mL, *n*_MeOH_ : *n*_ester_ = 14 : 1), 4 wt% cat. loading (10 mg, 0.56–0.63 mol% relative to ester linkages). (d) BPA-PC methanolysis in neat methanol with Zn(1–4)_2_ at 130 °C for 20–60 min. Conditions: 0.25 g BPA-PC (pellets: *M*_n_ ≈ 45 000 g mol^−1^), MeOH (2 mL, *n*_MeOH_ : *n*_carbonate_ = 50 : 1), 4 wt% cat. loading (10 mg, 1.72–2.18 mol% relative to carbonate linkages). (e) BPA-PC methanolysis in neat methanol with Zn(2–3)_2_ at 80–100 °C for 60 min. Reaction conditions: 0.25 g BPA-PC (pellets: *M*_n_ ≈ 45 000 g mol^−1^), MeOH (2 mL, *n*_MeOH_ : *n*_carbonate_ = 50 : 1), 4 wt% cat. loading (10 mg, 1.95–2.18 mol% relative to carbonate linkages). (f) PET glycolysis in neat EG with Zn(1–4)_2_ at 180 °C for 60–120 min. Reaction conditions: 0.25 g of bottle-grade PET (*M*_n_ ≈ 40 000 g mol^−1^), EG (1.5 mL, *n*_EG_ : *n*_ester_ = 20.6 : 1), 8 wt% cat. loading (20 mg, 2.60–3.30 mol% relative to ester linkages).

Ligands were prepared through imine-condensation and structures varied by amine linker (X = ethyl, propyl) and pendant amine substituent (R = H, Me, Ph). This was followed by complexation with diethyl zinc to give the catalysts, Zn(1–4)_2_ (Fig. 1a), full characterization details can be found in the SI (Fig. S1–S21). Complexes Zn(1–4)_2_ were initially tested for the degradation of PLA to MeLA in tetrahydrofuran (THF) with 8 wt% catalyst loading at 80 °C (Table S1).

Zn(2)_2_ and Zn(3)_2_ were the quickest catalysts to reach high PLA conversion after 90 min and 60 min respectively. Increasing the size of the amine bridge from ethyl, Zn(1)_2_, to propyl, Zn(2)_2_, had a positive effect on activity. This trend has been noted with imino-phenolate, amino-phenolate and imino-pyrrole complexes and could be attributed to the increased flexibility of the ligand offering a more dynamic coordination sphere for polymer interaction.^18,24,28,48^ The comparison between the two ethyl-linked, secondary amine catalysts, Zn(4)_2_ (R′ = Ph) and Zn(3)_2_ (R′ = Me) suggests that increasing the steric bulk of the amine reduces activity. However, Zn(4)_2_ was noticeably more active than Zn(1)_2_ (X = –CH_2_CH_2_–, R = R′ = Me) which implies the inclusion of an N–H group partially outweighs the increase in steric hindrance (Table S1).

Pseudo-first order kinetic data was obtained for PLA methanolysis, in keeping with literature examples (Fig. S23).^28,49^ At the same conditions, the most active imino-pyrrole catalyst (*k*_app_ = 0.365 min^−1^)^24^ was an order of magnitude faster than Zn(2)_2_ (*k*_app_ = 0.047 min^−1^) and Zn(3)_2_ (*k*_app_ = 0.042 min^−1^).^24^ The difference in activity can likely be related to the increased steric bulk of the indole moiety compared with pyrrole. Zn(1)_2_ and Zn(4)_2_ were the least active with apparent rate constants of 0.007 min^−1^ and 0.019 min^−1^ respectively. These catalysts are also considerably slower than the most active {ONN} imino-phenolate examples (*k*_app_ ≤ 0.20 min^−1^) which were also run at lower temperature and catalyst loadings.^18^

A similar trend in activity was observed for the methanolysis of BPA-PC (Table S2, Fig. S24) using Zn(1–4)_2_ and previously reported conditions (4 wt% catalyst, 2 mL THF, 17.5 equiv. MeOH, one hour).^24,28^ Zn(1)_2_ and Zn(4)_2_ both gave less than 50% conversion to BPA and efforts to obtain an isolated yield were unsuccessful due to the presence of side-products. However, Zn(2)_2_ and Zn(3)_2_ gave good selectivity to BPA after one hour and isolated yields of 72% and 80% respectively.

### Polymer alcoholysis in neat alcohol

Reducing reliance on toxic solvents is an important aspect of green chemistry and all complexes were shown to be active for the methanolysis of PLA in neat methanol at 130 °C using 4 wt% Zn(1–4)_2_ (Fig. 1, Fig. S22). Due to the insolubility of PLA in methanol, reactions were run until fully homogeneous. In all cases, this occurred in six minutes or less. The best performing catalyst, Zn(3)_2_ reached full conversion in four minutes. As obtaining kinetic data is challenging with these reactions, turnover frequency (TOF) values were used to compare results while considering differing conversions, reaction times and catalyst loadings. In keeping with the degradation of PLA in THF, Zn(2)_2_ and Zn(3)_2_ were the most active catalysts giving TOF values of 2700 h^−1^ and 2430 h^−1^ respectively (Fig. 1b). Zn(4)_2_ was the slowest, converting 33% of internal methine after four minutes. Zn(3)_2_ is comparable to its imino-pyrrole counterpart which gave full PLA conversion in one minute under the same conditions.^24^ Furthermore, both pyrrole and indole-based catalysts significantly outperform previous examples from the literature.^18,20,31^

To assess the activity of these catalysts under mild conditions, and to observe any change in selectivity, Zn(2)_2_ and Zn(3)_2_ were tested for PLA degradation in neat methanol at 100 °C and 80 °C (Fig. 1c). At 100 °C, high conversion and homogenisation of the reaction mixture was achieved after 30 minutes for Zn(2)_2_ and 21 minutes for Zn(3)_2_ giving TOF values of 270 h^−1^ and 430 h^−1^ respectively. Good catalytic activity was also shown at 80 °C with TOF values of 210 h^−1^ for Zn(2)_2_ and 200 h^−1^ for Zn(3)_2_. The reaction took 45–60 minutes to reach homogeneity, corresponding to high PLA conversion. This is longer than for the best performing imino-pyrrole catalyst but still represents a highly-active catalyst for this reaction.^24^ To assess the sensitivity of this reaction to air and moisture, the 80 °C methanolysis reactions were repeated in a round bottom flask at reflux (Table S3). This gave reduced conversion and yield, likely a combination of air/moisture sensitivity and the effective reaction temperature at the boiling point of methanol. The ethanolysis of PLA was attempted at 130 °C in neat ethanol (Table S4). The polymer was more readily solubilised upon heating than with methanol at the same temperature, presumably a result of decreased polarity. After one hour, Zn(2)_2_ and Zn(3)_2_ had converted 48% and 81% of internal methine to give 11% and 37% yields of ethyl lactate (EtLA) respectively.

Catalyst recycling was demonstrated for neat PLA methanolysis with Zn(3)_2_. Over an initial five runs, the catalyst remained active but activity reduced after each run. An additional portion of catalyst was added before run 6, which mostly restored activity before again reducing with each run. This shows that Zn(3)_3_ is less reusable than the equivalent pyrrole and similar to other homogeneous catalysts in the literature.^24,50^

All catalysts were tested for the methanolysis of BPA-PC at 130 °C in neat methanol (Fig. 1d). Zn(1)_2_ and Zn(4)_2_ were again the slowest, taking one hour to reach high BPA yields of 95% and 79% respectively. Zn(2)_2_ was marginally more active than Zn(3)_2_ with a TOF value of 160 h^−1^ although both catalysts were capable of complete solubilisation of the reaction mixture in around 20 minutes, corresponding to full deconstruction of BPA-PC. Reactions at lower temperatures were conducted using the most effective catalysts, Zn(2)_2_ and Zn(3)_2_. Reasonable activity was maintained at 100 °C with TOF values of 30 h^−1^ And 50 h^−1^ respectively (Fig. 1e). At 80 °C, minimal activity was shown for Zn(3)_2_ which achieved a mass loss of 5% and a TOF value of 2 h^−1^, significantly lower than equivalent pyrrole complex.^24^ This result shows that Zn(3)_2_ exhibits a large difference in activity between BPA-PC and PLA at 80 °C, crucial for achieving the selective chemical recycling of this mixture.

The glycolysis of PET was carried out at 180 °C in neat ethylene glycol to give the repolymerisable monomer BHET (Fig. 1f). All catalysts were active for glycolysis with reaction times between 60–120 min. BHET yields of 90–96% were measured using ^1^H NMR spectroscopy with an internal standard of which 47–58% could be isolated due to inefficiencies in small scale recrystallisation. In keeping with the PLA and BPA-PC results, Zn(2)_2_ and Zn(3)_2_ were the most active examples. The high temperatures required for glycolysis are ideal for the incorporation of PET into a sequential system where the other polymers are converted at much lower temperatures. The produced BPA and BHET showed some red colouration after recrystallisation, however the high purity showed by ^1^H NMR suggests that these products would be repolymerisable (Fig. S27 and S28).

### Chemical recycling to monomer

Although it is possible to go from MeLA to lactide monomer there are distinct advantages in developing CRM methods to produce high-purity lactide directly from waste material.^51^ The chemical recycling of PLA to lactide catalysed by Zn(1–4)_2_ was investigated using the reactive sublimation conditions published by Williams *et al.* using a Sn(Oct)_2_ and glycerol ethoxylate (GEO) system (Table 1).^36^ Two blank experiments containing GEO and PLA with no catalyst showed very little activity after five hours and a lactide yield of 56% after 24 hours, which corresponded to a reduction in molecular weight of the polymer residue (Fig. S29). A catalyst screen ([Cat]/[OH_GEO_]/[PLA] = 1 : 20 : 1000) showed that Zn(2)_2_ and Zn(3)_2_ were the most active, in keeping with the methanolysis results, giving lactide yields of 96% and 93% respectively. Using Zn(2)_2_, the catalyst loading was reduced ([Cat]/[OH_GEO_]/[PLA] = 1 : 80 : 4000) with minimal loss of activity to give a 96% lactide yield in three hours, showing activity that is competitive with Sn(Oct)_2_ under these conditions.^36^

**Table 1:** PLA depolymerization to l-lactide using Zn(1–4)_2_ at 160 °C *in vacuo*^a^

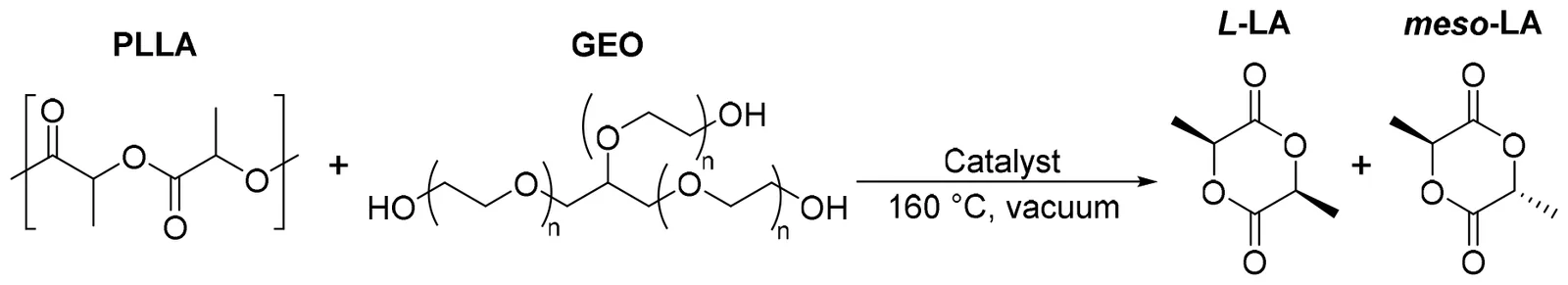				
Cat.	Ratio [Cat] : [OH_GEO_] : [PLA]	Time [h]	Yield^a^ [%]	l-LA^b^ [%]
None	0 : 20 : 1000	5	1	—
None	0 : 20 : 1000	24	56	97
Zn(1)_2_	1 : 20 : 1000	2	73	93
Zn(2)_2_	1 : 20 : 1000	2	96	94
Zn(3)_2_	1 : 20 : 1000	2	93	92
Zn(4)_2_	1 : 20 : 1000	2	10	98
Zn(2)_2_	1 : 40 : 2000	2.75	95	94
Zn(2)_2_	1 : 80 : 4000	3	96	93
Zn(2)_2_	1 : 320 : 16 000	24	89	93
Zn(2)_2_	1 : 640 : 32 000	24	92	94

a Isolated l-LA yield.

b l-LA content determined *via* integration of methyl resonance of ^1^H NMR spectrum.

The catalyst loading was further decreased to [Cat]/[PLA] = 1 : 16 000 and 1 : 32 000 whilst maintaining a 1 : 50 ratio of OH_GEO_ to PLA throughout. Both experiments gave high lactide yields after 24 hours with the latter experiment giving 92% l-lactide. The 24-hour blank suggests that some conversion could be attributed to GEO at this timepoint, however there is still a significant increase in lactide that can be attributed to the addition of ppm levels of catalyst. The percentage of l-lactide was measured by the ratio of methyl signals in the ^1^H NMR spectrum and was shown to be greater than 90% throughout. This suggests limited epimerization to the *meso* form, particularly when considering that the starting material contains approximately ≈95% PLLA.

The lactide produced from Zn(2)_2_ was combined and singly recrystallised from anhydrous toluene. It was subsequently polymerised at a 100 : 1 : 1 ratio with a literature Zn(ii) catalyst and benzyl alcohol at 130 °C in the melt.^18^ A conversion of 92% was attained after two hours, demonstrating the circularity of this approach. However, the molecular weight of the polymer was low (*M*_n SEC_ = 1100 g mol^−1^, *Ð* = 1.30) suggesting the presence of protic impurities acting as additional co-initiators, indicating further optimisation is necessary.

### Mixed-plastic optimization

**Fig. 2:**
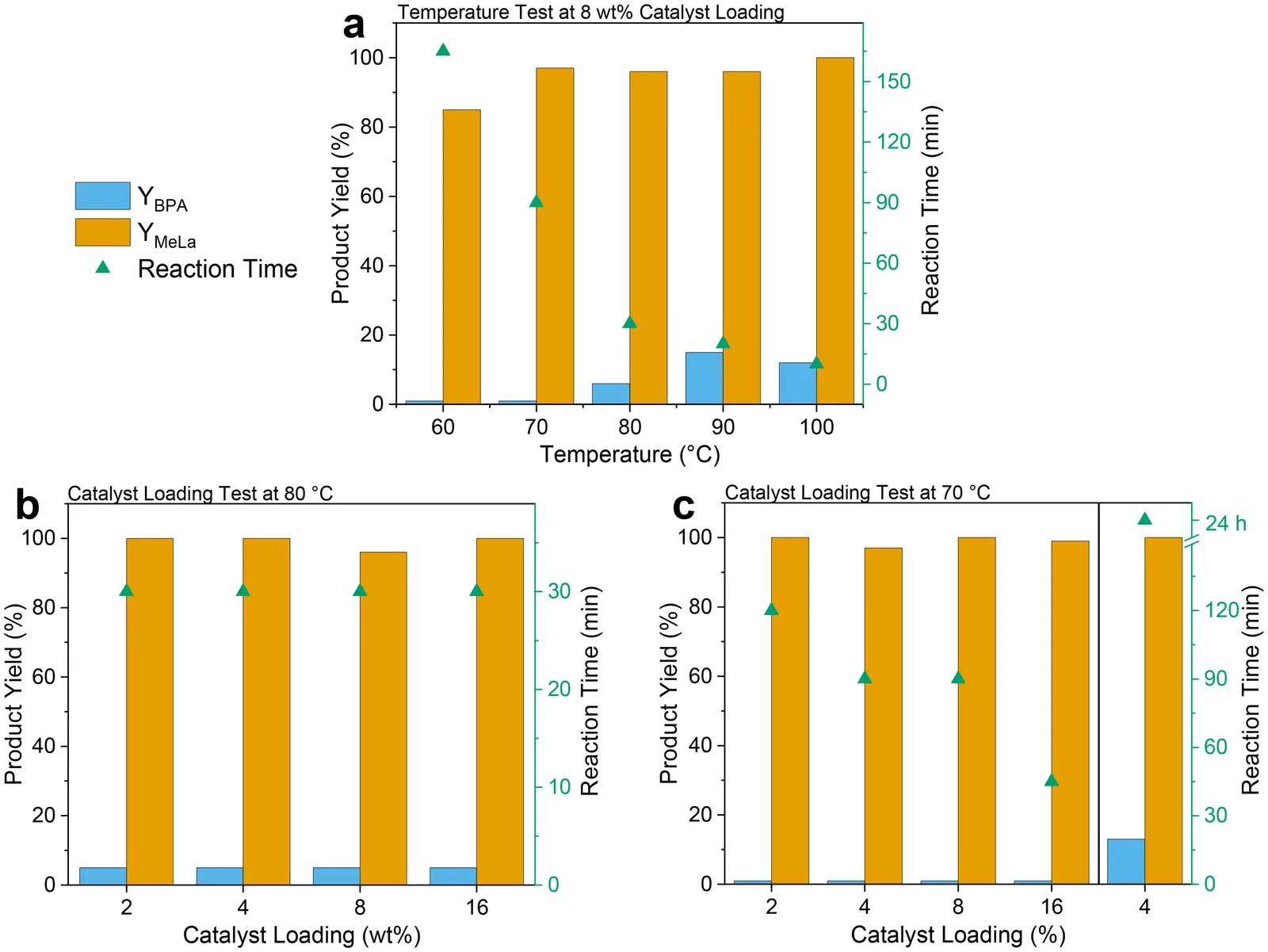
PLA/BPA-PC selectivity optimization using Zn(3)_2_ at 60–100 °C under argon. (a) Temperature comparison with 8 wt% Zn(3)_2_. (b) Catalyst loading comparison at 80 °C. (c) Catalyst loading comparison at 70 °C. General conditions: 0.125 g of PLLA cup (*M*_n_ = 45 510 g mol^−1^), 0.125 g BPA-PC (pellets: *M*_n_ ≈ 45 000 g mol^−1^), 2–16 wt% cat. loading with respect to PLA (2.5–40 mg, 0.31–2.51 mol% relative to ester linkages), MeOH (2 mL, *n*_MeOH_ : *n*_ester_ = 14 : 1). Reaction stopped upon disappearance of PLA. MeLA yield calculated from integration of the methine region of the ^1^H NMR spectrum. BPA yield calculated from the product of mass loss (%) and selectivity to BPA (%) divided by 100.

Optimizing for highly selective sequential depolymerization is a major challenge. For example, in our recently reported sequential methanolysis of PLA and BPA-PC, it was noted that during the initial PLA methanolysis, a small amount (5–7%) of BPA-PC was also converted.^24^ Therefore, a series of experiments were conducted to optimize the selectivity of this sequential reaction using Zn(3)_2_ (Fig. 2, Tables S5–S7). A mixture of PLA and BPA-PC was reacted with Zn(3)_2_ in methanol until no PLA was observable. The remaining BPA-PC was weighed to determine mass loss, and an aliquot was taken for ^1^H NMR analysis to determine PLA conversion (*X*_int_), MeLA yield (*Y*_MeLA_), selectivity to BPA (*S*_BPA_) and BPA yield (*Y*_BPA_).

The effect of temperature was initially investigated (Fig. 2a). The reaction time decreased as the temperature was increased from 60 °C (165 min) to 100 °C (15 min). However, significant mass loss of BPA-PC was observed at temperatures greater than 80 °C. At 80 °C, 6% mass loss was observed, in keeping with the results from imino-pyrrole complexes.^24^ This suggests that BPA-PC conversion could be a function of BPA-PC solubility in methanol at different temperatures as opposed to intrinsic catalyst selectivity. Below 80 °C, BPA-PC lost less than 1% of its mass, although at 60 °C the reaction time increased to 100 minutes, and MeLA yield only reached 85%. 70 °C and 80 °C were therefore selected for further investigation into the effect of catalyst loading (Fig. 2b and c).

At 80 °C (Fig. 2b), the catalyst loading had no significant effect on catalytic activity in terms of PLA conversion (99–100%), reaction time (30 min) and mass loss of BPA-PC (5–6%). This supports the hypothesis that the conversion of BPA-PC depends upon a small amount of solubility at this temperature. The same series of experiments conducted at 70 °C (Fig. 2c) resulted in longer reaction times (45–120 min) and gave <1% BPA yield at full PLA conversion regardless of catalyst loading. This demonstrates the capability of orthogonal PLA/BPA-PC methanolysis with near-perfect selectivity. After 24 hours with 4 wt% Zn(3)_2_, a 15% mass loss of BPA-PC was observed. This demonstrates that control of reaction time is important to avoid unwanted BPA formation during the first stage of the reaction, however the timescale is significantly greater than the PLA degradation.

### Three component mixed plastic recycling

**Fig. 3:**
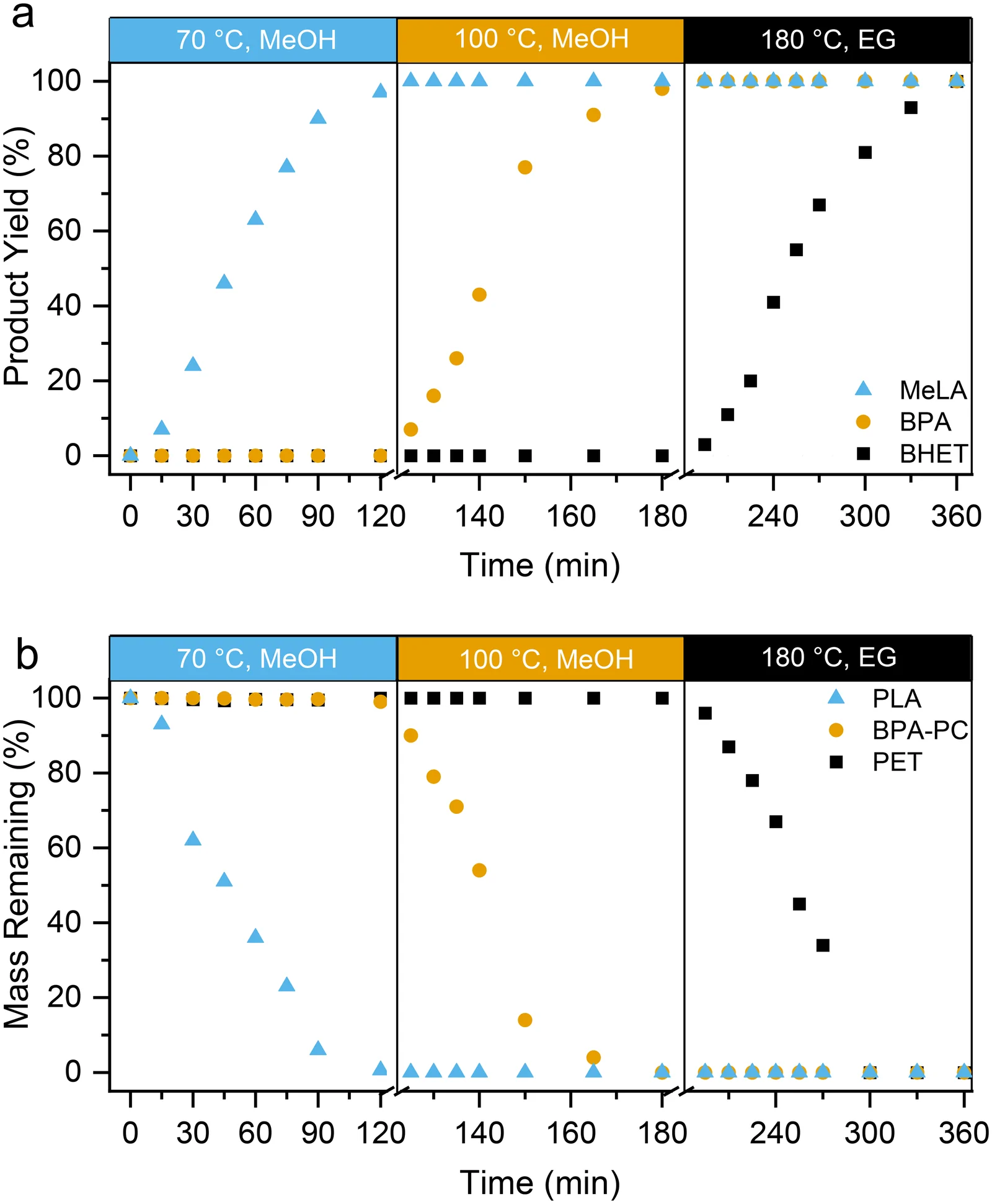
Sequential chemical recycling of PLA, BPA-PC and PET. (a) Product yields over time. Determined by ^1^H NMR spectroscopy with an internal standard used for BHET quantification. (b) Mass remaining over time. After each reaction, remaining polymer was dried, separated and weighed to determine mass loss. Conditions (PLA methanolysis): 0.1 g of PLLA cup (*M*_n_ = 45 510 g mol^−1^), 0.1 g BPA-PC (pellets: *M*_n_ ≈ 45 000 g mol^−1^), 0.1 g of bottle-grade PET (*M*_n_ ≈ 40 000 g mol^−1^), 8 wt% cat. loading with respect to PLA (8 mg, 0.63 mol% relative to PLA ester linkages), MeOH (1.6 mL, *n*_MeOH_ : *n*_ester_ = 14 : 1 w.r.t PLA), 70 °C, 0–120 min. Conditions (BPA-PC methanolysis): PLA methanolysis conditions for 120 min then 100 °C for 5–60 min. Conditions (PET glycolysis): PLA methanolysis conditions for 120 min, BPA-PC methanolysis conditions for 60 min then volatiles removed, EG (1.2 mL, *n*_MeOH_ : *n*_ester_ = 20.6 : 1) added, 180 °C, 15–180 min.

Having demonstrated the selectivity of Zn(3)_2_ for each polymer at different temperatures, a three-component, sequential system could be constructed for PLA, BPA-PC and PET using optimized conditions for each polymer (Fig. 3, Table S8). With Zn(3)_2_ at 70 °C, PLA conversion and mass loss reached >99% with a with a MeLA yield of 97% after two hours. The reaction profiles of product yield and mass loss show an S-shaped curve where the reaction is slower at the outset and end with a rapid, linear phase between 20% and 90% product yield This could reflect an induction stage where catalyst and methanol diffuse into the polymer matrix before rapid deconstruction of the polymer until the concentration drops alongside the rate. Crucially, during this phase, less than 1% yield of BPA and BHET were observed. This demonstrates excellent selectivity, exceeding that of the equivalent imino-pyrrole.^24^

On heating the reaction mixture to 100 °C, BPA-PC degradation occurred rapidly giving 10% mass loss and 7% BPA yield after five minutes. The product yield plot shows activity reducing at high conversion after 30 minutes before reaching full mass loss after one hour with a BPA yield of 98%. The mass loss of PET remained below 1%, again highlighting the selectivity of this process. Furthermore, this shows that BPA-PC methanolysis is not negatively affected by residual MeLA.

Upon addition of EG and heating to 180 °C, PET glycolysis proceeded to completion over three hours. The plot displayed a similar S – shaped profile to PLA and BPA-PC for product yield; however, 100% mass loss was observed after two hours. At this point, the solutions were observed to contain solid particles that were too small to be isolated by simple filtration. This suggests that the PET significantly fragmented between 90 and 120 minutes at 180 °C and between 57% and 81% BHET yield. This could be attributed to brittleness through the increasing crystallinity of the remaining solid polymer as amorphous regions are initially consumed, as reported by Dadmun *et al.*^52^ Overall, these results demonstrate three-component, sequential chemical recycling with >99% selectivity to the target products.

**Fig. 4:**
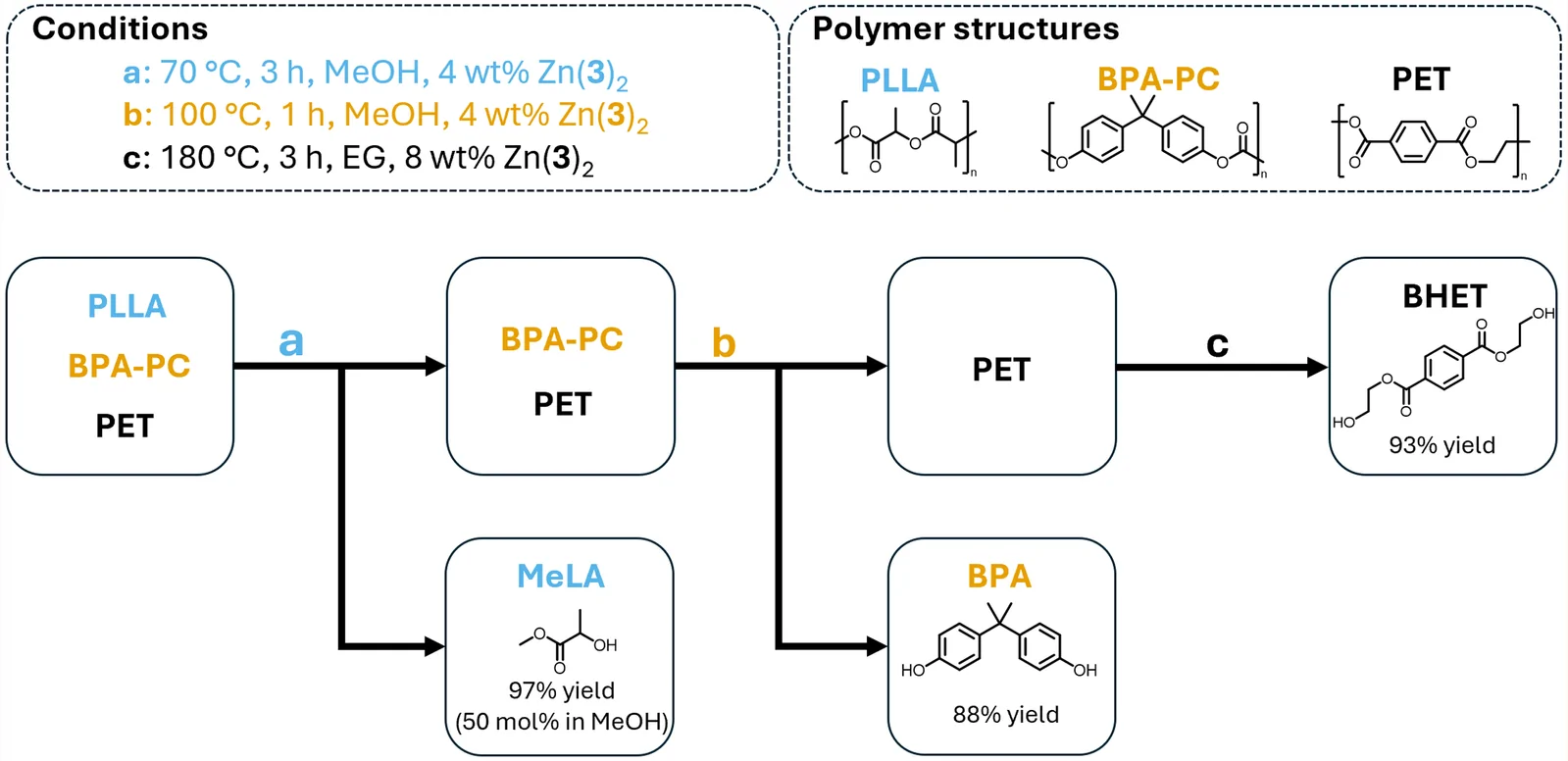
Scaled up sequential chemical recycling of PLA, BPA-PC and PET.

The optimized chemical recycling of PLA, BPA-PC and PET was scaled up to 5 g of each polymer to demonstrate viability of this process with sequential product removal (Fig. 4). PLA methanolysis was carried out at 70 °C for three hours with 4 wt% Zn(3)_2_. Upon completion, the volatile components were distilled off to give 97% methyl lactate yield as a 50 mol% solution in methanol; this is a challenging mixture to separate at lab scale. A total of 27.3 g of methanol containing <1% MeLA was also recovered, and this was used for the subsequent methanolysis reaction, enhancing sustainability and reducing the overall cost of the reaction.

After one hour at 100 °C, BPA-PC methanolysis was complete and the reaction mixture was decanted to leave unreacted PET in the reaction flask. Distillation of the volatile components resulted in 21.0 g of dimethyl carbonate (DMC)/methanol solution. Recrystallisation of the crude mixture from water resulted in 4.0 g BPA (*Y*_BPA_ = 88%), unfortunately the red coloration of the catalyst could not be fully removed from the final product.

Ethylene glycol and 8 wt% Zn(3)_2_ were added to the unreacted PET and glycolysis occurred at 180 °C yielding 6.17 g of recrystallised BHET (*Y*_BHET_ = 93%) after three hours. The BHET product also retained some red coloration even after several recrystallisations. The zinc content of singly recrystallised BHET was measured as 0.83% by ICP-OES. This could be reduced to <10 ppm by sublimation, although a small amount of red colouration remained. Overall, this experiment demonstrated the feasibility of scaling up this sequential process while retaining the selectivity demonstrated in Fig. 3, however issues around discoloration will need to be addressed in future research. Considering all these steps, including purifications and solvent recycling, the important green metrics, Process Mass Intensity (PMI) and *E*-factor were calculated. These are defined respectively as total mass of inputs divided by total mass of products (PMI) and total mass of waste divided by total mass of products (*E*-factor). They were calculated as PMI = 3.2 and *E*-factor = 4.8, however, further work is required to contextualize and optimise these metrics (Fig. S30). Ultimately, a full Life Cycle Assessment (LCA) conducted alongside a significant scale-up will be required to provide a realistic evaluation of this process, as laboratory-scale experiments are intrinsically inefficient. This would provide a more realistic assessment of the overall process compared to lab-scale reactions which are inevitably less efficient.

## Conclusions

We have reported four zinc(ii) catalysts bearing sterically bulky imino-indole ligands that were applied to the selective chemical recycling of three commodity polymers. The catalysts were initially assessed through PLA and BPA-PC methanolysis in THF to benchmark catalytic activity against previously reported systems and to compare structural relationships. Zn(3)_2_ was shown to be the most effective catalyst, and this could be a result of the secondary amine group interacting with the polymer chain. PLA depolymerization to lactide was also demonstrated using a reactive sublimation system with GEO. At equivalent conditions, Zn(2)_2_ was highly competitive with Sn(Oct)_2_ and was similarly active at low catalyst loadings. Circularity of this reaction was demonstrated through repolymerisation of the produced lactide.

The methanolysis of PLA and BPA-PC in neat methanol showed reasonably high activity and important differences in activity between the two polymers at reduced temperatures. Selectivity between PLA and BPA-PC was further optimized to give conditions of 70 °C for two hours with 4 wt% catalyst loading; the BPA-PC remained untouched under these conditions, while PLA was fully converted. PET glycolysis proceeded to high conversion in 60–120 minutes depending on the catalyst used. These results allowed for a demonstration of three-component chemical recycling with a selectivity to target product of >99% during each reaction step, potentially reducing the need for pre-sorting and minimizing chemical separation of product mixtures. This is a significant advancement over the selectivity demonstrated by Zn(ii) pyrrole catalysts (Fig. S10).^24^ The reaction was scaled up to 15 g total polymer, resulting in 17.3 g of total product, either as recrystallised solid or as a concentrated solution in methanol. Remaining methanol from PLA methanolysis was collected and successfully used for BPA-PC methanolysis. Overall, this work represents an exceptionally selective process for the sequential chemical recycling of three common polymers, which could be readily adapted to work with a range of different polymeric materials. Future work will seek to develop the reusability of zinc-systems and gain a deeper understanding of the active catalytic species.

## Author contributions

Jack A. Stewart (conceptualization, validation, investigation, writing – original draft, writing – review & editing). Matthew J. Cullen (investigation, writing – review & editing). Matthew G. Davidson (supervision, resources, funding acquisition, writing – review & editing). Matthew D. Jones (conceptualisation, resources, formal analysis, supervision, funding acquisition, project administration, writing – review & editing).

## Conflicts of interest

There are no conflicts to declare.

## Supplementary Material

GC-028-D6GC03650C-s001

GC-028-D6GC03650C-s002

## Data Availability

All data is available in the supplementary information (SI). Supplementary information is available. See DOI: https://doi.org/10.1039/d6gc03650c. CCDC 2556631 and 2556632 contain the supplementary crystallographic data for this paper.^53*a*,*b*^
