# Supplementary material for: The selective and sequential chemical recycling of commodity polymers with imino-indole zinc(ii) catalysts
Source: Green Chem. 2026 Aug 13;28(35):14320–30. doi: 10.1039/d6gc03650c (PMC13573571; doi:10.1039/d6gc03650c)
Supplement: Supplementary file 1 [file GC-028-D6GC03650C-s001.pdf]

Supplementary Information (SI) for Green Chemistry.
This journal is © The Royal Society of Chemistry 2026

The Selective and Sequential Chemical Recycling of Commodity Polymers with Imino-Indole Zinc (II) Catalysts

Jack A. Stewart,[a] Matthew J. Cullen,[a] Matthew G. Davidson [a] and Matthew D. Jones* [a]

a Department of Chemistry, University of Bath, Claverton Down, Bath, United Kingdom, BA2 7AY. E-mail: mj205@bath.ac.uk

Contents

1. Synthesis and Characterisation 2
   1.1 Synthesis of imino-indole ligands 2
   1.2 Synthesis of imino-indole Zn(II) complexes 8
   1.3 X-ray crystallography 17
2. Solution alcoholysis of PLA and BPA-PC 18
   2.1 PLA methanolysis – conversion, selectivity and yield 18
   2.2 PLA methanolysis – results and kinetics 19
   2.3 BPA-PC methanolysis in THF 20
3. Co-solvent-free alcoholysis of polymers 21
   3.1 PLA alcoholysis in neat alcohol 21
   3.2 BPA-PC alcoholysis in neat methanol 22
   3.3 PET glycolysis 23
4. Chemical recycling to monomer 23
5. Chemical recycling of mixed plastics 24
   5.1 PLA/BPA-PC selectivity optimisation 24
   5.2 Three component chemical recycling 25
6. Green metrics calculations 26
7. Crystallography data 27

1. Synthesis and Characterisation

1.1 Synthesis of imino-indole ligands

1H

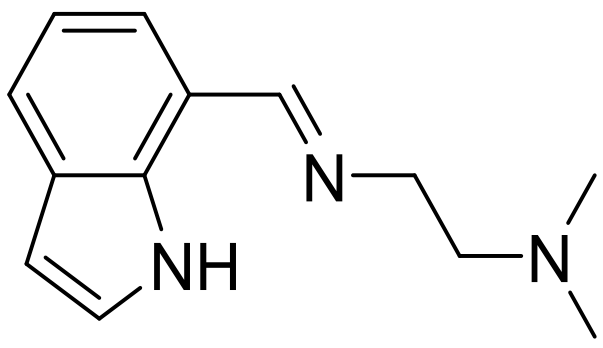

7-Formyl indole (1.452 g, 1 equiv, 10 mmol) and N,N-dimethyl ethylenediamine (1.09 mL, 10 mmol) were added to a round bottomed flask with MeOH (30 mL). The reaction was stirred at room temperature for 24 h and the solution went from colourless to yellow. The solvent was removed *in vacuo* giving a yellow oil as the product, **1**H (1.51 g, 70%).

^{1}H NMR (500 MHz, $CDCl_3$) δ 8.53 (s, 1H, **H**C=N), 7.74 (d, *J* = 7.9 Hz, 1H, Ar**H**), 7.32 (dd, *J* = 3.2, 2.2 Hz, 2H, Ar**H**), 7.29 (dd, *J* = 7.2, 1.0 Hz, 2H, Ar**H**), 7.16 (dd, *J* = 7.8, 7.2 Hz, 2H, Ar**H**), 6.59 (dd, *J* = 3.2, 2.3 Hz, 2H, Ar**H**), 3.85 – 3.79 (m, 2H, C**H**$_2$), 2.73 – 2.68 (m, 2H, C**H**$_2$), 2.35 (s, 6H, C**H**$_3$).

^{13}C{^{1}H} NMR (126 MHz, $CDCl_3$) δ 163.0 (**C**=N), 134.0 (Ar), 128.3 (Ar), 125.2 (Ar), 124.9 (Ar), 123.5 (Ar), 119.4 (Ar), 119.1 (Ar), 102.1 (Ar), 60.7 (**C**H$_2$), 60.0 (**C**H$_2$), 46.0 (**C**H$_3$).

HRMS (ESI^{+}) *m/z*: $[M+H]^+$ calc. for $C_{13}H_{18}N_3$ 216.1501, found 216.1506.

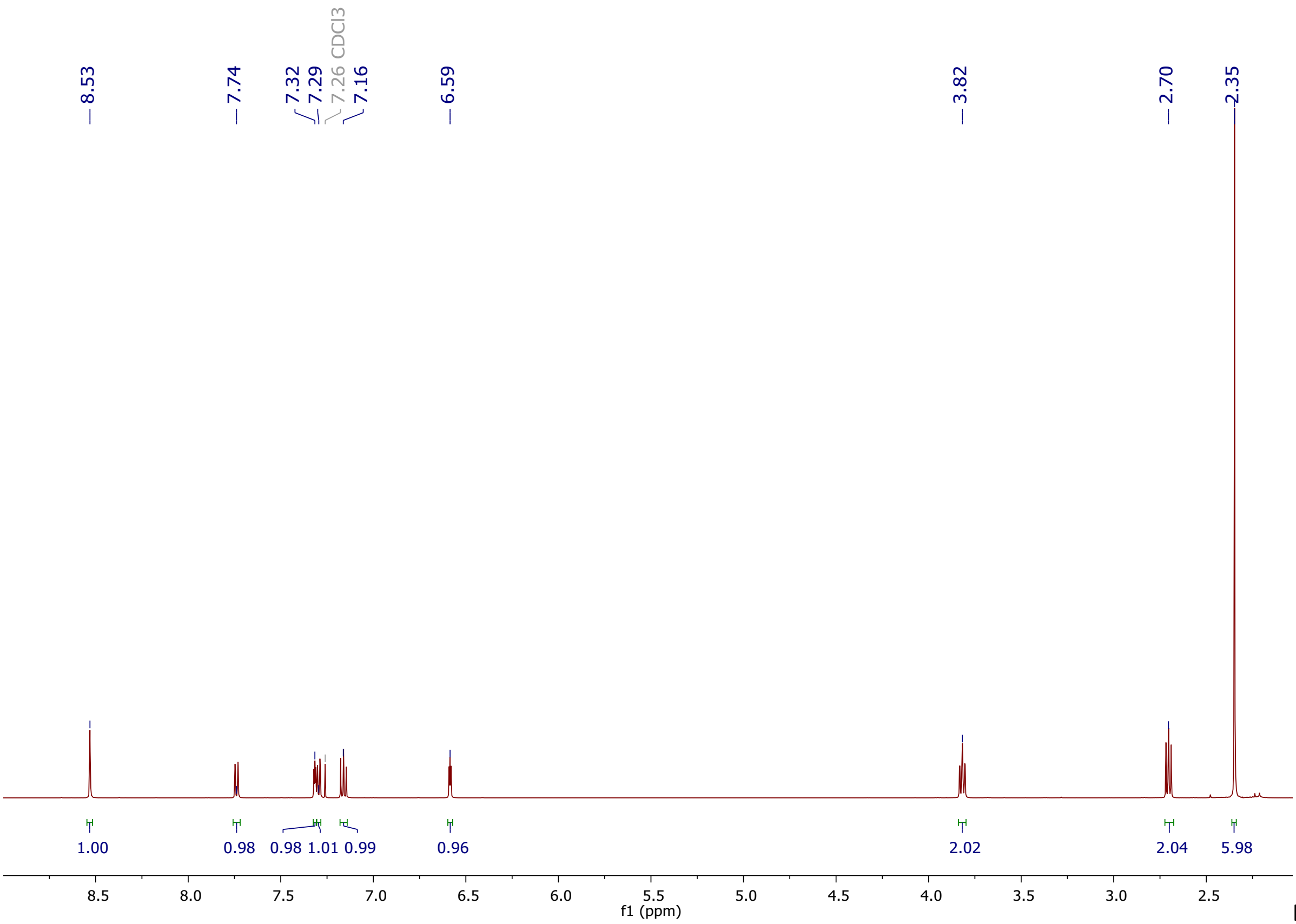

F

igure S1 ^{1}H NMR spectrum (500 MHz, $CDCl_3$) of **1**H.

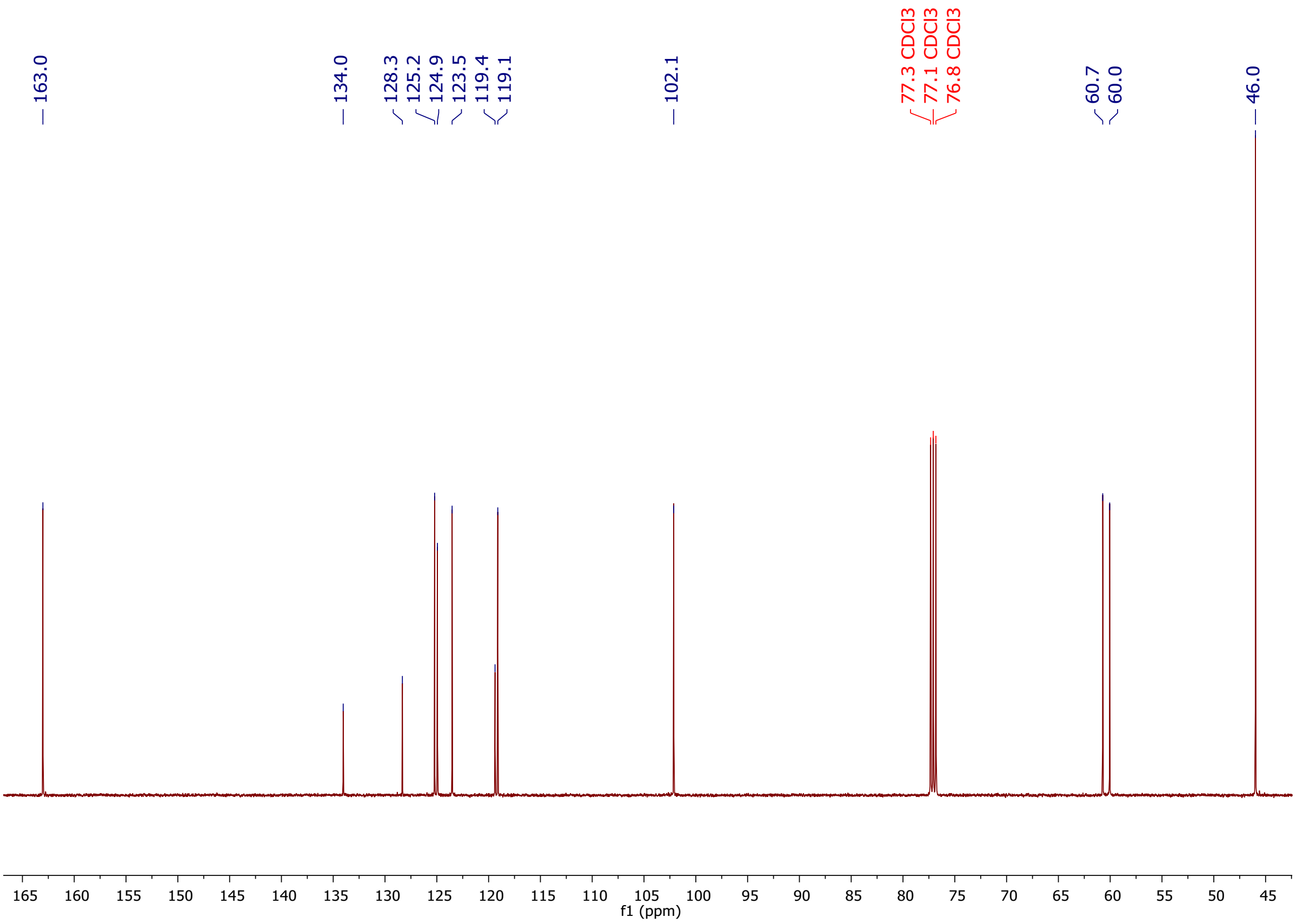

Figure S2 $^{13}C\{^1H\}$ NMR spectrum (126 MHz, $CDCl_3$) of **1**H.

2H

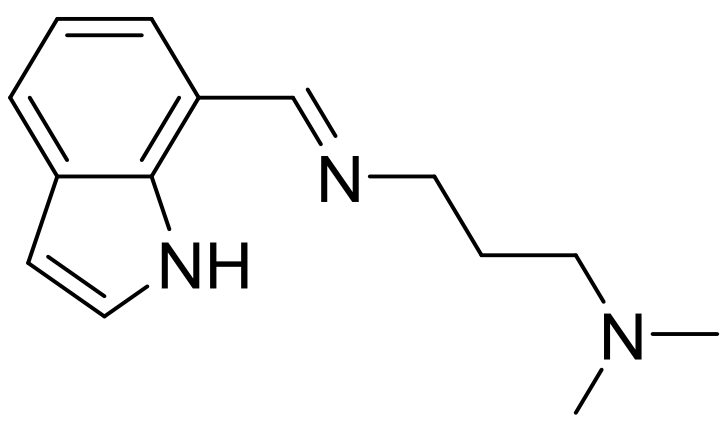

7-Formyl indole (1.452 g, 1 equiv, 10 mmol) and 3-(dimethylamino)-1-propylamine (1.25 mL, 10 mmol) were added to a round bottomed flask with MeOH (30 mL). The reaction was stirred at room temperature for 24 h and the solution went from colourless to yellow. The solvent was removed *in vacuo* giving a yellow oil as the product, **2**H (1.97 g, 86%).

^1H NMR (400 MHz, $CDCl_3$) δ 8.51 (t, *J* = 1.3 Hz, 1H, **H**C=N), 7.73 (dt, *J* = 7.9, 0.7 Hz, 1H, Ar**H**), 7.32 (dd, *J* = 3.2, 2.1 Hz, 1H, Ar**H**), 7.29 (dd, *J* = 7.3, 1.1 Hz, 1H, Ar**H**), 7.16 (dd, *J* = 7.9, 7.2 Hz, 1H, Ar**H**), 6.59 (dd, *J* = 3.1, 2.3 Hz, 1H, Ar**H**), 3.72 (td, *J* = 6.8, 1.4 Hz, 2H, C$\mathbf{H_2}$), 2.49 – 2.35 (m, 2H, C$\mathbf{H_2}$), 2.26 (s, 6H, C$\mathbf{H_3}$), 2.08 – 1.83 (m, 2H, C$\mathbf{H_2}$).

$^{13}C\{^1H\}$ NMR (126 MHz, $CDCl_3$) δ 162.3 (**C**=N), 134.1 (Ar), 128.3 (Ar), 125.0 (Ar), 125.0 (Ar), 123.4 (Ar), 119.5 (Ar), 119.1 (Ar), 102.1 (Ar), 59.7 (**C**H_2), 57.8 (**C**H_2), 45.6 (**C**H_3), 29.5 (**C**H_2).

HRMS (ESI^+) *m/z*: $[M+H]^+$ calc. for $C_{14}H_{20}N_3$ 230.1657, found 230.1654.

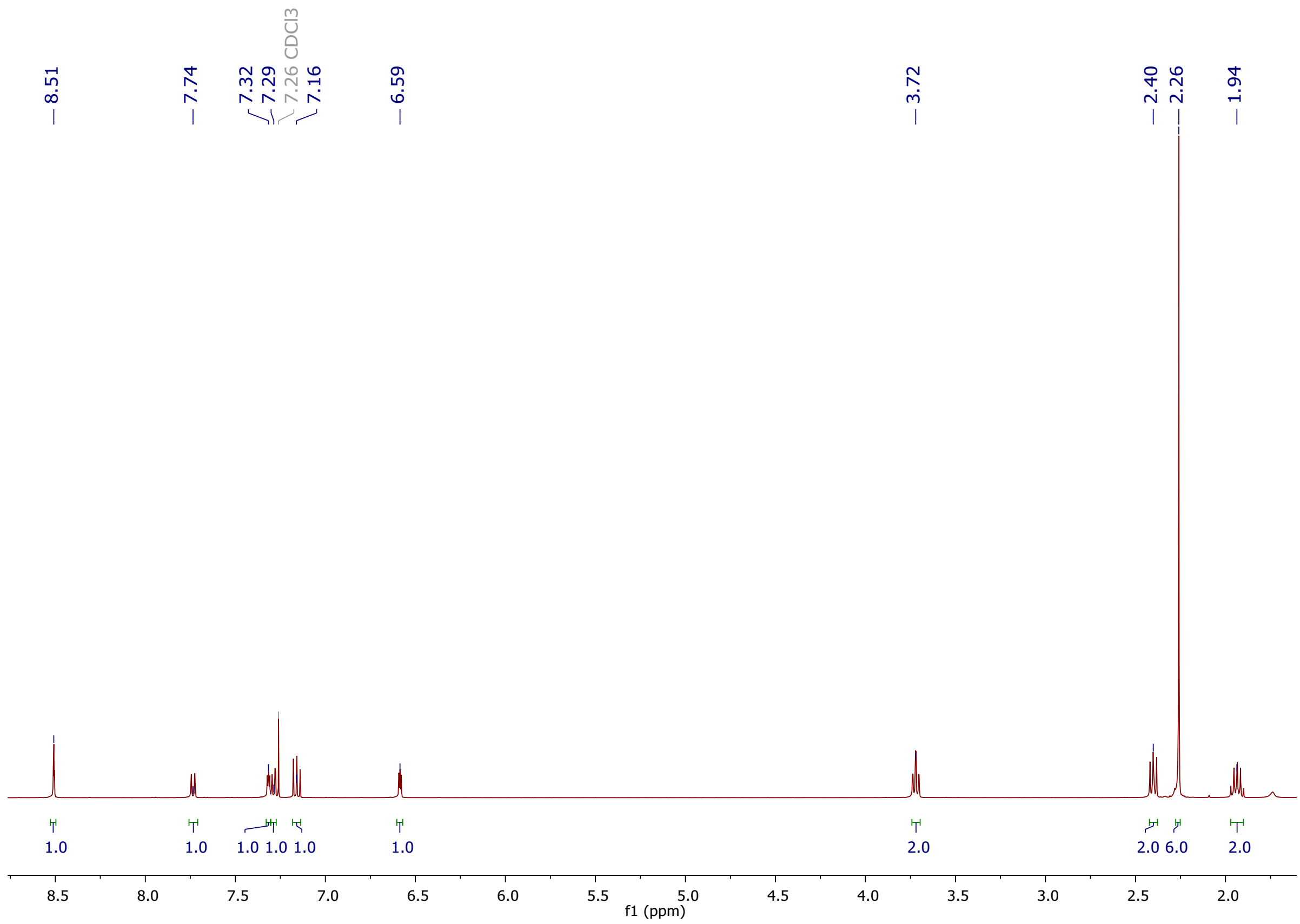

Figure S3 ^{1}H NMR spectrum (500 MHz, $CDCl_3$) of **2**H.

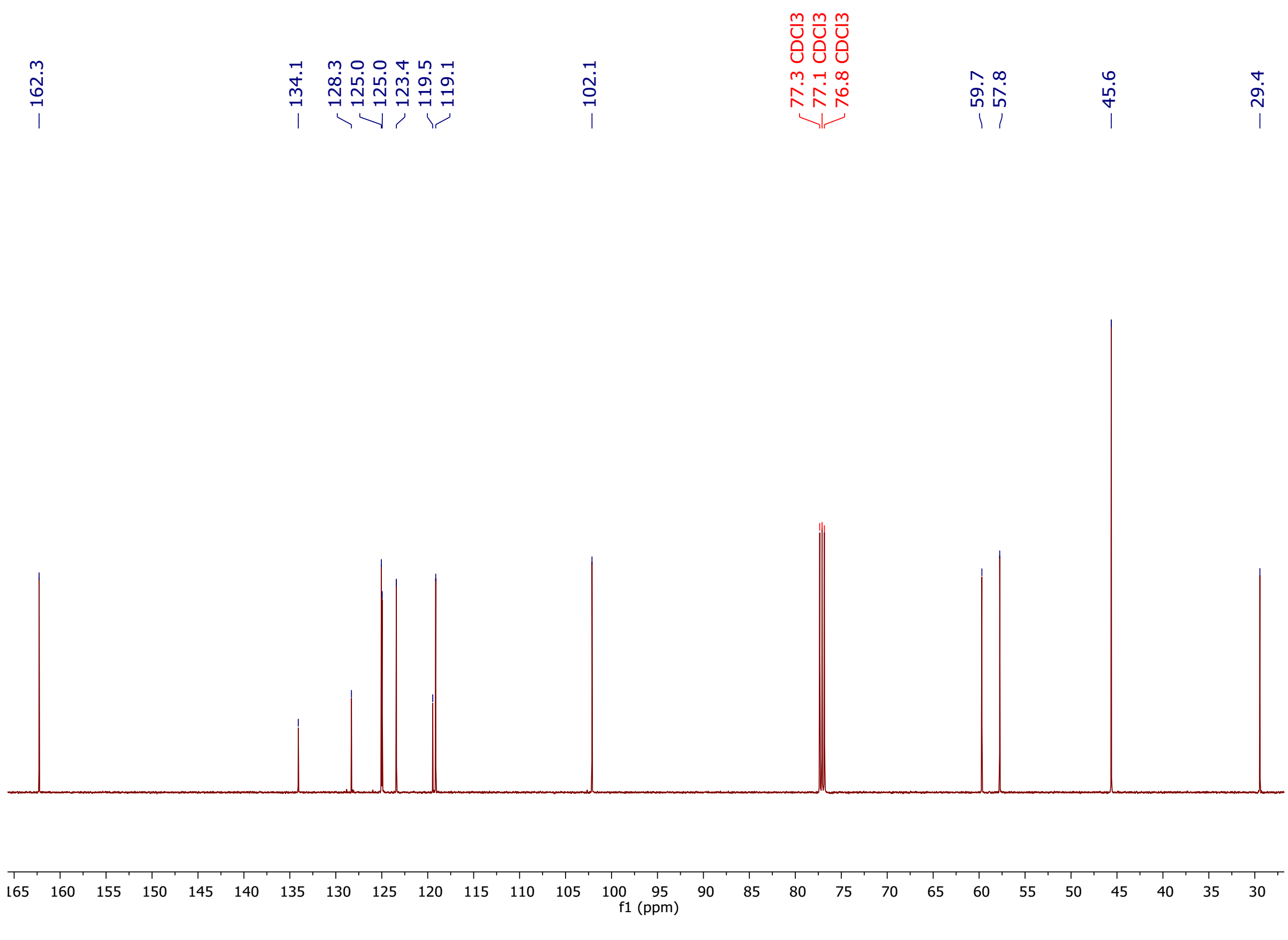

Figure S4 $^{13}C\{^{1}H\}$ NMR spectrum (126 MHz, $CDCl_3$) of **2**H.

3H

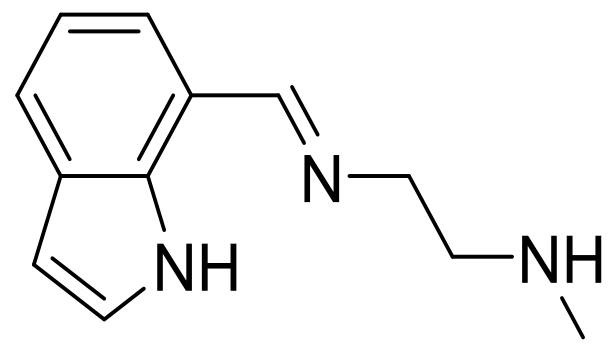

3H*

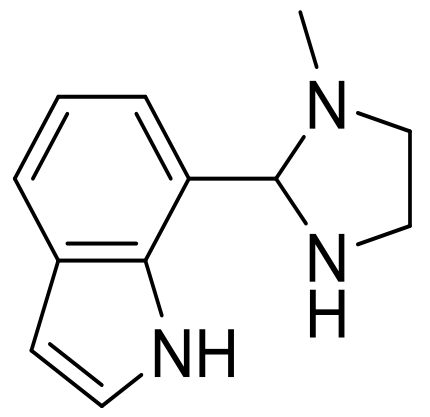

7-Formyl indole (1.452 g, 1 equiv, 10 mmol) and N-methylethylenediamine (0.87 mL, 10 mmol) were added to a round bottomed flask with MeOH (30 mL). The reaction was stirred at room temperature for 24 h and the solution went from colourless to yellow. The solvent was removed *in vacuo* giving a yellow oil as the product, **3**H (1.45 g, 72%). A smaller series of peaks can be seen which correspond to a cyclised byproduct, **3**H*.

3H ^{1}H NMR (400 MHz, $CDCl_3$) δ 8.54 (t, *J* = 1.3 Hz, 1H, **H**C=N), 7.74 (dt, *J* = 7.8, 0.9 Hz, 1H, Ar**H**), 7.51 – 7.29 (m, 2H, Ar**H**), 7.16 (dd, *J* = 7.9, 7.2 Hz, 1H, Ar**H**), 6.59 (dd, *J* = 3.2, 2.3 Hz, 1H, Ar**H**), 3.84 (ddd, *J* = 7.1, 4.8, 1.3 Hz, 2H, C**H**$_2$), 3.25 – 2.80 (m, 2H, C**H**$_2$), 2.49 (s, 3H, C**H**$_3$).

3H* ^{1}H NMR (400 MHz, $CDCl_3$) δ 7.62 (dt, *J* = 7.8, 0.9 Hz, 1H, Ar**H**), 7.21 (dd, *J* = 3.2, 2.3 Hz, 1H, Ar**H**), 7.07 (dd, *J* = 7.9, 7.2 Hz, 1H, Ar**H**), 6.55 (dd, *J* = 3.2, 2.3 Hz, 1H, Ar**H**), 4.24 (s, 1H, C**H**), 3.43 (ddd, *J* = 9.0, 7.6, 2.8 Hz, 2H, C**H**$_2$), 3.31 (ddd, *J* = 10.3, 8.7, 7.5 Hz, 2H, C**H**$_2$), 3.18 (ddd, *J* = 10.2, 8.5, 2.8 Hz, 2H, C**H**$_2$), 2.55 (d, *J* = 8.8 Hz, 2H, C**H**$_2$), 2.31 (s, 3H, C**H**$_3$).

3H ^{13}C NMR (126 MHz, $CDCl_3$) δ 163.6 (**C**=N), 134.0 (Ar), 128.4 (Ar), 125.4 (Ar), 125.0 (Ar), 123.7 (Ar), 119.3 (Ar), 119.2 (Ar), 102.3 (Ar), 61.5 (**C**H$_2$), 52.5 (**C**H$_2$), 36.5 (**C**H$_3$).

3H* ^{13}C NMR (126 MHz, $CDCl_3$) δ 128.9 (Ar), 124.0 (Ar), 122.3 (Ar), 121.7 (Ar), 120.8 (Ar), 119.4 (Ar), 102.2 (Ar), 85.1 (**C**H), 55.5 (**C**H$_2$), 44.6 (**C**H$_2$), 38.8 (**C**H$_3$).

HRMS (ESI$^+$) *m/z*: $[M+H]^+$ calc. for $C_{12}H_{15}N_3$ 201.1266, found 202.1339.

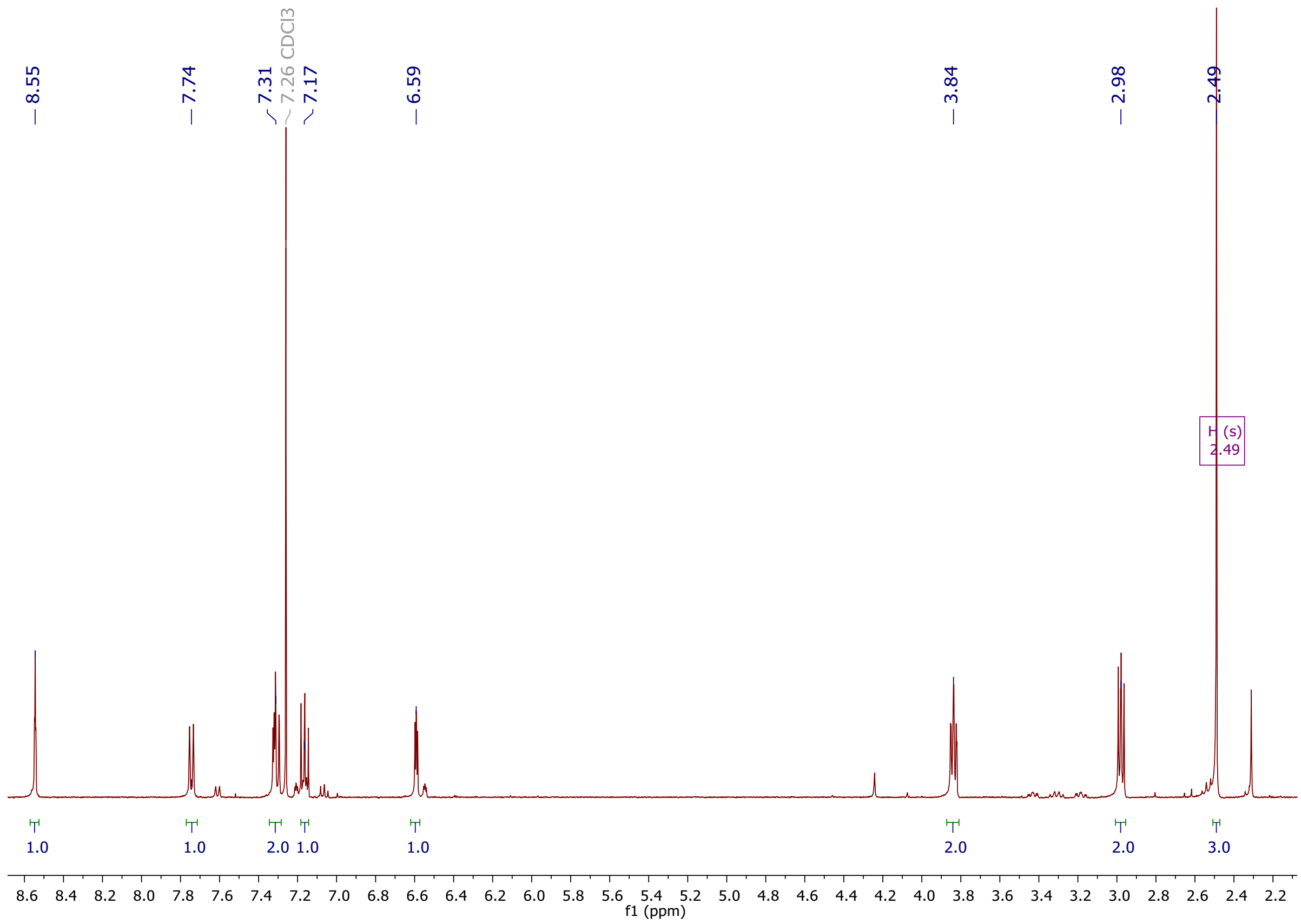

Figure S5 ^{1}H NMR spectrum (500 MHz, $CDCl_3$) of **3**H and **3**H*.

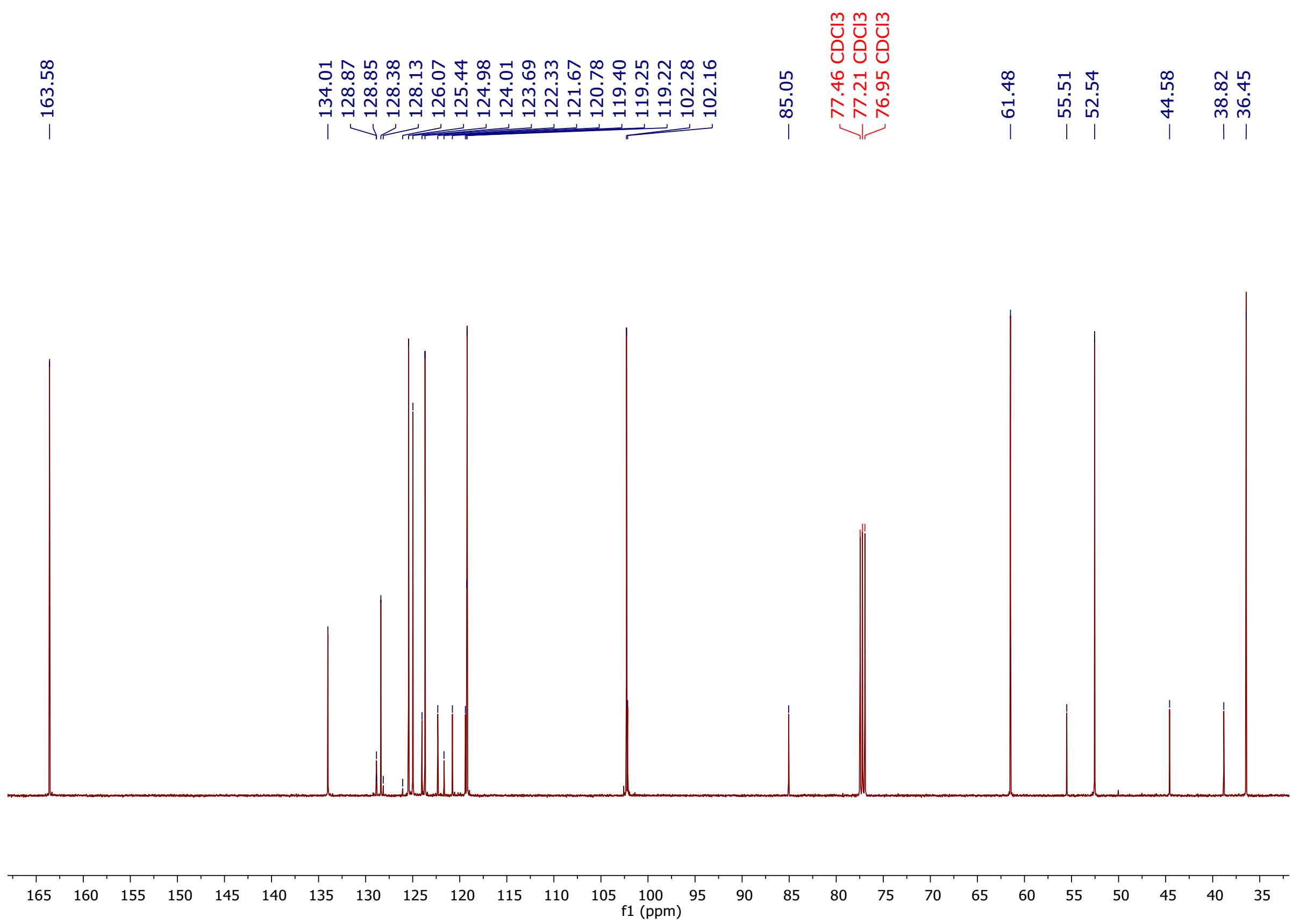

Figure S6 $^{13}C\{^{1}H\}$ NMR spectrum (126 MHz, $CDCl_3$) of **3**H and **3**H*.

4H

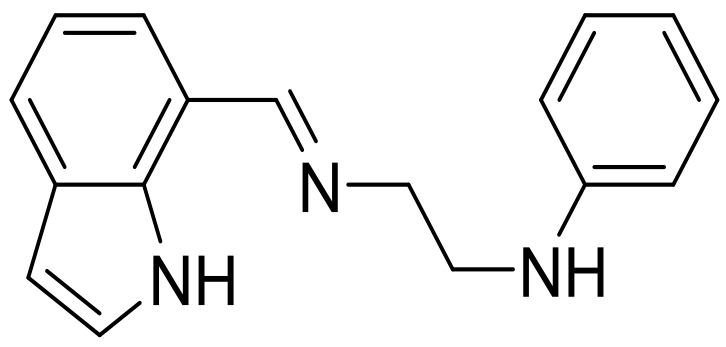

7-Formyl indole (1.452 g, 1 equiv, 10 mmol) and N-phenylethylenediamine (1.31 mL, 10 mmol) were added to a round bottomed flask with MeOH (30 mL). The reaction was stirred at room temperature for 24 h and the solution went from colourless to yellow. The solvent was removed *in vacuo* giving a yellow oil as the product, **4**H (2.24 g, 85%).

^{1}H NMR (500 MHz, $CDCl_3$) δ 10.79 (s, 1H, N**H**), 8.60 (s, 1H, N=C**H**), 7.96 (d, *J* = 7.8 Hz, 1H, Ar**H**), 7.54 – 7.43 (m, 2H, Ar**H**), 7.38 (dt, *J* = 11.1, 7.6 Hz, 3H, Ar**H**), 6.94 (t, *J* = 7.4 Hz, 1H, Ar**H**), 6.83 (d, *J* = 8.0 Hz, 2H, Ar**H**), 6.80 (s, 1H, Ar**H**), 4.00 (t, *J* = 5.9 Hz, 2H, C**H**$_2$), 3.67 (t, *J* = 5.9 Hz, 2H, C**H**$_2$).

^{13}C NMR (126 MHz, $CDCl_3$) δ 193.7 (**C**=N), 164.3 (Ar), 148.2 (Ar), 134.1 (Ar), 129.6 (Ar), 128.6 (Ar), 125.7 (Ar), 125.3 (Ar), 124.1 (Ar), 119.4 (Ar), 119.3 (Ar), 117.8 (Ar), 113.4 (Ar), 102.5 (Ar), 60.6 (**C**H$_2$), 44.8 (**C**H$_2$).

HRMS (ESI$^+$) *m/z*: $[M+H]^+$ calc. for $C_{17}H_{17}N_3$ 263.1422, found 264.1497.

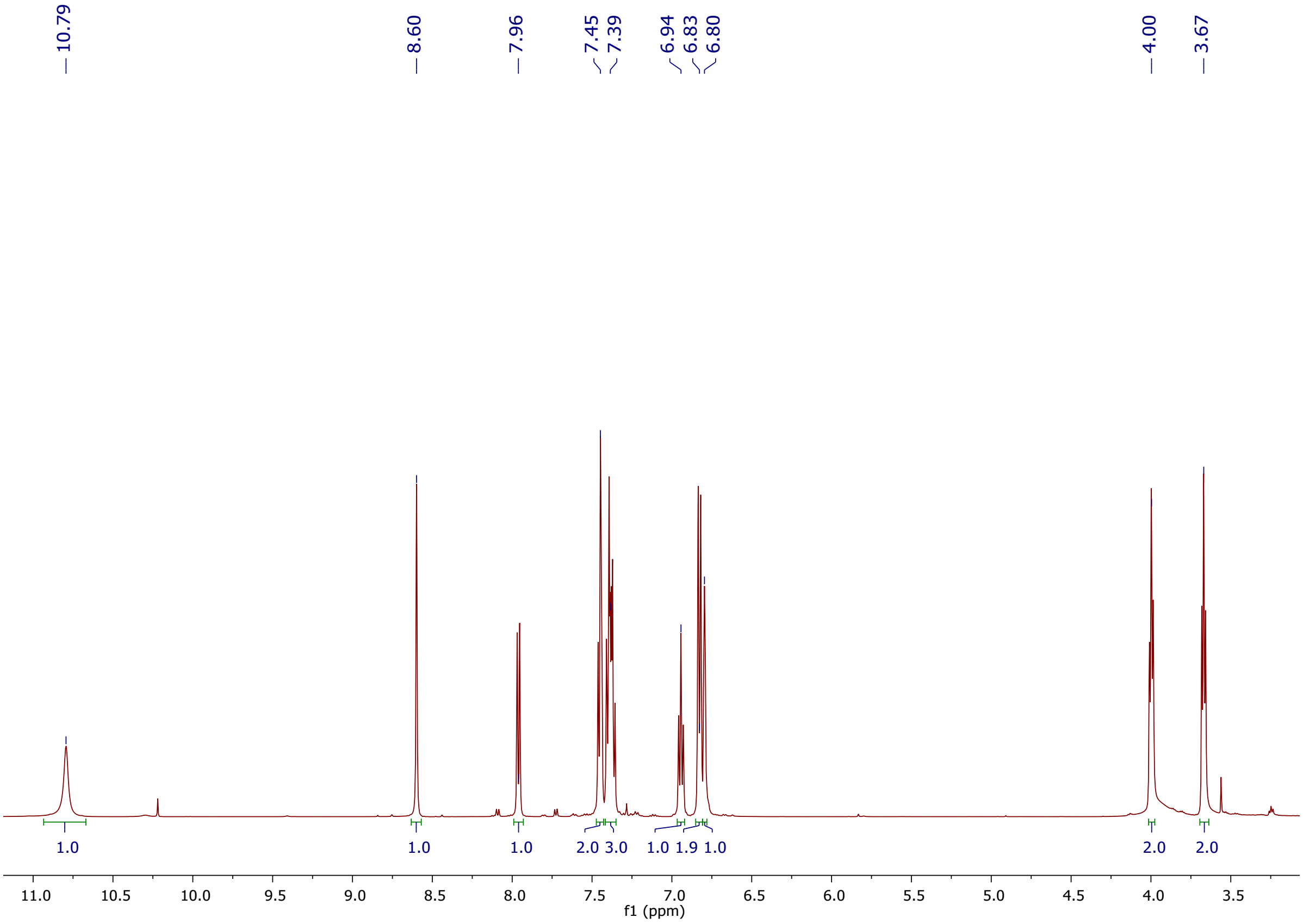

Figure S7 ^{1}H NMR spectrum (500 MHz, $CDCl_3$) of **4**H.

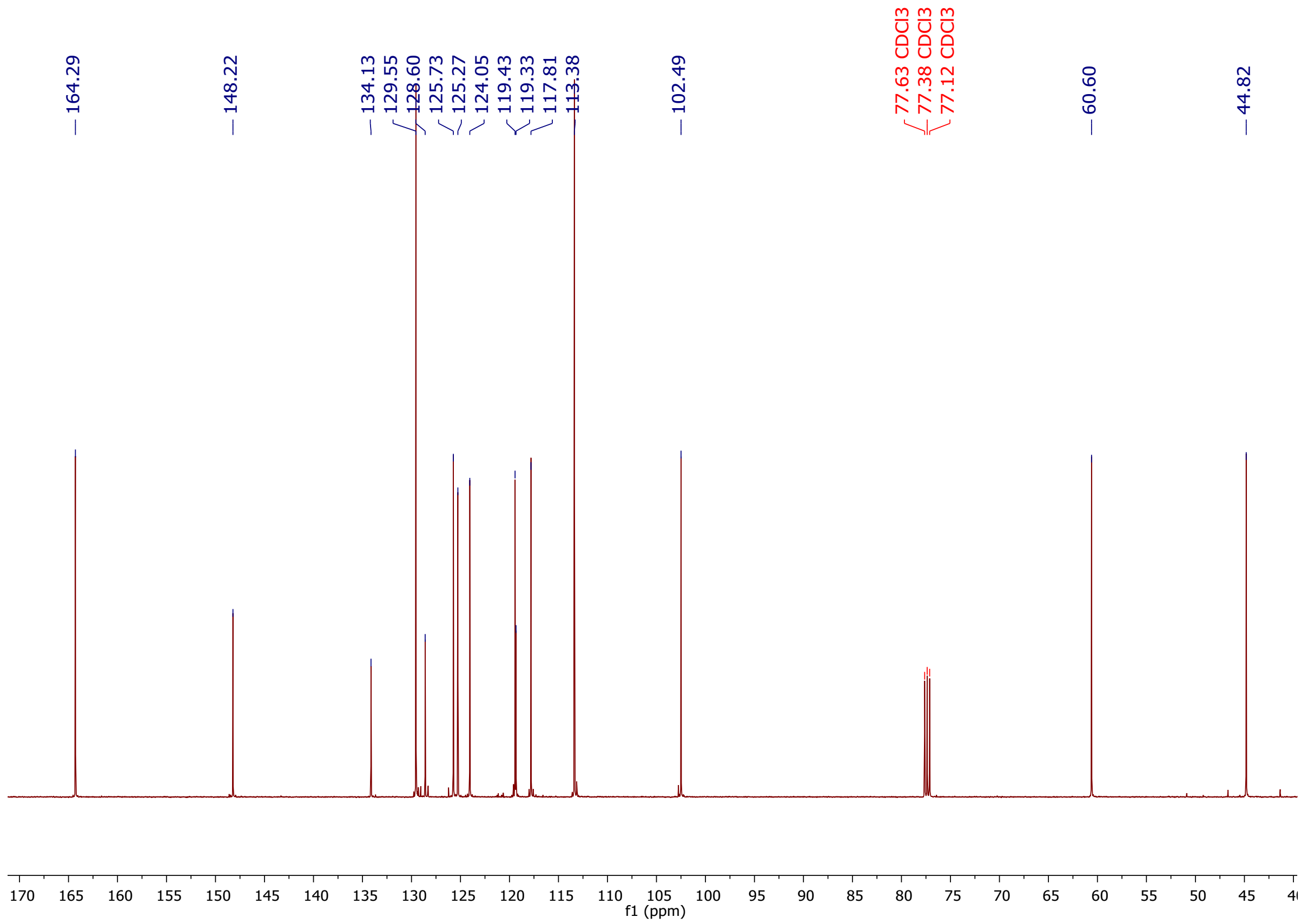

Figure S8 $^{1}H\{^{13}C\}$ NMR spectrum (126 MHz, $CDCl_3$) of **4**H

1.2 Synthesis of imino-indole Zn(II) complexes

Zn(**1**)$_2$

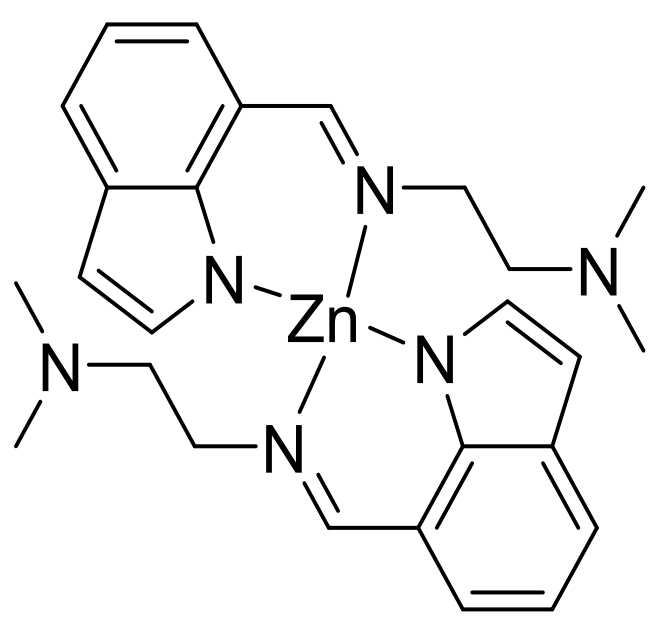

Ligand **1**H (0.861 g, 2 equiv, 4 mmol) was dried in vacuo for two hours and dissolved in anhydrous toluene (10 mL) in a Schlenk flask. Zn(Et)$_2$ (2 mL, 1 equiv, 2 mmol) was added dropwise. The solution was stirred overnight before removal of toluene *in vacuo* to give a yellow solid. The crude product was dissolved in hexane with heating and formed yellow crystals of Zn(**1**)$_2$ upon cooling (0.46 g, 47%).

^{1}H NMR (400 MHz, $CDCl_3$) δ 8.50 (s, 1H, **H**C=N), 7.96 (dd, *J* = 7.7, 1.1 Hz, 1H, Ar**H**), 7.46 (d, *J* = 2.6 Hz, 1H, Ar**H**), 7.31 (dd, *J* = 7.3, 1.1 Hz, 1H, Ar**H**), 7.10 (t, *J* = 7.5 Hz, 1H, Ar**H**), 6.70 (d, *J* = 2.6 Hz, 1H, Ar**H**), 3.73 – 3.30 (m, 2H, C**H**$_2$), 2.30 – 2.00 (m, 2H, C**H**$_2$), 1.84 (s, 6H, C**H**$_3$).

^{13}C NMR (126 MHz, $CDCl_3$) δ 170.1 (**C**=N), 141.8 (Ar), 137.0 (Ar), 131.7 (Ar), 128.0 (Ar), 126.9 (Ar), 116.7 (Ar), 116.7 (Ar), 102.1 (Ar), 58.7 (**C**H$_2$), 58.4 (**C**H$_2$), 45.1 (**C**H$_3$).

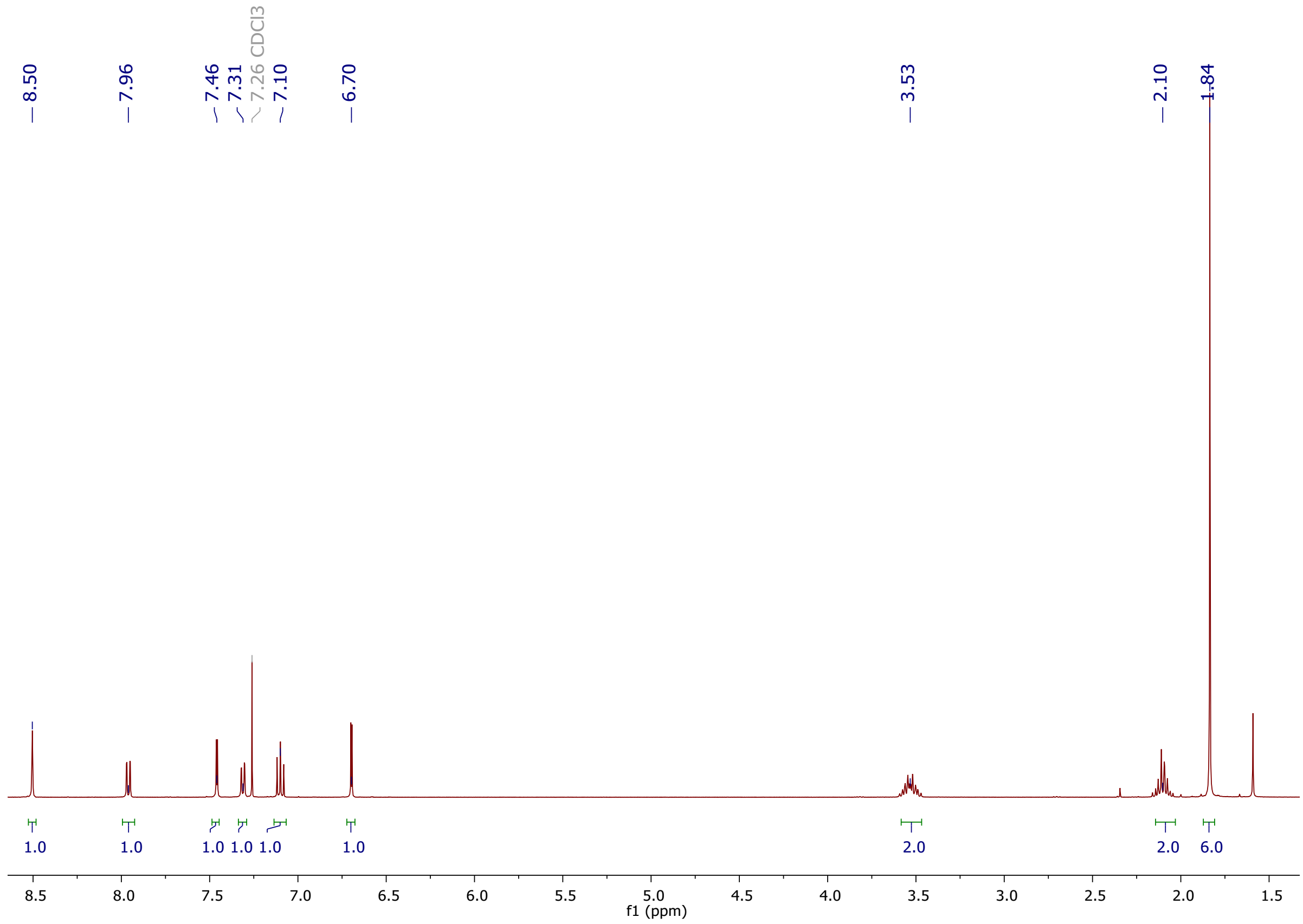

Figure S9 ^{1}H NMR spectrum (400 MHz, $CDCl_3$) of Zn(**1**)$_2$.

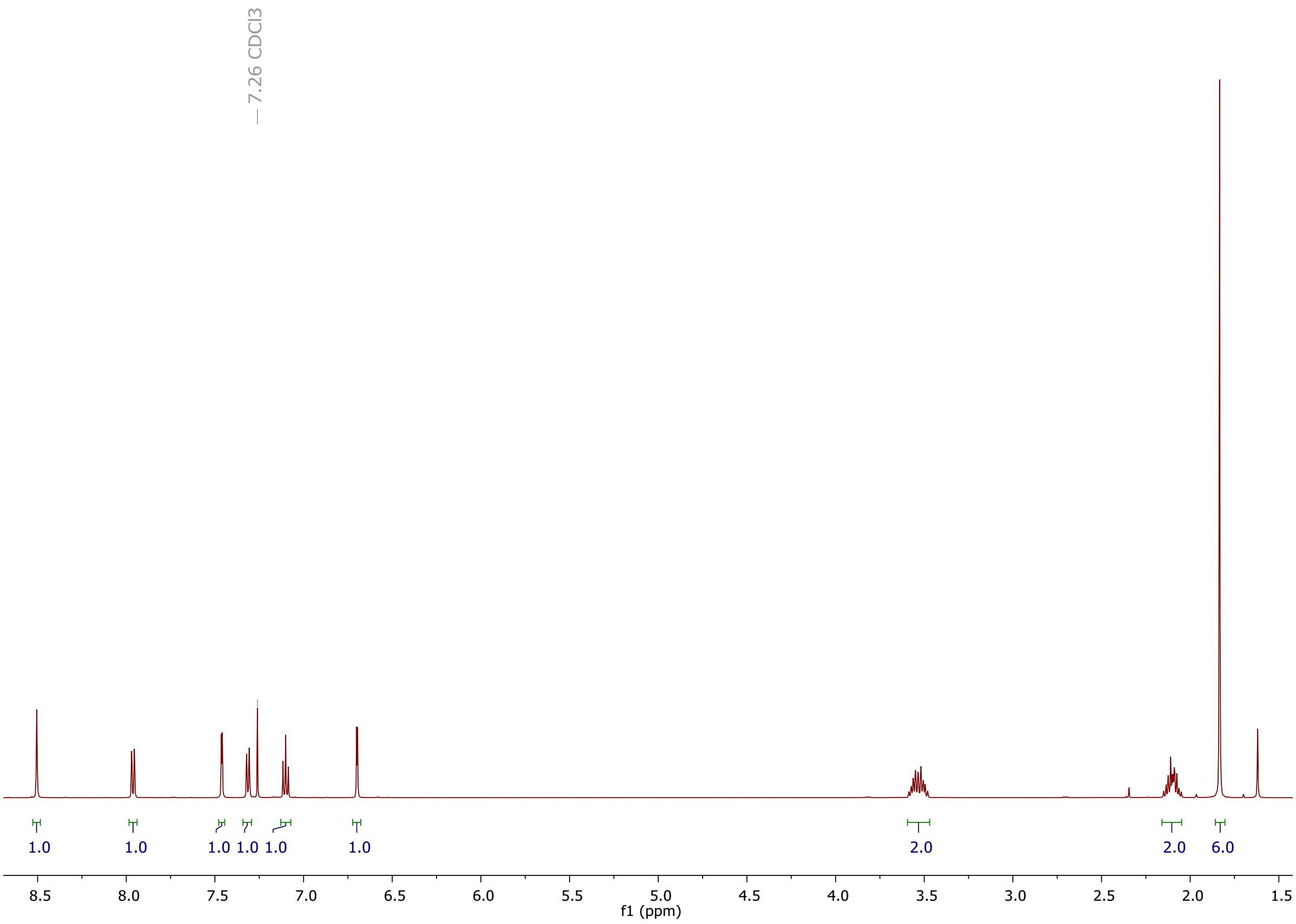

Figure S10 ^{1}H NMR spectrum (400 MHz, $CDCl_3$) of Zn(**1**)$_2$ after one week stored in air.

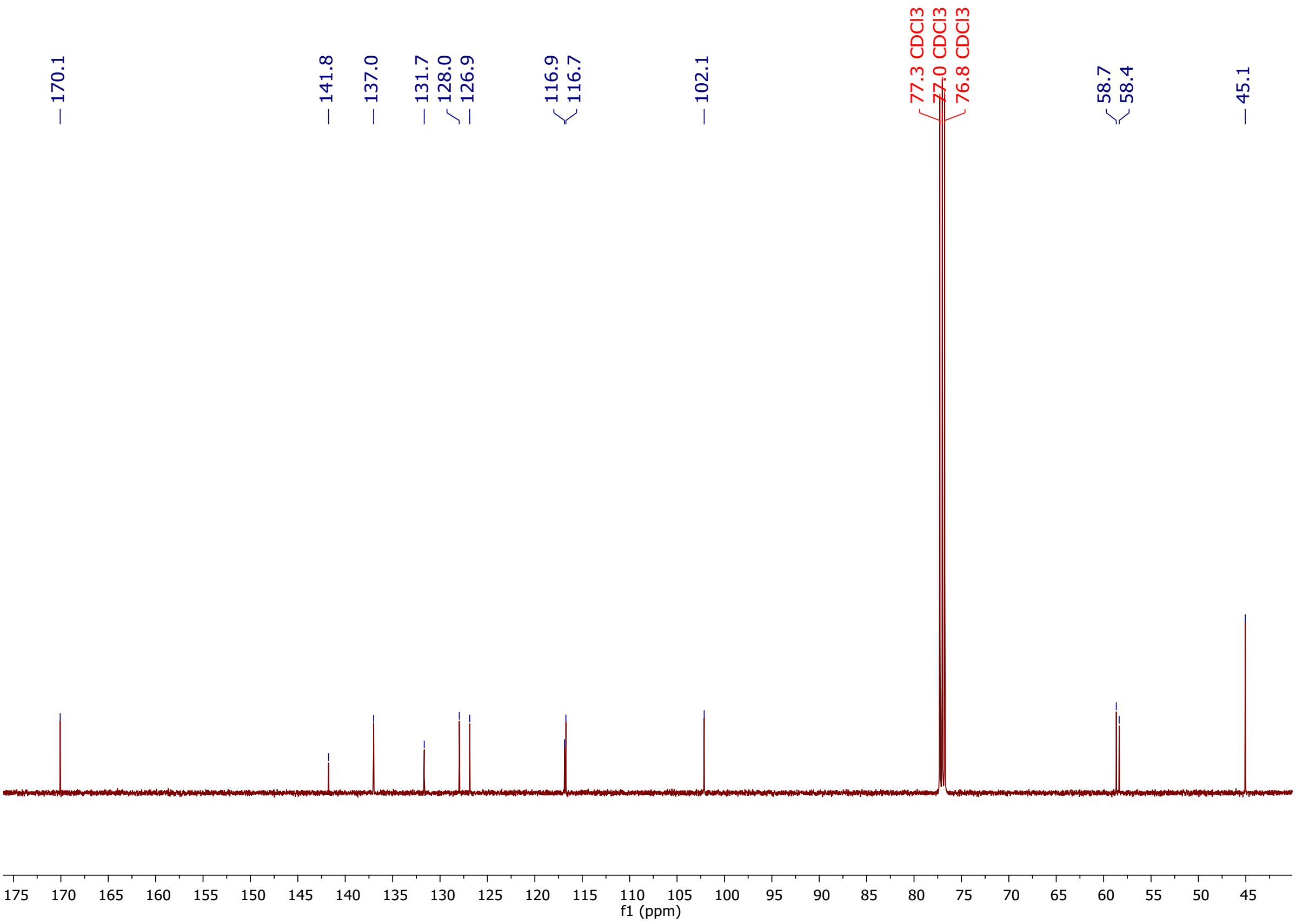

Figure S11 $^{13}C\{^{1}H\}$ NMR spectrum (126 MHz, $CDCl_3$) of Zn(**1**)$_2$.

Zn(**2**)$_2$

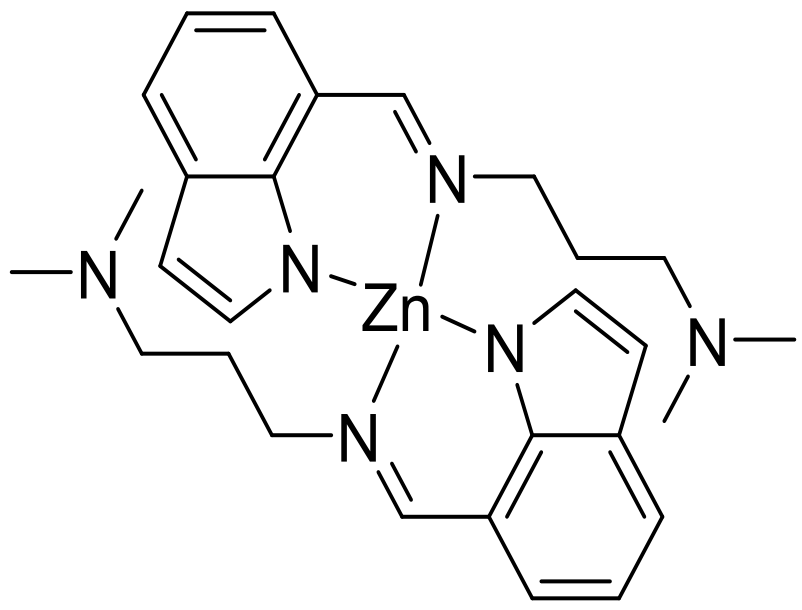

Ligand **2**H (0.459 g, 2 equiv, 2 mmol) was dried in vacuo for two hours and dissolved in anhydrous toluene (10 mL) in a Schlenk flask. Zn(Et)$_2$ (1 mL, 1 equiv, 1 mmol) was added dropwise. The solution was stirred overnight before removal of toluene *in vacuo* to give a yellow solid. The crude product was dissolved in hexane with heating and formed yellow crystals of Zn(**2**)$_2$ upon cooling (0.26 g, 49%).

^{1}H NMR (400 MHz, CDCl$_3$) δ 8.55 (d, *J* = 1.1 Hz, 1H, **H**C=N), 7.97 (dd, *J* = 7.7, 1.1 Hz, 1H, Ar**H**), 7.45 (d, *J* = 2.6 Hz, 1H, Ar**H**), 7.32 (dd, *J* = 7.3, 1.2 Hz, 1H, Ar**H**), 7.11 (t, *J* = 7.5 Hz, 1H, Ar**H**), 6.70 (d, *J* = 2.6 Hz, 2H, Ar**H**), 3.65 – 3.38 (m, 2H, C**H**$_2$), 2.05 (dt, *J* = 12.2, 7.2 Hz, 2H, C**H**$_2$), 1.99 – 1.89 (m, 2H, C**H**$_2$), 1.87 (s, 6H, C**H**$_3$), 1.53 – 1.25 (m, 2H, C**H**$_2$).

^{13}C NMR (101 MHz, CDCl$_3$) δ 168.9 (**C**=N), 140.9 (Ar), 135.9 (Ar), 130.6 (Ar), 126.9 (Ar), 125.9 (Ar), 115.8 (Ar), 115.8 (Ar), 101.2 (Ar), 57.8 (**C**H$_2$), 55.1 (**C**H$_2$), 43.9 (**C**H$_3$), 26.8 (**C**H$_2$).

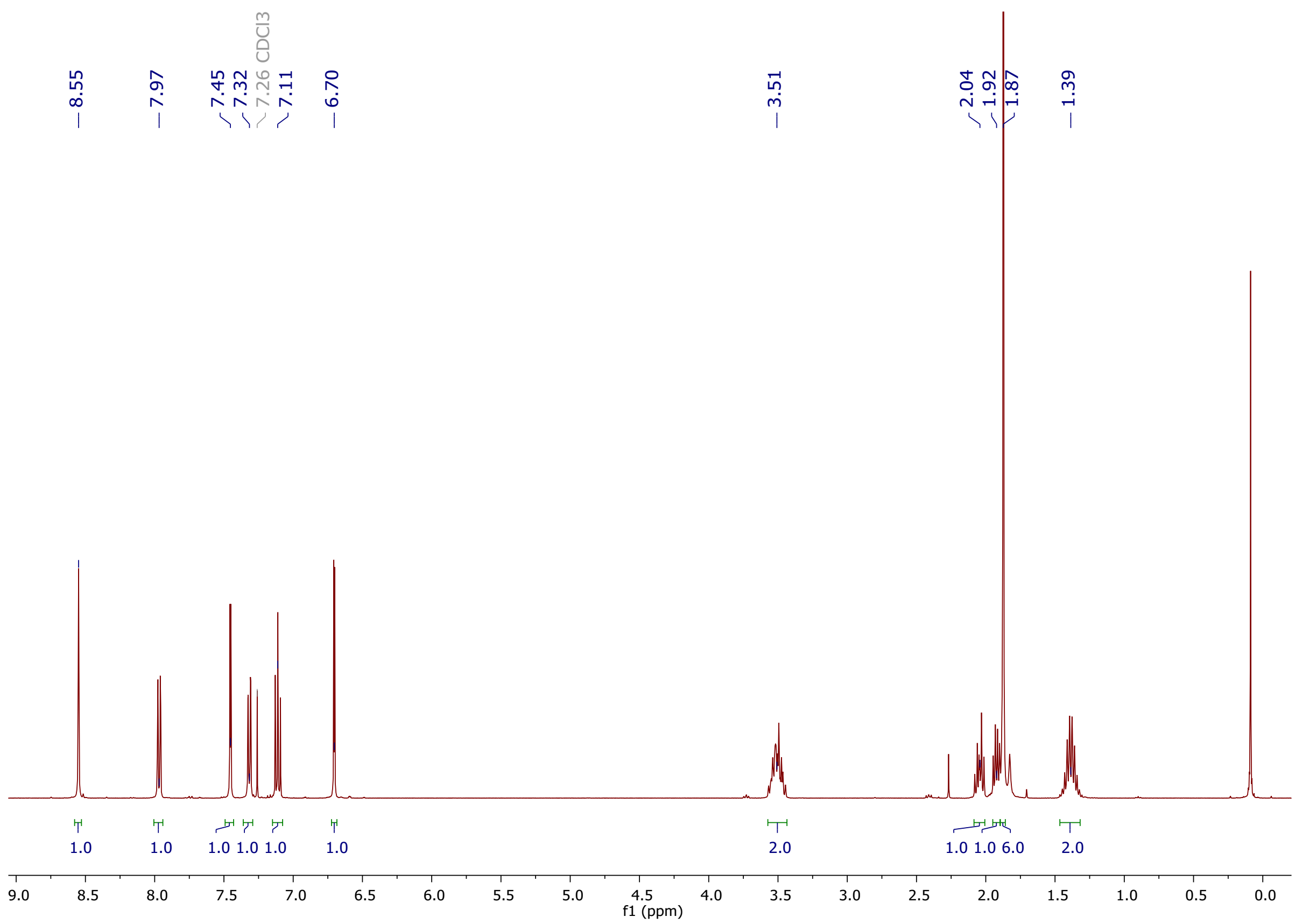

Figure S12 ^{1}H NMR spectrum (400 MHz, CDCl$_3$) of Zn(**2**)$_2$.

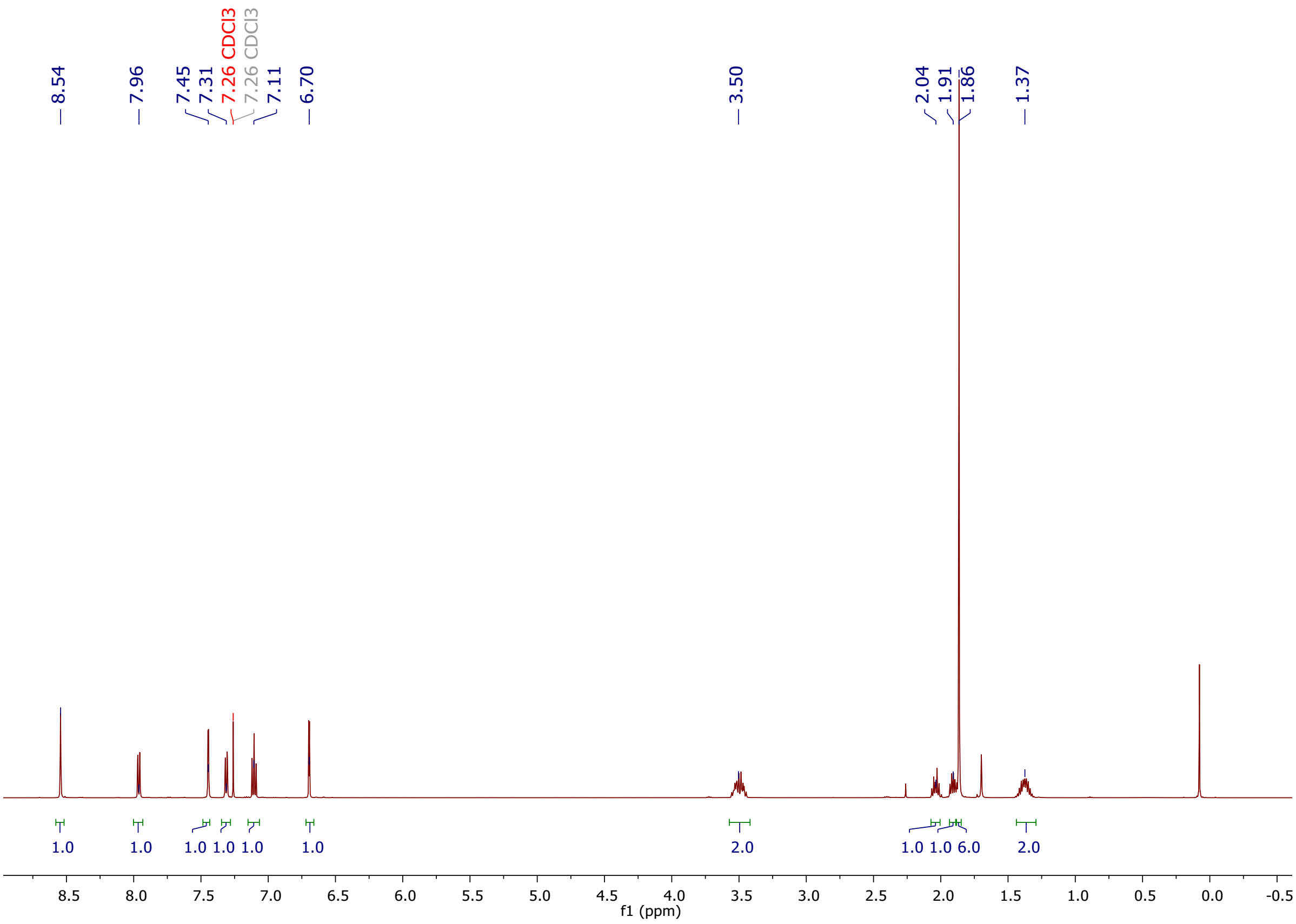

Figure S13 ^{1}H NMR spectrum (500 MHz, $CDCl_3$) of Zn(**2**)$_2$ after one week stored in air.

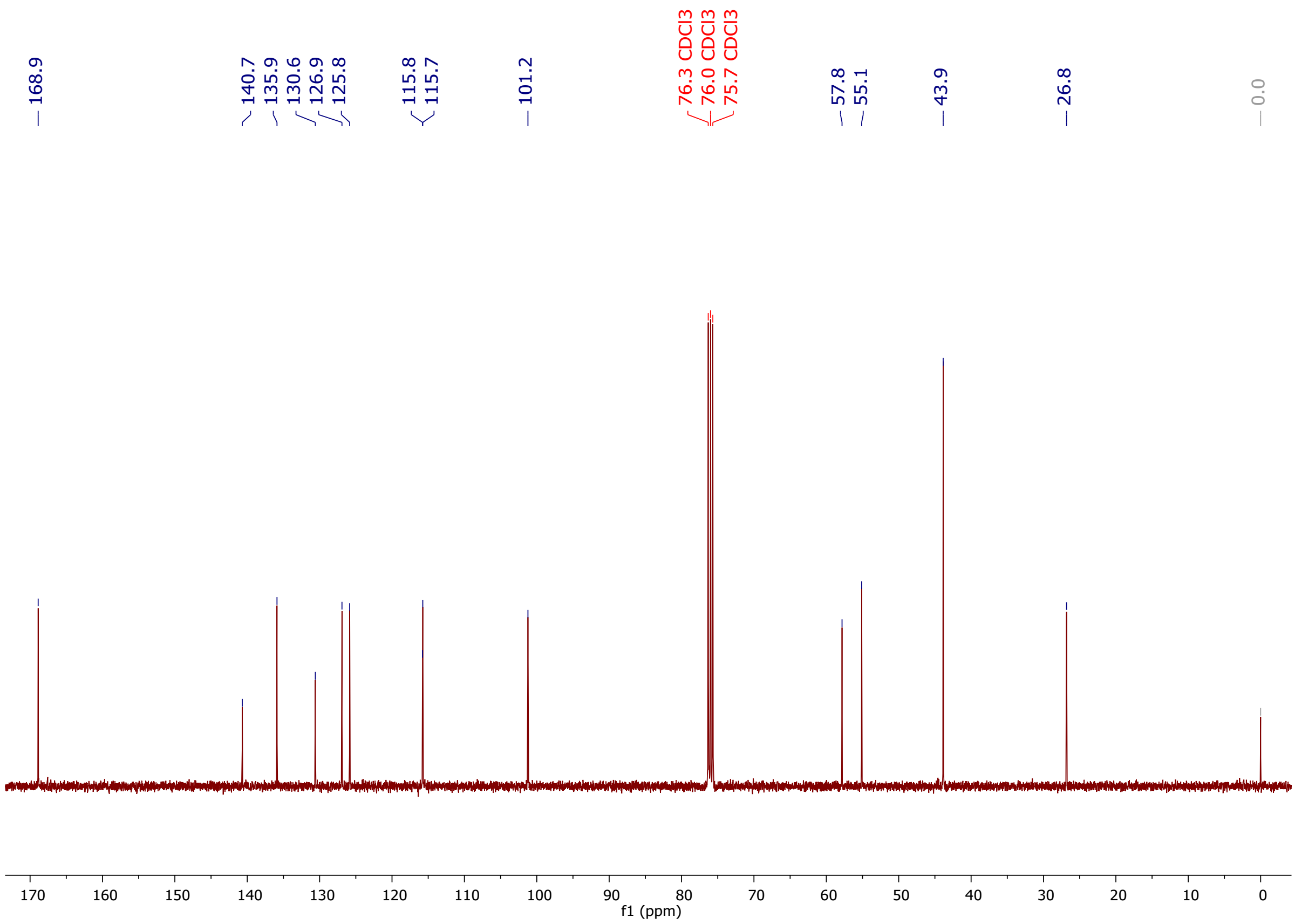

Figure S14 $^{13}C\{^{1}H\}$ NMR spectrum (101 MHz, $CDCl_3$) of Zn(**2**)$_2$.

Zn(**3**)$_2$

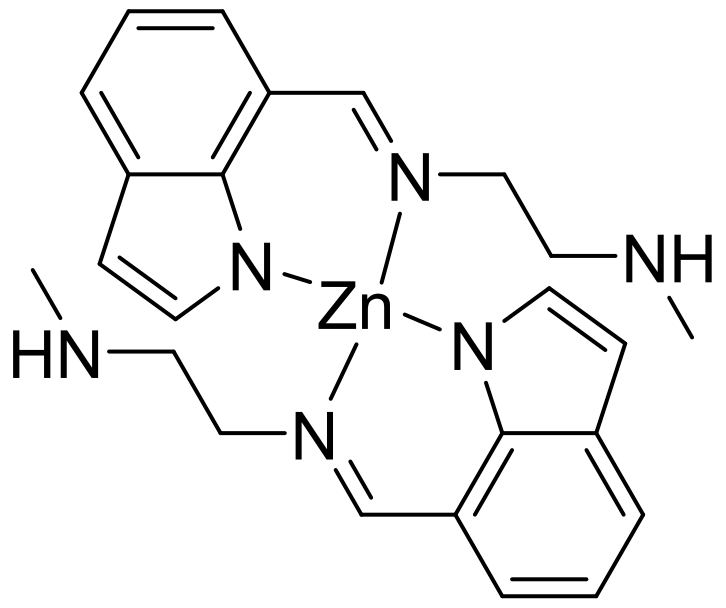

Ligand **3**H (0.403 g, 2 equiv, 2 mmol) was dried in vacuo for two hours and dissolved in anhydrous toluene (10 mL) in a Schlenk flask. Zn(Et)$_2$ (1 mL, 1 equiv, 1 mmol) was added dropwise. The solution was stirred overnight, and hexane was layered (5 mL). Zn(**3**)$_2$ formed at the solvent interface and was collected as a yellow solid (0.056 g, 12%).

^{1}H NMR (500 MHz, $CDCl_3$) δ 8.58 (s, 1H, **H**C=N), 7.96 (d, *J* = 7.7 Hz, 1H, Ar**H**), 7.39 (d, *J* = 2.5 Hz, 1H, Ar**H**), 7.32 (d, *J* = 7.3 Hz, 1H, Ar**H**), 7.10 (t, *J* = 7.5 Hz, 1H, Ar**H**), 6.67 (d, *J* = 2.5 Hz, 1H, Ar**H**), 3.94 – 3.44 (m, 2H, C**H**$_2$), 2.48 (qt, *J* = 12.3, 6.1 Hz, 2H, C**H**$_2$), 2.05 (d, *J* = 4.2 Hz, 3H, C**H**$_3$).

^{13}C NMR (126 MHz, $CDCl_3$) δ 170.0 (**C**=N), 141.8 (Ar), 136.9 (Ar), 131.7 (Ar), 127.9 (Ar), 126.8 (Ar), 116.9 (Ar), 116.6 (Ar), 101.9 (Ar), 60.4 (**C**H$_2$), 50.9 (**C**H$_2$), 35.9 (**C**H$_3$).

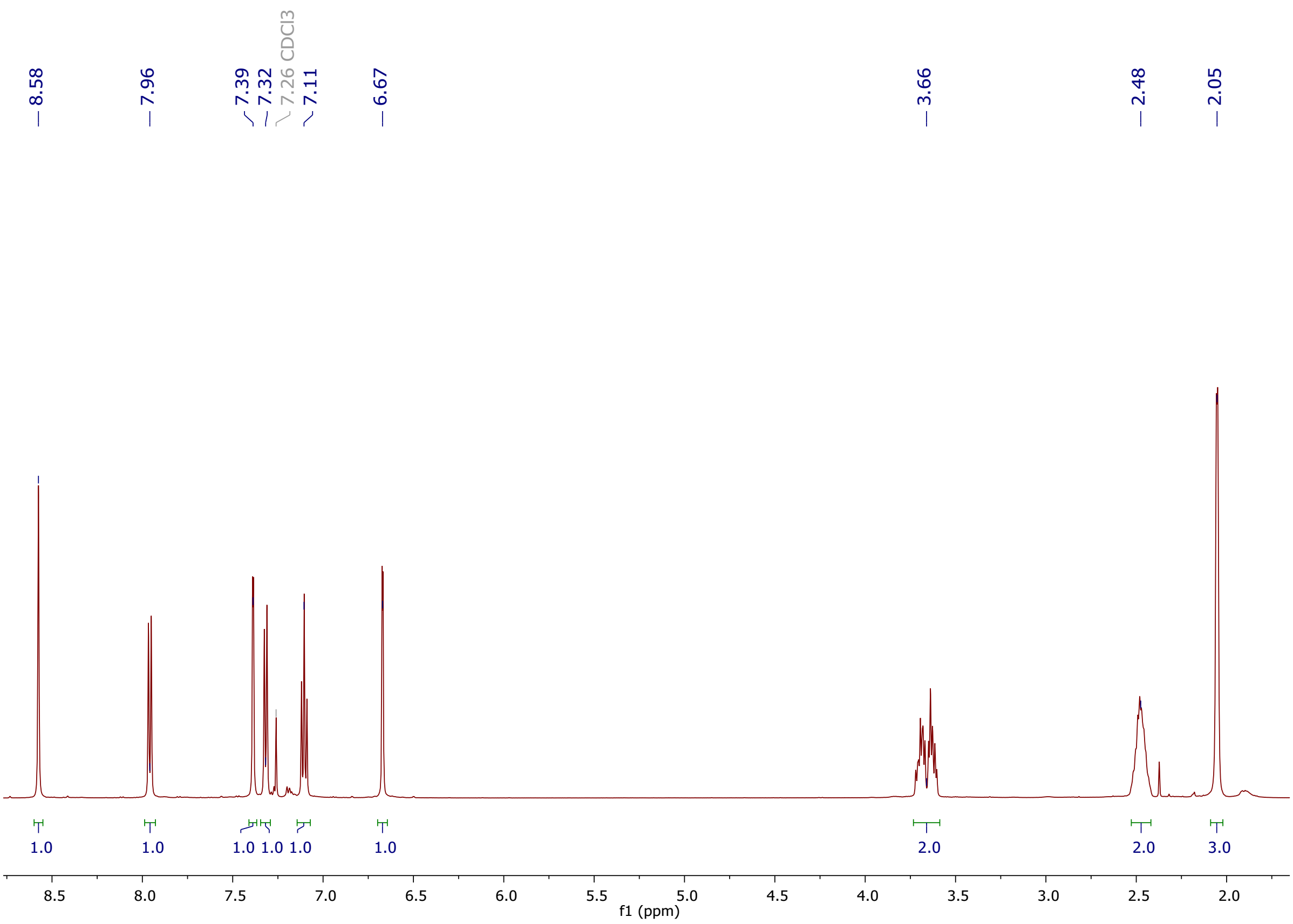

Figure S15 ^{1}H NMR spectrum (500 MHz, $CDCl_3$) of Zn(**3**)$_2$.

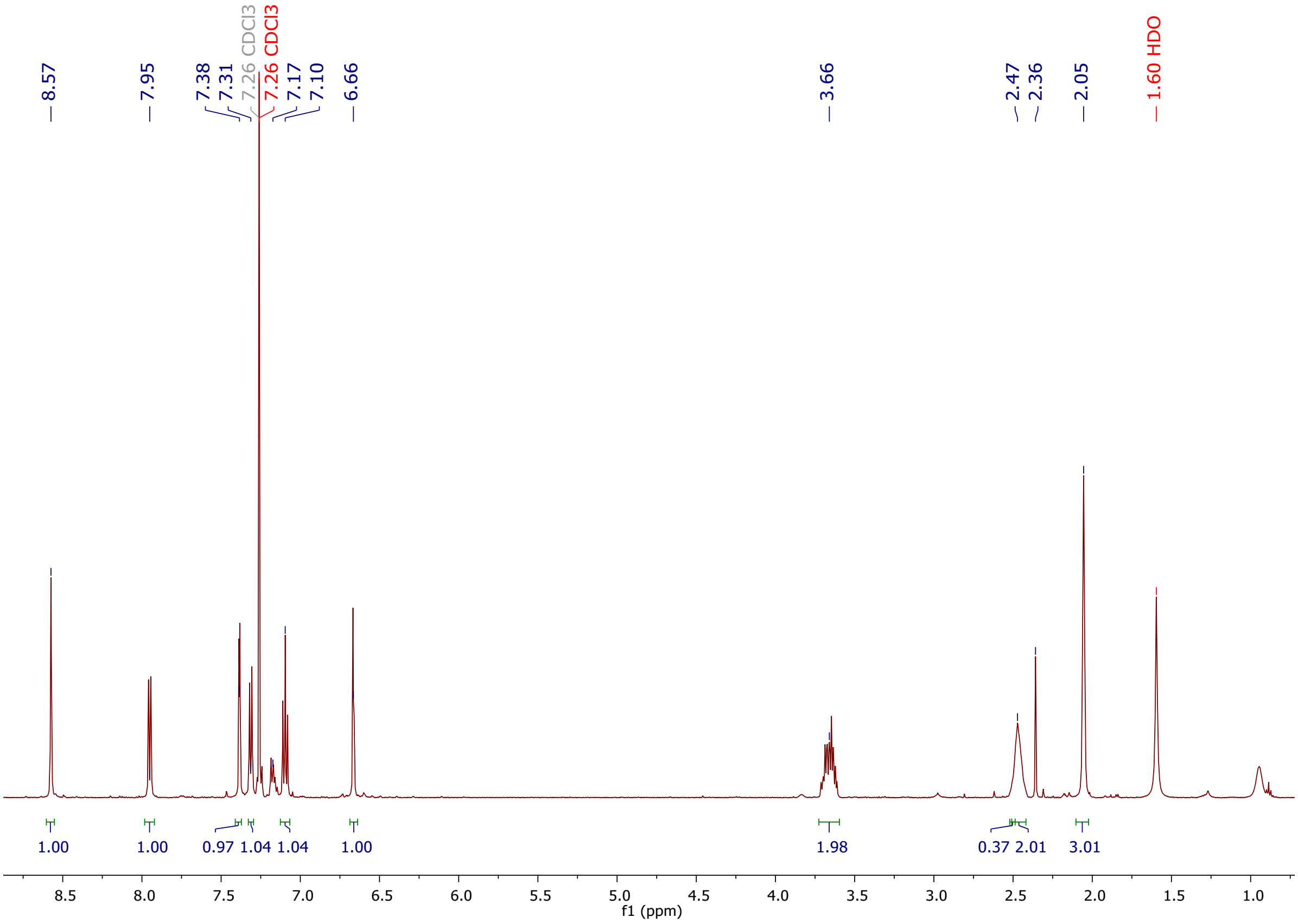

Figure S16 ^{1}H NMR spectrum (500 MHz, $CDCl_3$) of Zn(**3**)$_2$ after one week stored in air.

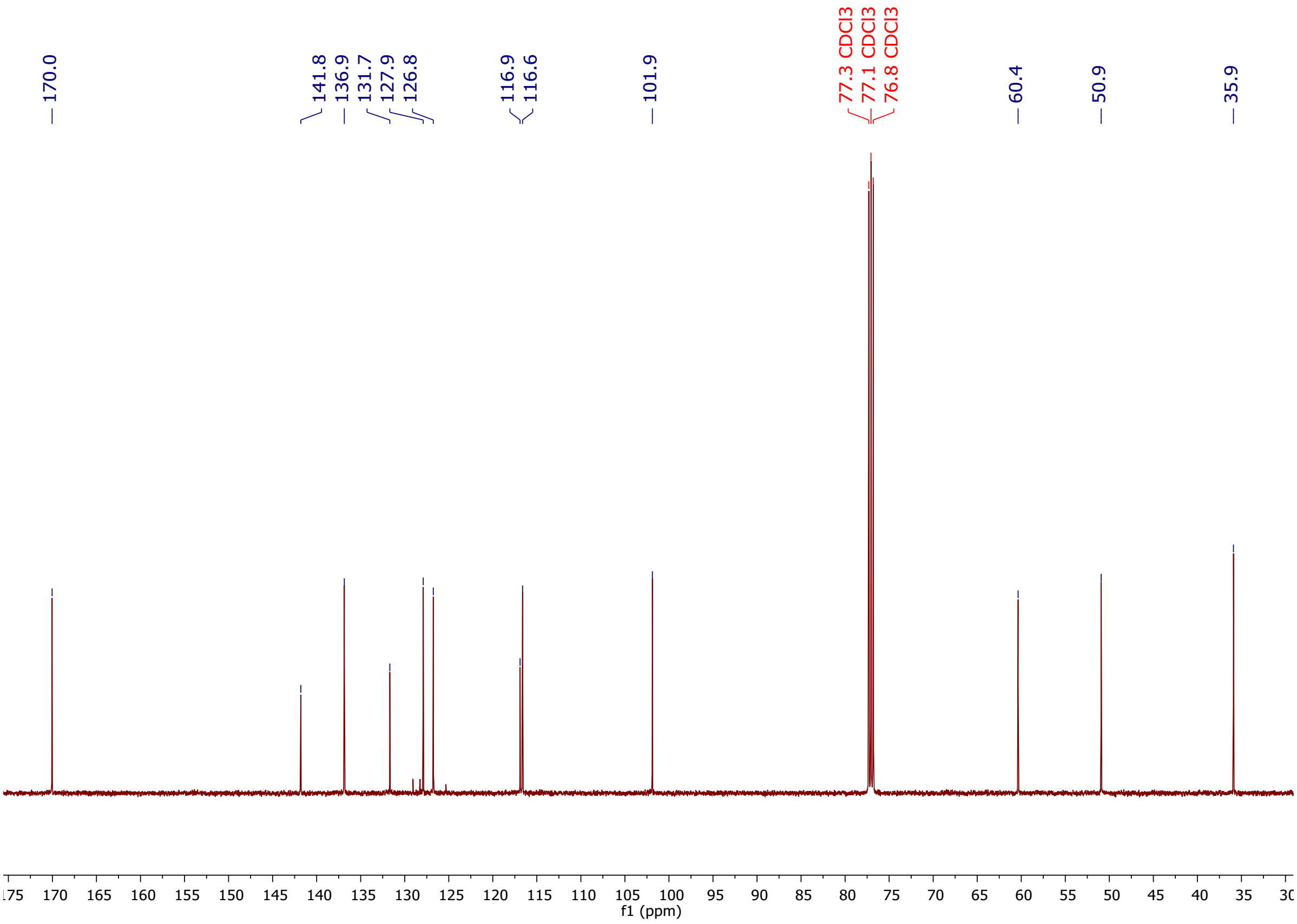

Figure S17 $^{13}C\{^{1}H\}$ NMR spectrum (126 MHz, $CDCl_3$) of Zn(**3**)$_2$.

$Zn(\mathbf{4})_2$

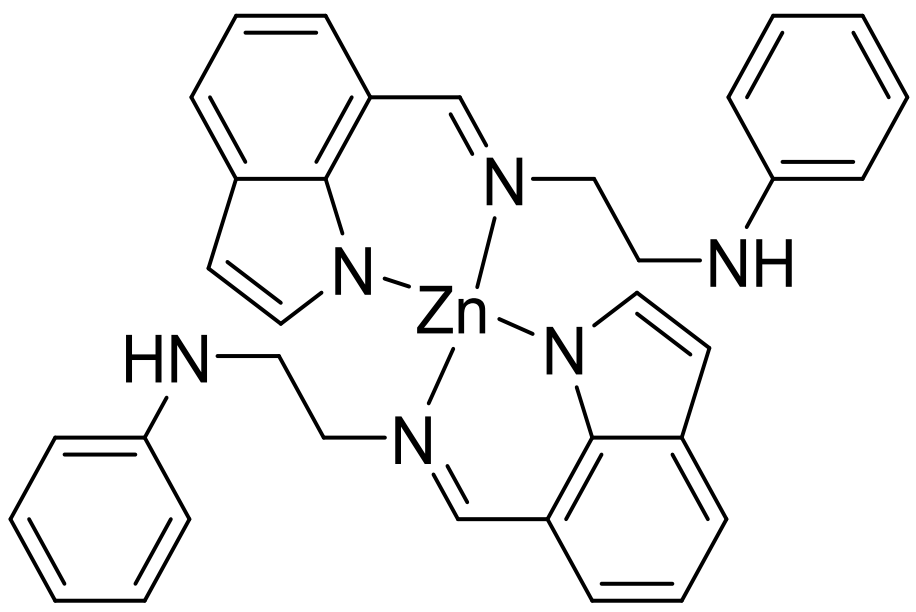

Ligand **4**H (0.503 g, 2 equiv, 2 mmol) was dried in vacuo for two hours and dissolved in anhydrous toluene (10 mL) in a Schlenk flask. $Zn(Et)_2$ (1 mL, 1 equiv, 1 mmol) was added dropwise. The solution was stirred overnight and concentrated to yield $Zn(\mathbf{4})_2$ as a yellow precipitate (0.206 g, 35%).

^{1}H NMR (500 MHz, $CDCl_3$) δ 8.39 (s, 1H, N=C**H**), 8.04 (dd, *J* = 7.8, 1.1 Hz, 1H, Ar**H**), 7.49 (d, *J* = 2.7 Hz, 1H, Ar**H**), 7.32 (dd, *J* = 7.3, 1.1 Hz, 1H, Ar**H**), 7.17 (t, *J* = 7.5 Hz, 1H, Ar**H**), 6.97 (dd, *J* = 8.5, 7.3 Hz, 2H, Ar**H**), 6.76 (d, *J* = 2.7 Hz, 1H, Ar**H**), 6.64 (tt, *J* = 7.3, 1.1 Hz, 1H, Ar**H**), 6.17 – 6.02 (m, 2H, Ar**H**), 3.70 – 3.56 (m, 2H, C**H**$_2$), 3.40 (t, *J* = 6.4 Hz, 1H, N**H**), 3.08 (m, 2H, C**H**$_2$), 3.02 – 2.87 (m, 1H, C**H**$_2$).

^{13}C NMR (126 MHz, $CDCl_3$) δ 171.1 (**C**=N), 146.7 (Ar), 141.7 (Ar), 136.8 (Ar), 131.7 (Ar), 129.3 (Ar), 128.7 (Ar), 127.6 (Ar), 117.8 (Ar), 117.2 (Ar), 116.7 (Ar), 112.8 (Ar), 102.9 (Ar), 59.5 (**C**H$_2$), 43.44 (**C**H$_2$).

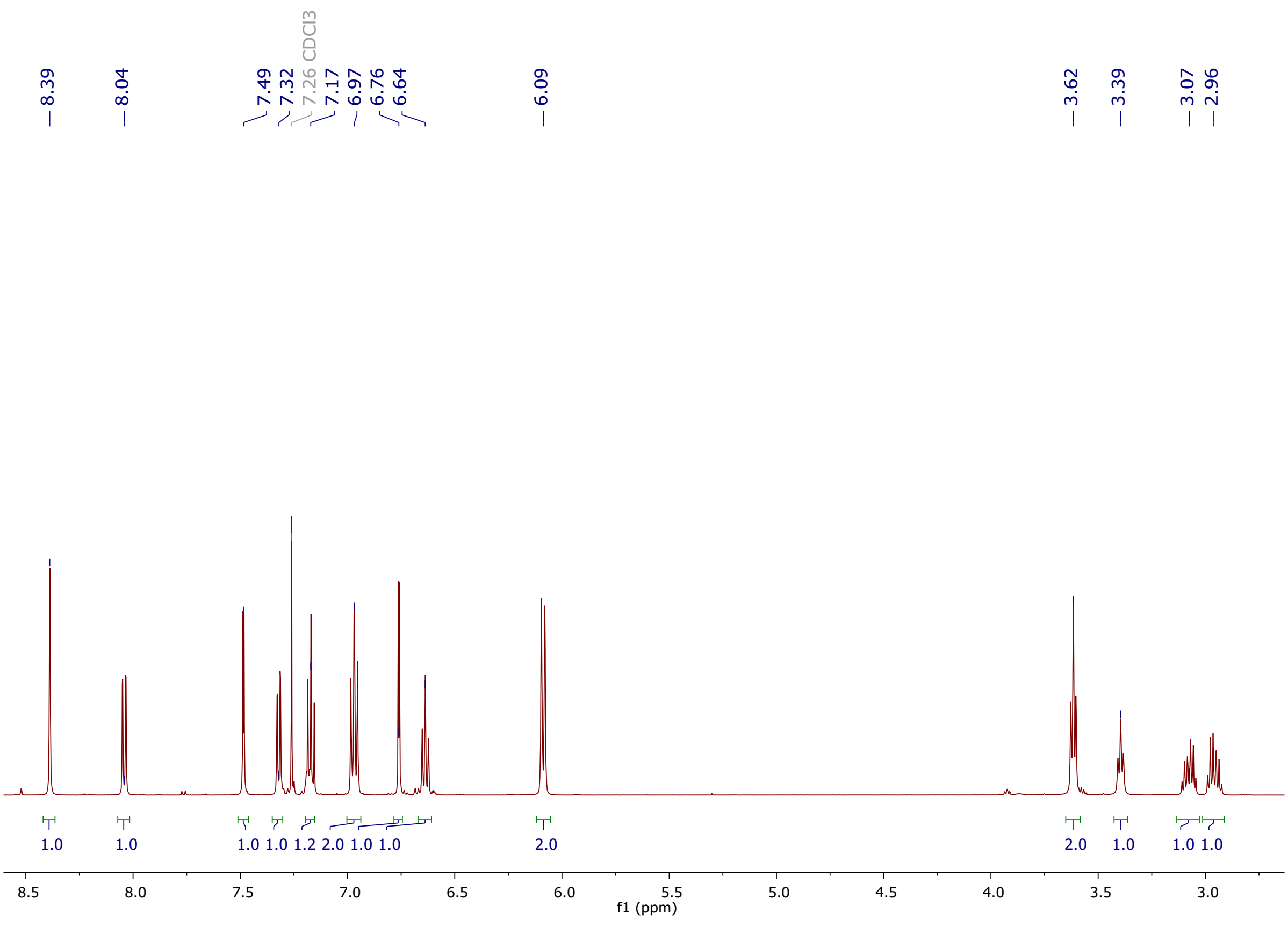

Figure S18 $^{13}C\{^{1}H\}$ NMR spectrum (500 MHz, $CDCl_3$) of Zn(**4**)$_2$.

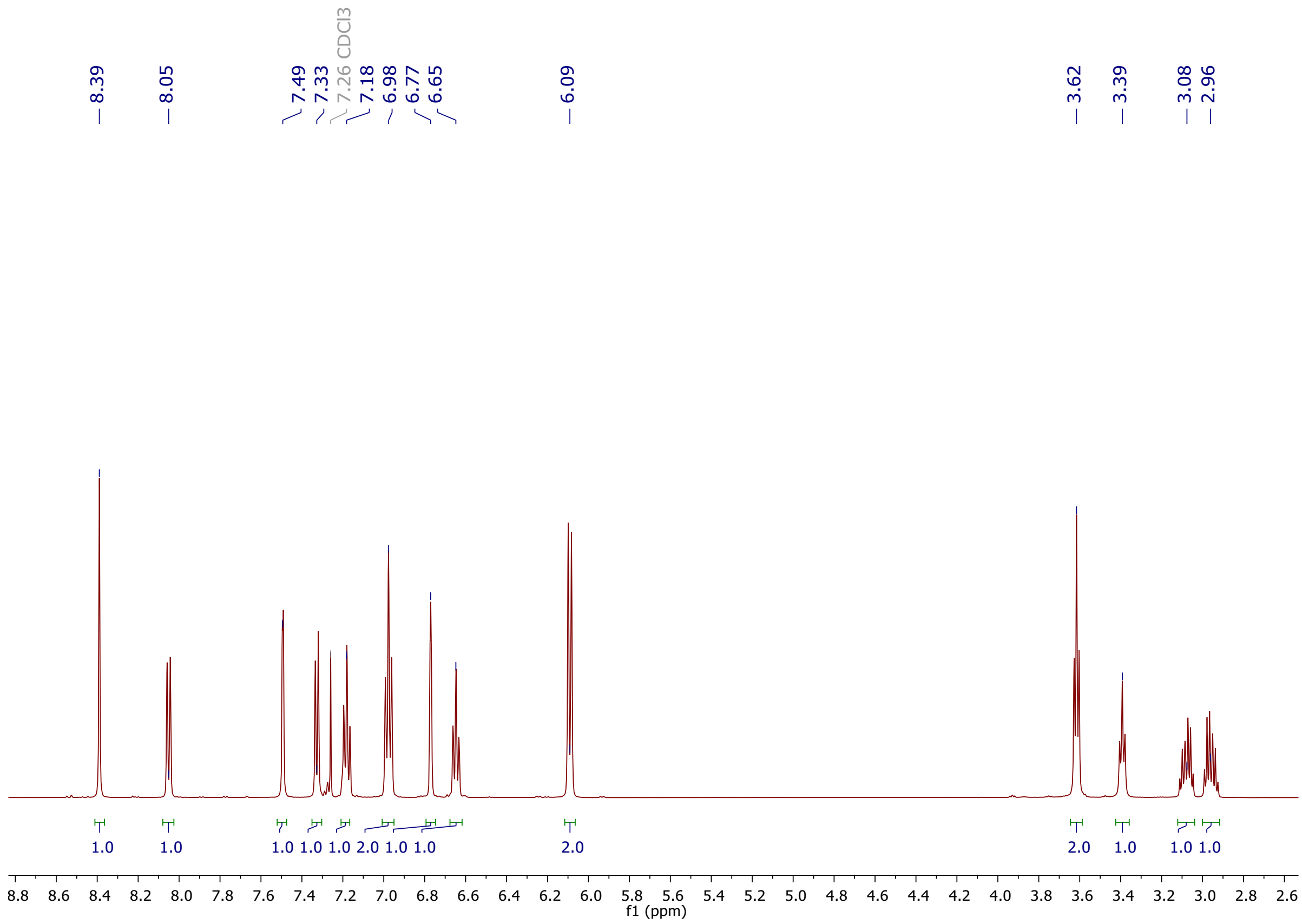

Figure S19 ^{1}H NMR spectrum (500 MHz, $CDCl_3$) of Zn(**4**)$_2$ after one week stored in air.

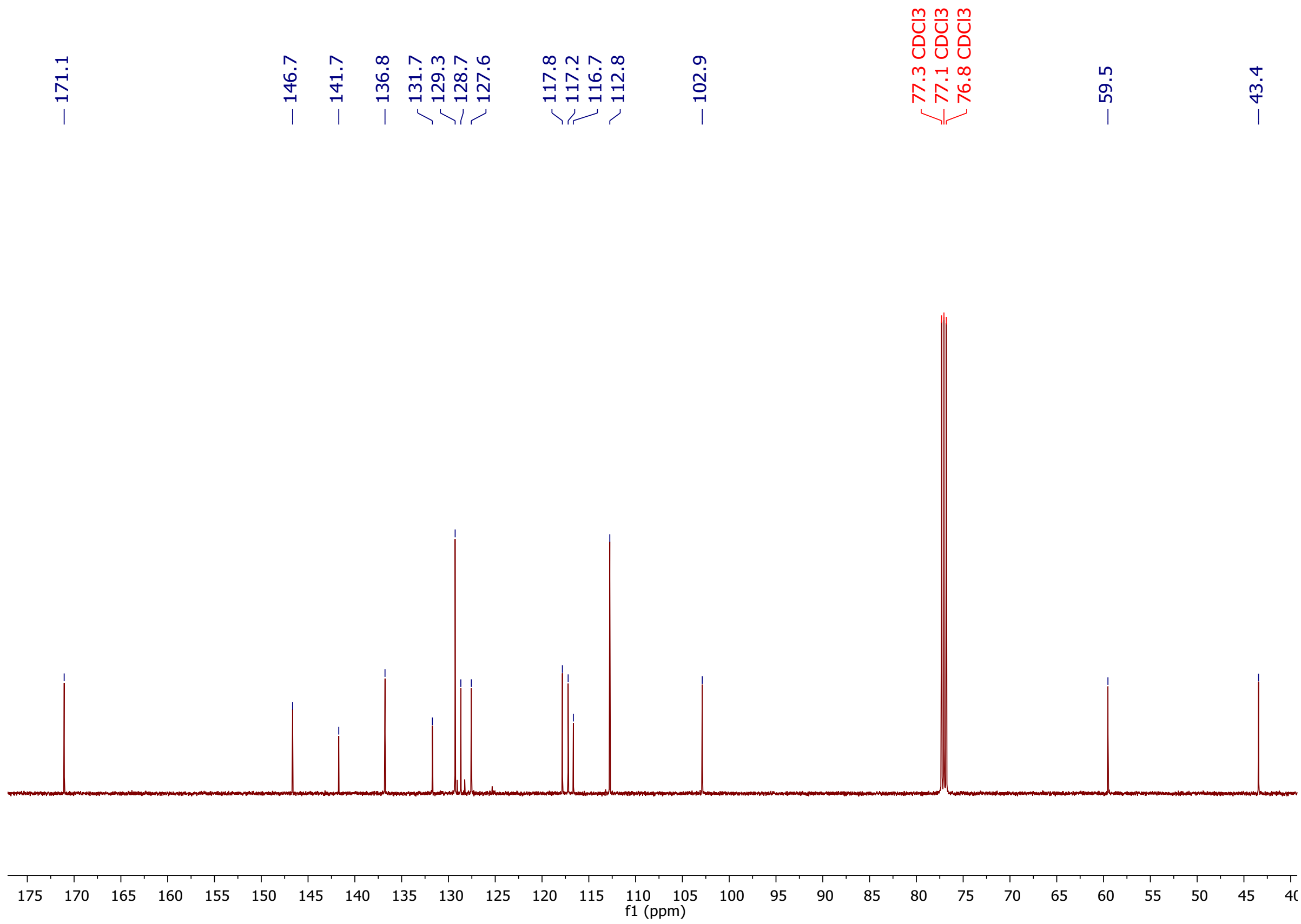

Figure S20 $^{13}C\{^{1}H\}$ NMR spectrum (126 MHz, $CDCl_3$) of Zn(**4**)$_2$.

1.3 X-ray crystallography

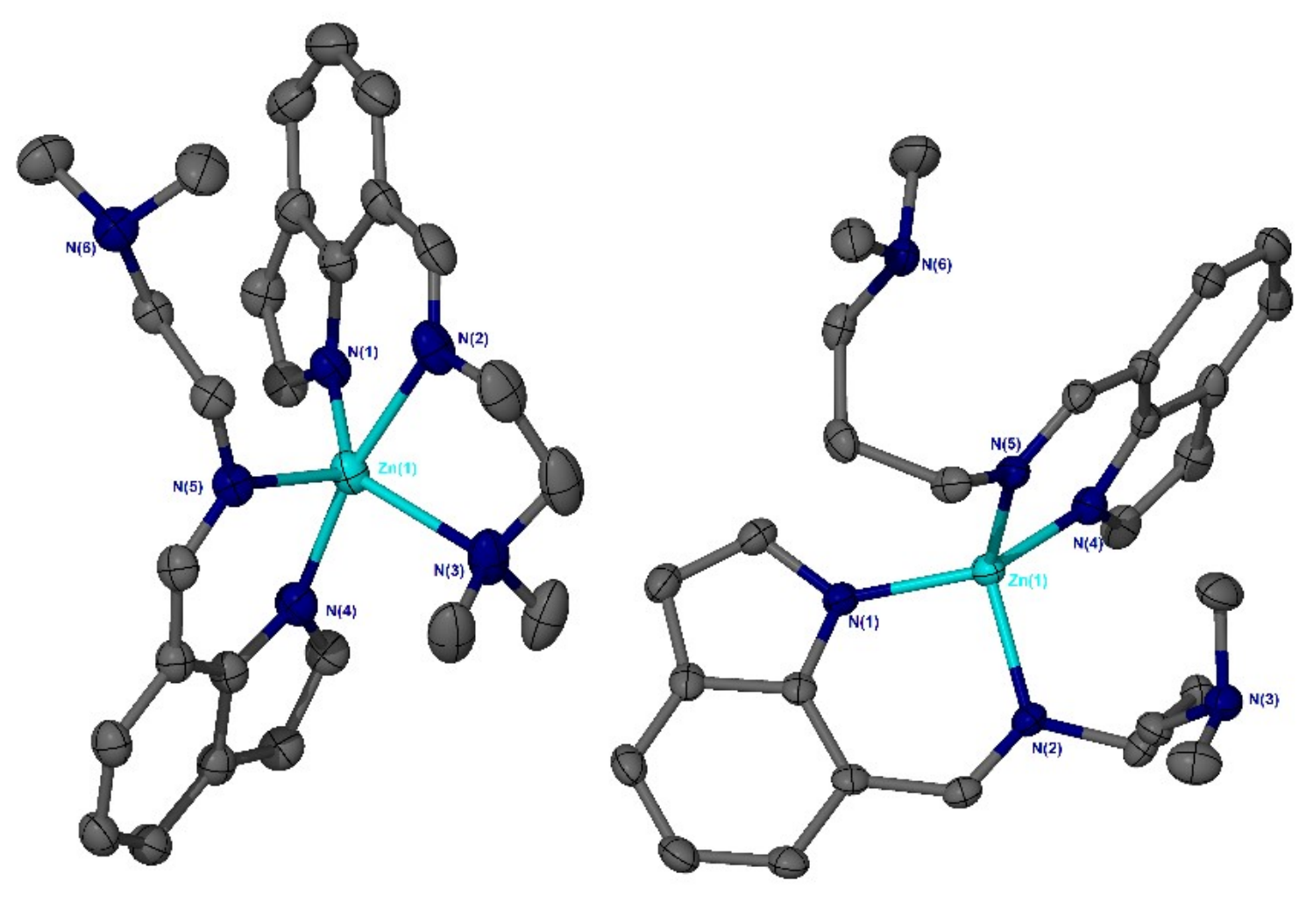

Figure S21 Solid-state structures of Zn(**1**)$_2$ (left) and Zn(**2**)$_2$ (right). Ellipsoids are shown at the 30% probability level. Hydrogen atoms have been removed for clarity.

2. Solution alcoholysis of PLA and BPA-PC

2.1 PLA methanolysis – conversion, selectivity and yield

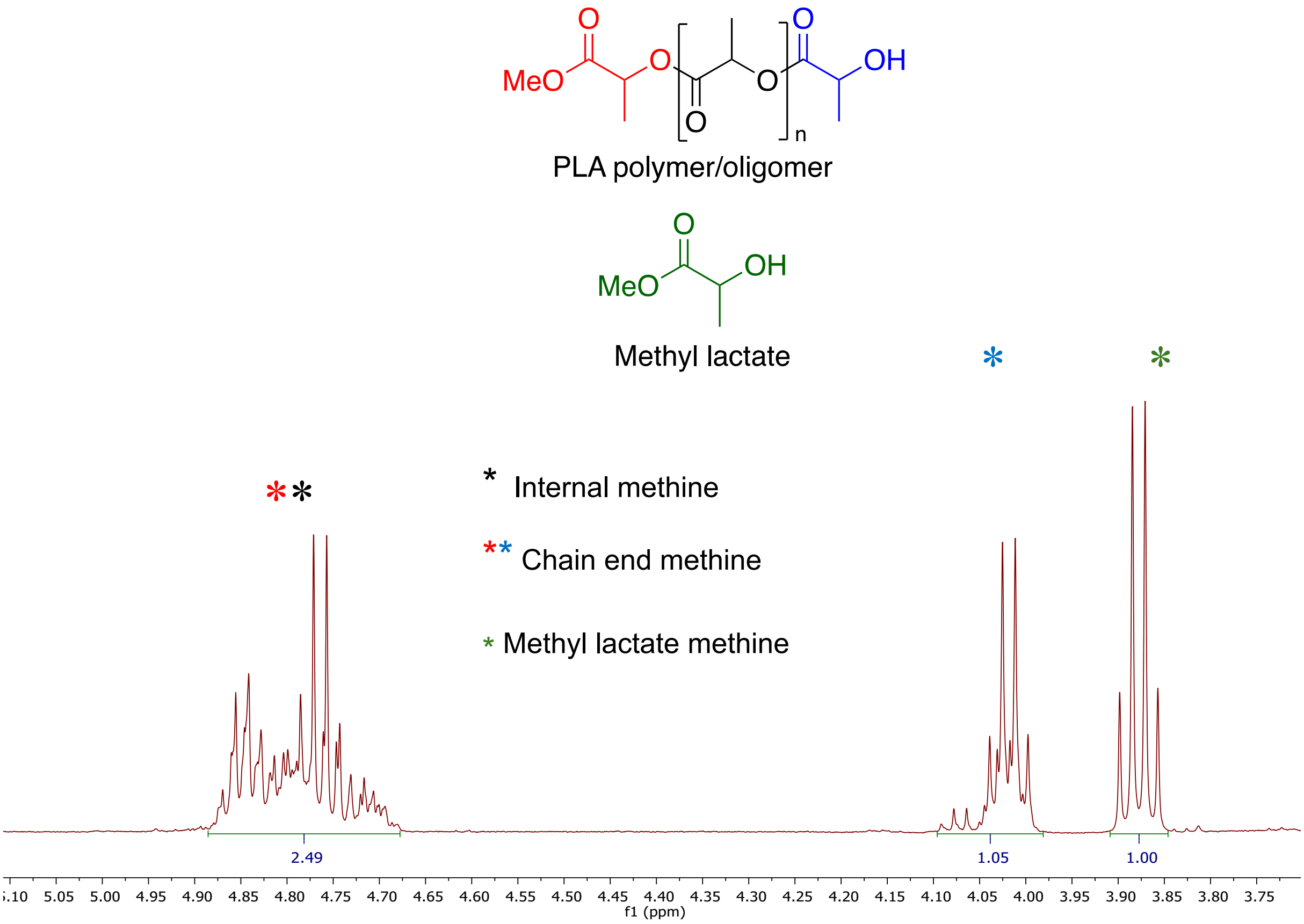

Figure S22 ^{1}H NMR ($CDCl_3$, 500MHz, δ = 5.2 – 4.0 ppm) spectrum of PLA degradation into MeLA using Zn(**3**)$_2$.

$$[Me-LA] = \frac{1}{(1+1.05+2.49)} x\ 100 = 22\%$$

$$[Int] = \frac{(2.49-1.05)}{(1+1.17+2.49)} x\ 100 = 31\%$$

$$[CE] = \frac{1.05}{(1+1.05+2.49)} x\ 100 = 23\%$$

$$[CE] = \frac{1.05}{(1+1.05+2.49)} x\ 100 = 23\%$$

Utilising the relative quantities shown in **Figure S22**, the following key parameters can be calculated from equations (1–3). The conversion of internal methine (X_{int}) represents the overall conversion of PLA to oligomers and methyl lactate. The methyl lactate yield (Y_{MeLA}) and selectivity (S_{MeLA}) are used to monitor the completion of the reaction to methyl lactate.

$$X_{int} = 1 - \frac{[int]}{[int]_0} \qquad \textbf{(1)}$$

$$S_{Me-LA} = \frac{[Me-LA]}{[int]_0 - [int]} \qquad \textbf{(2)}$$

$$Y_{Me-LA} = X_{int} S_{Me-LA} \qquad \textbf{(3)}$$

2.2 PLA methanolysis – results and kinetics

Cat.	Time [min]	Loading [wt%]	$Y_{Me\text{-}LA}$ [%][b]	$S_{Me\text{-}LA}$ [%][b]	$X_{Me\text{-}LA}$ [%][b]	K_{app} [min^{-1}]
Zn(**1**)$_2$	270	8	40	47	87	0.007
Zn(**2**)$_2$	90	8	74	75	99	0.046
Zn(**3**)$_2$	60	8	69	70	93	0.042
Zn(**4**)$_2$	300	8	79	79	100	0.018

Table S1 Degradation of PLLA cup to methyl lactate *via* methanolysis using Zn(**1 – 4**)$_2$ at 80 °C.[a]

[a] Reaction conditions: 0.25 g of PLLA cup (M_n = 45,510 g mol^{-1}), V_{THF}:V_{MeOH} = 4 : 1, n_{MeOH}:n_{ester} = 7 : 1, 8 wt% cat. loading (20 mg, 0.98 – 1.24 mol% relative to ester linkages). [b] ^{1}H NMR (400 MHz, 298 K, $CDCl_3$) spectroscopy used to calculate Y_{MeLA}, S_{MeLA} and X_{Int}.

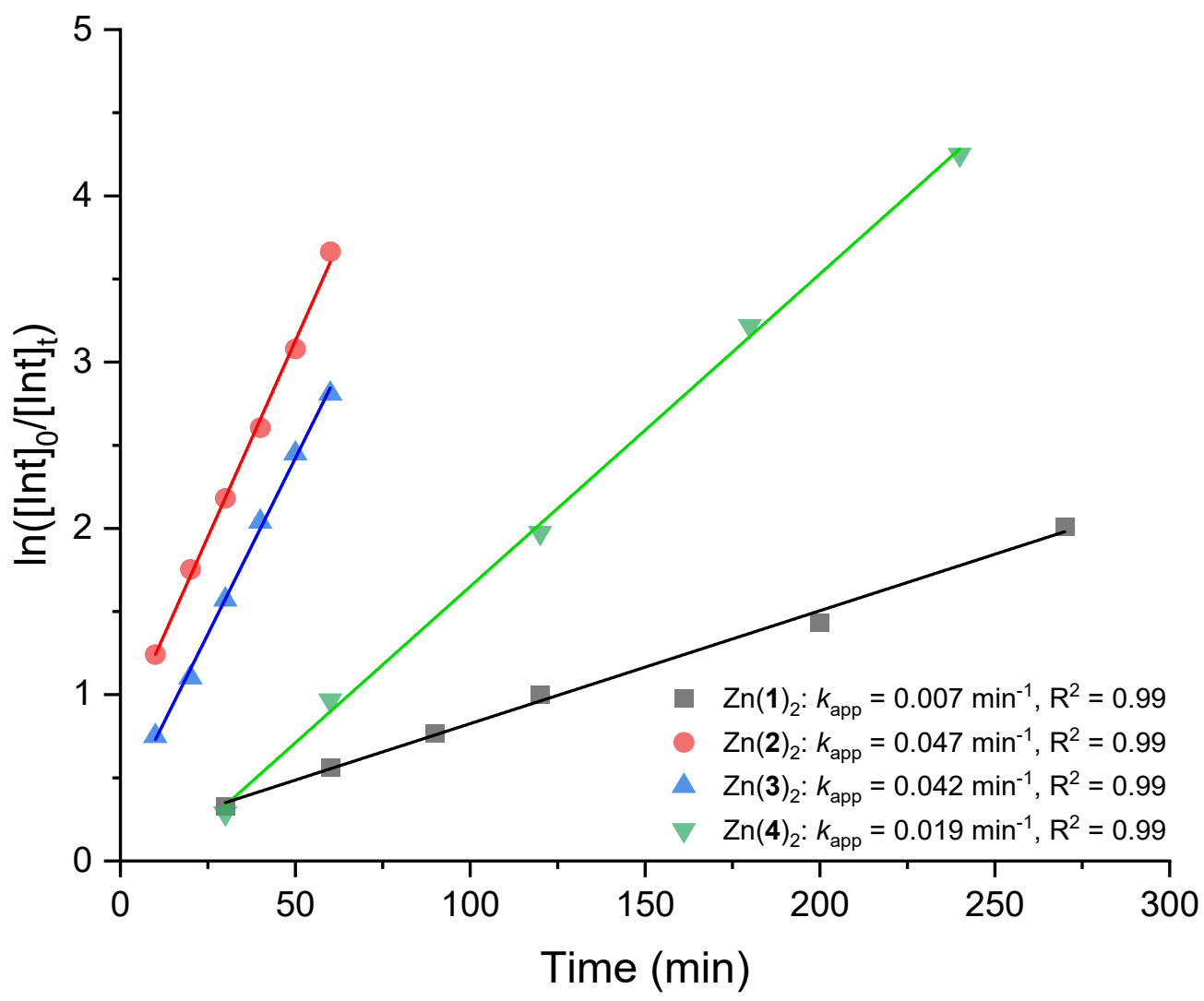

Figure S23 *Pseudo*-first order kinetics of PLA methanolysis with 8 wt% Zn(**1–4**)$_2$ at 80 °C.

2.3 BPA-PC methanolysis in THF

Cat.	Time [h]	Loading [wt%]	S_{BPA} [%][b]	$S_{DC\text{-}BPA + MC\text{-}BPA}$ [%][b]	Y_{BPA} (Isolated) [%][c]
Zn(**1**)$_2$	1	4	38	62	-
Zn(**2**)$_2$	1	4	81	19	72
Zn(**3**)$_2$	1	4	87	13	80
Zn(**4**)$_2$	1	4	47	53	-

Table S2 Degradation of BPA-PC to BPA *via* methanolysis using Zn(**1 – 4**)$_2$ at 75 °C, 17.5 eq. MeOH.[a]

[a] Reaction conditions: 0.25 g BPA-PC pellets (M_n = 45,000 g mol^{-1}), 17.5 equivalents of MeOH (relative to carbonate linkages), 4 wt% catalyst loading (10 mg, 1.7 – 2.2 mol% based on carbonate linkages). [b] Selectivities based on ^{1}H NMR spectroscopy. [c] BPA was recrystallised from water and dried to constant weight.

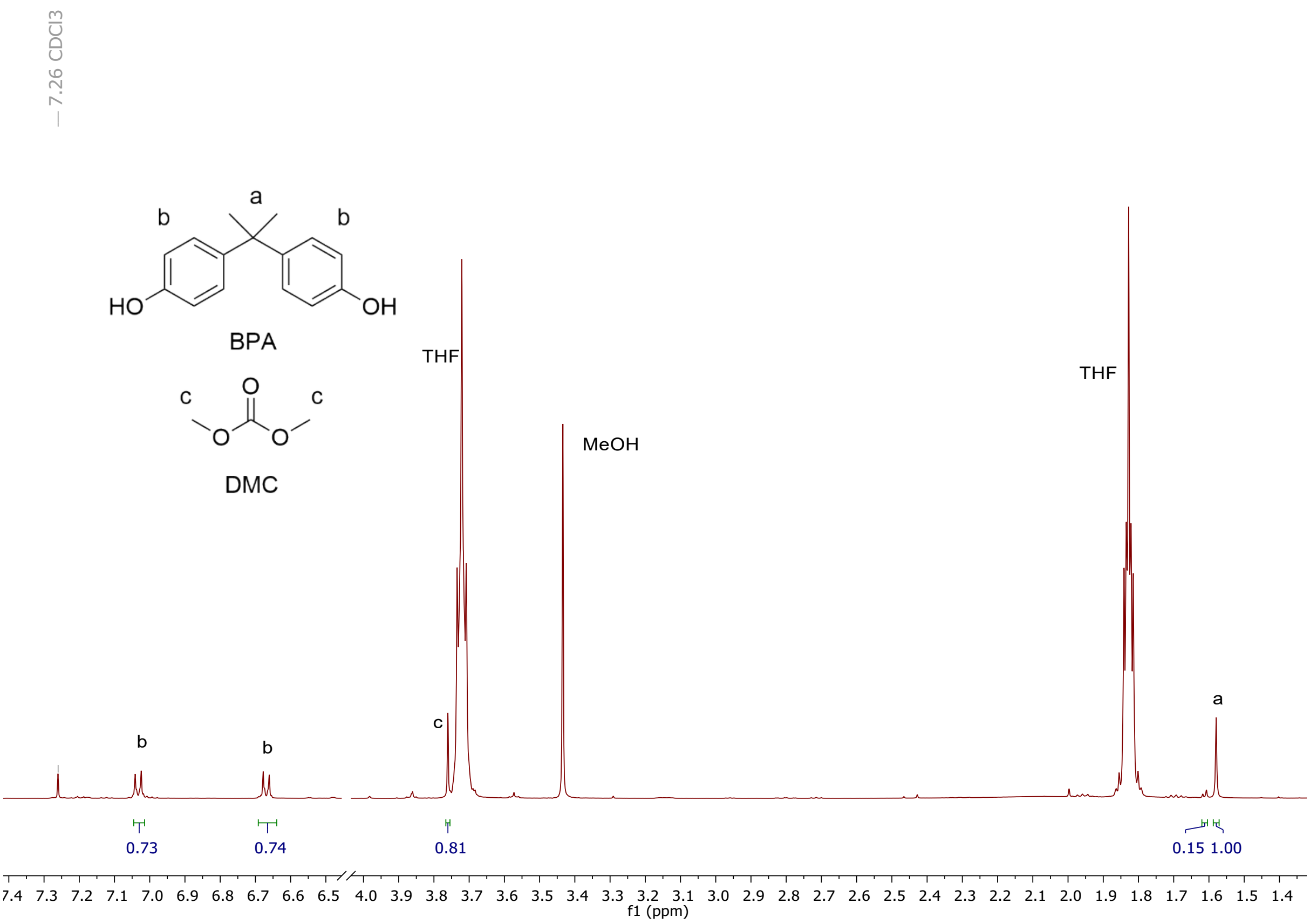

Figure S24 ^{1}H NMR ($CDCl_3$, 500 MHz) spectrum of BPA-PC (M_n = 45,000 gmol^{-1}) methanolysis (17.5 equivalents MeOH) aliquot after one hour at 75 °C in THF. Selectivities calculated from literature peak examples from the methyl region (δ = 1.60 ppm).

3. Co-solvent-free alcoholysis of polymers

3.1 PLA alcoholysis in neat alcohol

Cat.	Temp. [°C]	Loading [wt%]	Time (min)[a]	X_{int} [%][b]	S_{MeLA} [%][b]	Y_{MeLA} [%][b]	TOF [h^{-1}]
Zn(**2**)$_2$	Reflux	4	60	91	64	58	160
Zn(**3**)$_2$	Reflux	4	45	93	82	76	200

Table S3 Degradation of PLLA *via* methanolysis in air using Zn(**2 – 3**)$_2$ in neat methanol at reflux in air.[a]

[a] Reaction conditions: 0.25 g of PLLA cup (M_n = 45,510 g mol^{-1}), MeOH (2 mL, n_{MeOH}/n_{ester} = 14 : 1), 4 wt% cat. loading (10 mg, 0.55 – 0.62 mol% relative to ester linkages). [b] ^{1}H NMR (400 MHz, 298 K, $CDCl_3$) spectroscopy used to calculate Y_{MeLA}, S_{MeLA} and X_{Int}.

Cat.	ROH	Temp. [°C]	Loading [wt%]	Time (min)[a]	X_{int} [%]	S_{MeLA} [%]	Y_{MeLA} [%]	TOF [h^{-1}]
Zn(**2**)$_2$	EtOH	130	4	60	48	22	11	90
Zn(**3**)$_2$	EtOH	130	4	60	81	45	37	130

Table S4 Degradation of PLLA cup *via* ethanolysis using Zn(**2** – **3**)$_2$ in neat ethanol at 130 °C.

[a] Reaction conditions: 0.25 g of PLLA cup (M_n = 45,510 g mol^{-1}), EtOH (2.9 mL, n_{MeOH}/n_{ester} = 14 : 1), 4 wt% cat. loading (10 mg, 0.55 – 0.62 mol% relative to ester linkages). [b] ^{1}H NMR (400 MHz, 298 K, $CDCl_3$) spectroscopy used to calculate Y_{MeLA}, S_{MeLA} and X_{Int}.

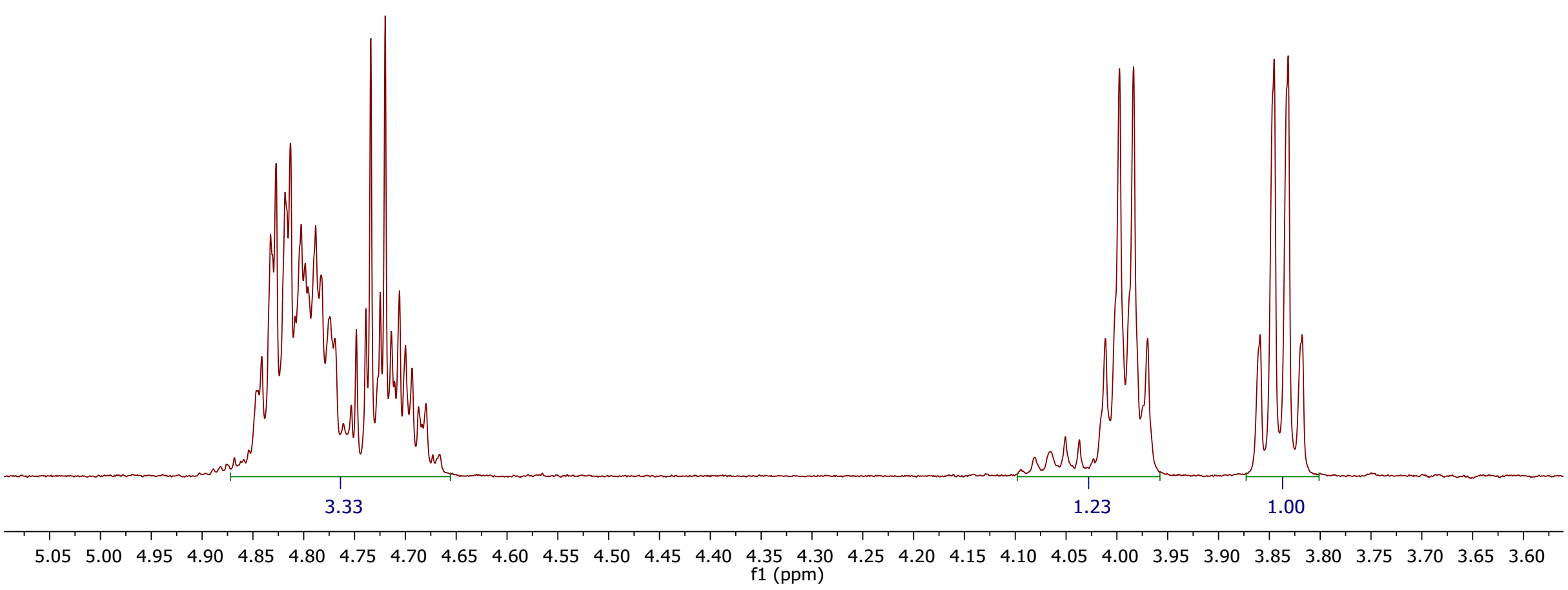

Figure S25 ^{1}H NMR (C_6D_6, 500 MHz) spectrum of PLA (M_n = 45,510 gmol^{-1}) methanolysis (14 equiv. MeOH) aliquot after three minutes at 130 °C in neat methanol.

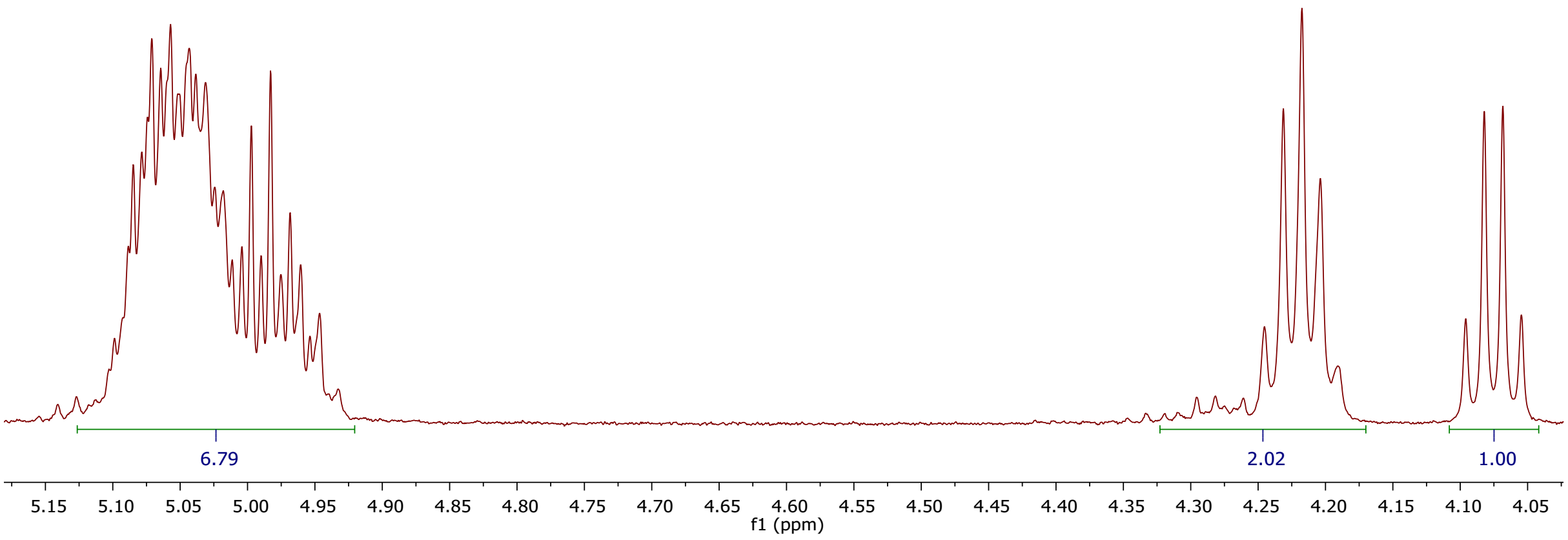

Figure S26 ^{1}H NMR (C_6D_6, 500 MHz) spectrum of PLA (M_n = 45,510 gmol^{-1}) ethanolysis (14 equiv. EtOH) aliquot after one hour at 130 °C in neat ethanol.

3.2 BPA-PC alcoholysis in neat methanol

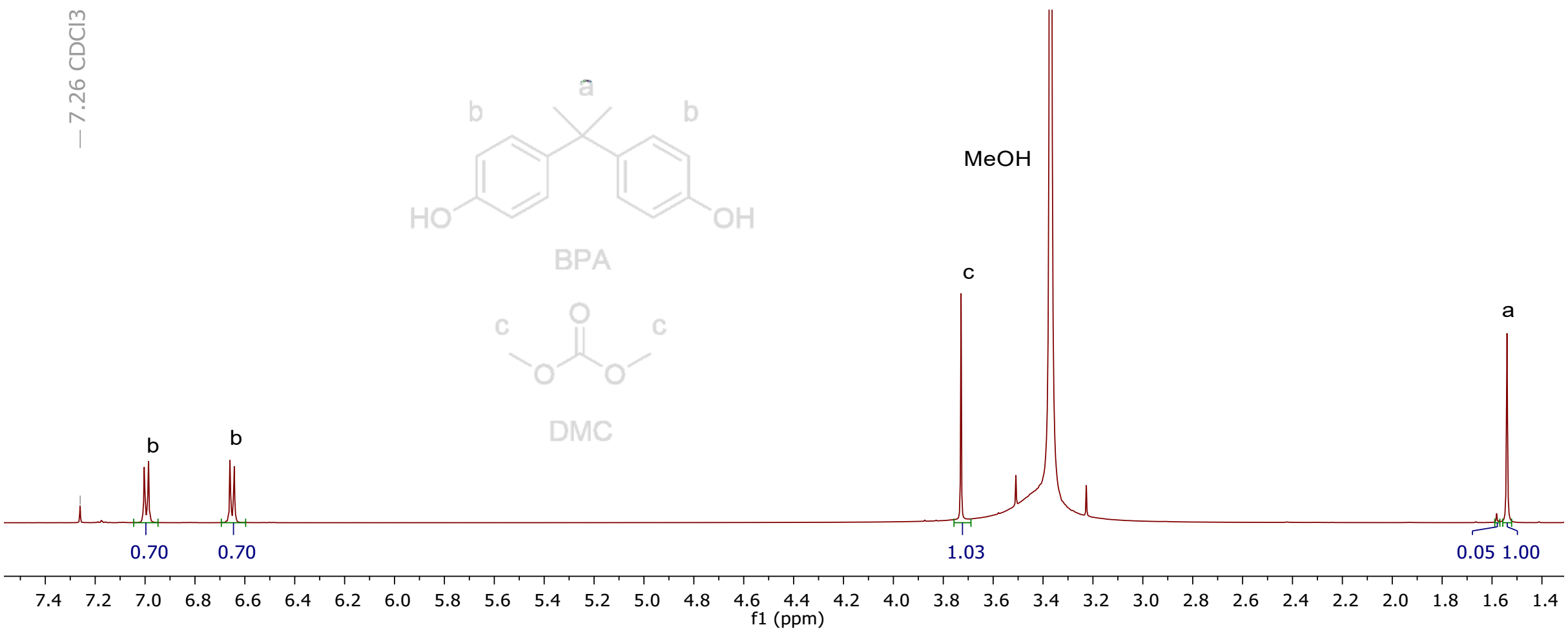

Figure S27 ^{1}H NMR ($CDCl_3$, 500 MHz) spectrum of BPA-PC (M_n = 45,000 gmol^{-1}) methanolysis (50 equivalents MeOH) aliquot after one hour at 130 °C in MeOH. Selectivities calculated from literature peak examples from the methyl region (δ = 1.60 ppm).

3.3 PET glycolysis

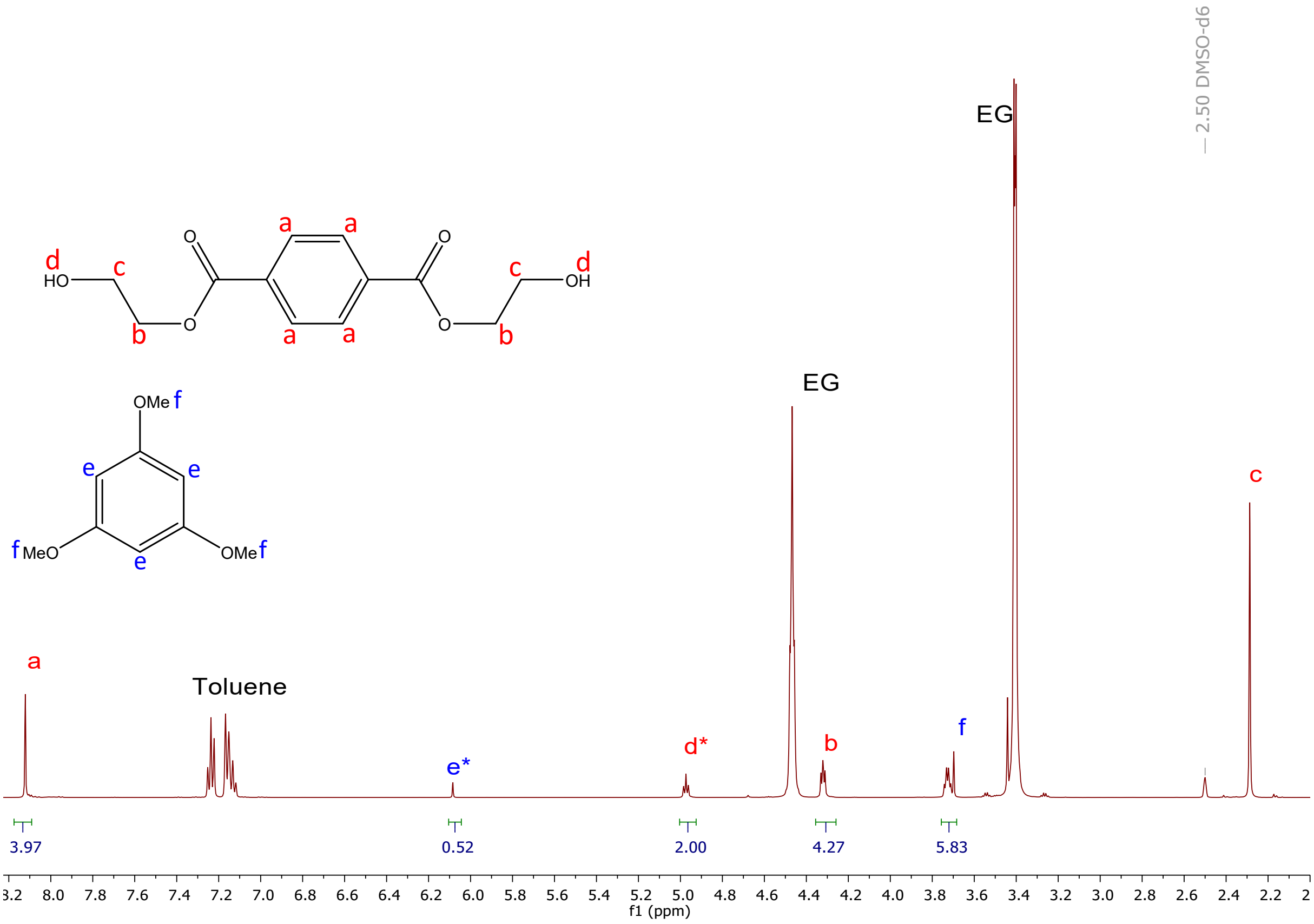

Figure S28 ^{1}H NMR (400 MHz, DMSO-d6) spectrum for PET degradation with internal standard (trimethoxybenzene in toluene) added. Starred peaks were used for determination of BHET yield.

4. Chemical recycling to monomer

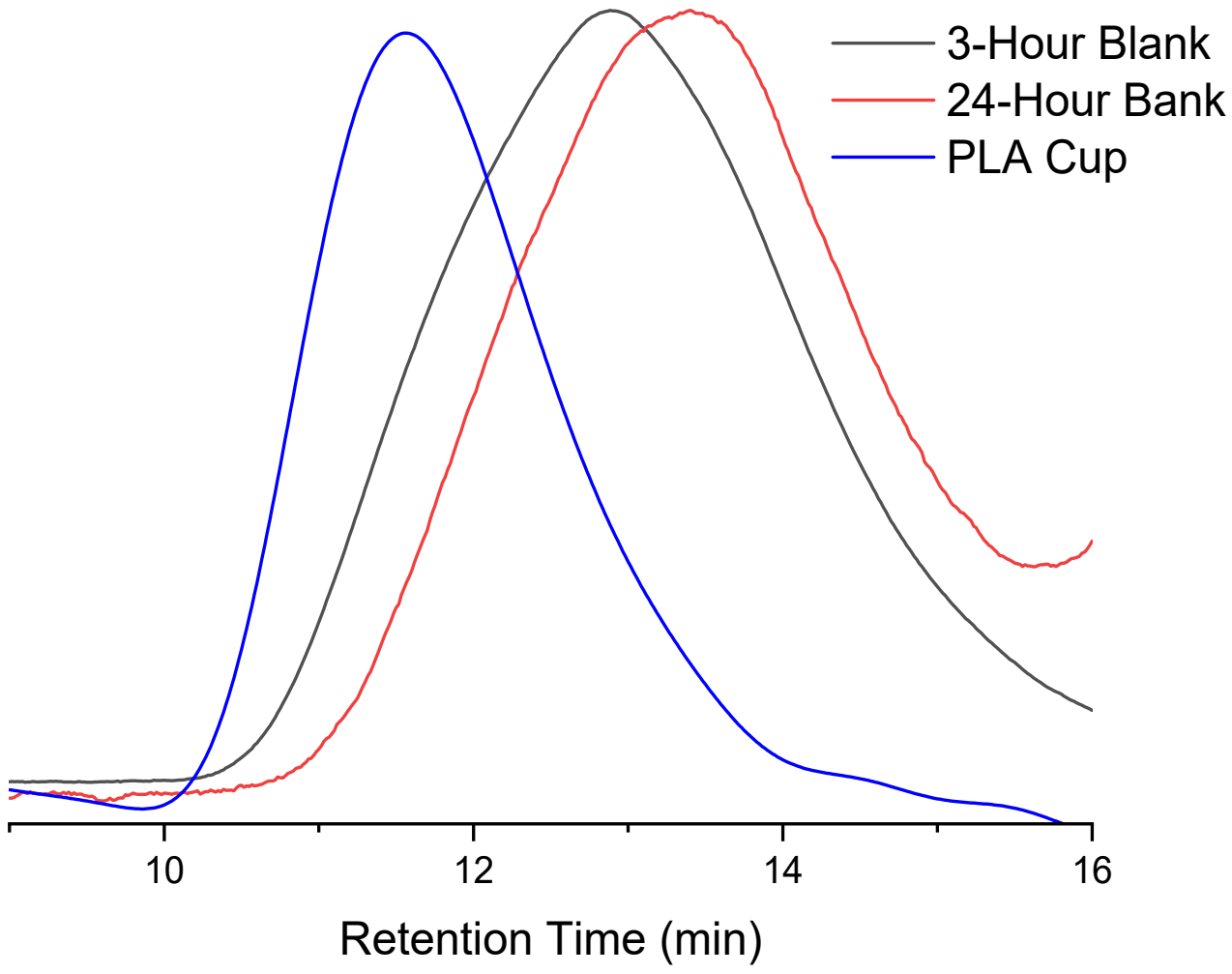

Figure S29 GPC traces of PLA cup (M_n = 56,000 g mol^{-1}), 3-hour blank (21,900 g mol^{-1}) and 24-hour blank (16,100 g mol^{-1})

5. Chemical recycling of mixed plastics

5.1 PLA/BPA-PC selectivity optimisation

Table S5 Degradation of PLA to MeLA in the presence of BPA-PC in neat methanol at 60 – 100 °C with 4 wt% Zn(**3**)$_2$.

				BPA-PC			PLA	
Entry	Temp [°C]	Loading [wt%]	Time [min]	Mass Loss [%][d]	S_{BPA} [%][b]	Y_{BPA} [%][c]	X_{int} [%]b	Y_{MeLA} [%][b]
1	100	8	10	12	96	12	100	100
2	90	8	20	15	89	13	99	95
3	80	8	30	6	86	5	100	96
4	70	8	90	<1	65	<1	99	97
5	60	8	165	<1	49	<1	97	85

[a] Reaction conditions: 0.125 g of PLLA cup (M_n = 45,510 g mol^{-1}), 0.125 g BPA-PC (Pellets: M_n ≈ 45,000 g mol^{-1}), MeOH (1 mL, n_{MeOH}/n_{ester} = 14 : 1 w.r.t. PLA), 8 wt% cat. loading (10 mg, 1.24 mol% relative to ester linkages). [b] ^{1}H NMR (400 MHz, 298 K, $CDCl_3$) spectroscopy used to calculate Y_{MeLA}, X_{int} and S_{BPA}. [c] BPA yield was calculated from the product of mass loss (%) and selectivity to BPA (%) divided by 100. [d] Remaining BPA-PC was collected, dried and weighed to determine mass loss.

Table S6 Degradation of PLA to MeLA in the presence of BPA-PC in neat methanol at 80 °C with 2 – 16 wt% Zn(**3**)$_2$.

				BPA-PC			PLA	
Entry	Temp [°C]	Loading [wt%]	Time [min]	Mass Loss [%][d]	S_{BPA} [%][b]	Y_{BPA} [%][c]	X_{int} [%]b	Y_{MeLA} [%][b]
1	80	16	30	6	80	5	100	99
2	80	8	30	6	86	5	100	96
3	80	4	30	5	93	5	96	79
4	80	2	30	5	94	5	100	99

[a] Reaction conditions: 0.125 g of PLLA cup (M_n = 45,510 g mol^{-1}), 0.125 g BPA-PC (Pellets: M_n ≈ 45,000 g mol^{-1}), MeOH (1 mL, n_{MeOH}/n_{ester} = 14 : 1 w.r.t. PLA), 2 – 8 wt% cat. loading (2.5 – 40 mg, 0.31 – 2.51 mol% relative to ester linkages). [b] ^{1}H NMR (400 MHz, 298 K, $CDCl_3$) spectroscopy used to calculate Y_{MeLA}, X_{int} and S_{BPA}. [c] BPA yield was calculated from the product

of mass loss (%) and selectivity to BPA (%) divided by 100. [d] Remaining BPA-PC was collected, dried and weighed to determine mass loss.

Table S7 Degradation of PLA to MeLA in the presence of BPA-PC in neat methanol at 70 °C with 2 – 16 wt% Zn(**3**)$_2$.

				BPA-PC			PLA	
Entry	Temp [°C]	Loading [wt%]	Time [min]	Mass Loss [%][d]	S_{BPA} [%][b]	Y_{BPA} [%][c]	X_{int} [%]b	Y_{MeLA} [%][b]
1	70	16	45	1	84	<1	100	100
2	70	8	90	<1	65	<1	99	97
3	70	4	90	1	93	<1	100	100
4	70	2	120	1	80	<1	100	99
5	70	4	1440	15	87	13	100	100

[a] Reaction conditions: 0.125 g of PLLA cup (M_n = 45,510 g mol^{-1}), 0.125 g BPA-PC (Pellets: M_n ≈ 45,000 g mol^{-1}), MeOH (1 mL, n_{MeOH}/n_{ester} = 14 : 1 w.r.t. PLA), 2 – 8 wt% cat. loading (2.5 –4 0 mg, 0.31 – 2.51 mol% relative to ester linkages). [b] ^{1}H NMR (400 MHz, 298 K, $CDCl_3$) spectroscopy used to calculate Y_{MeLA}, X_{Int} and S_{BPA}. [c] BPA yield was calculated from the product of mass loss (%) and selectivity to BPA (%) divided by 100. [d] Remaining BPA-PC was collected, dried and weighed to determine mass loss.

5.2 Three component chemical recycling

			PLA[a]			BPA-PC[b]			PET[c]	
Entry	Temp [°C]	Time [min]	Mass Loss[d] [%]	X_{int} [%][e]	Y_{MeLA} [%][e]	Mass Loss [%][d]	S_{BPA} [%][e]	Y_{BPA} [%][e]	Mass Loss [%][d]	Y_{BHET} [%][f]
1	70	15	7	7	7	<1	-	-	<1	-
2	70	30	28	27	24	<1	-	-	<1	-
3	70	45	49	49	46	<1	-	-	<1	-
4	70	60	64	64	63	<1	-	-	<1	-
5	70	75	77	77	77	<1	-	-	<1	-
6	70	90	94	94	90	<1	-	-	<1	-
7	70	120	>99	>99	97	1	85	<1	<1	-
8	100	5	100	100	100	10	69	7	<1	-
9	100	10	100	100	100	21	78	16	<1	-
10	100	15	100	100	100	29	90	26	<1	-
11	100	20	100	100	100	54	80	43	<1	-
12	100	30	100	100	100	86	90	77	<1	-
13	100	45	100	100	100	96	95	91	<1	-
14	100	60	100	100	100	100	98	98	<1	-
15	180	15	100	100	100	100	100	100	4	3
16	180	30	100	100	100	100	100	100	13	11
17	180	45	100	100	100	100	100	100	22	20
18	180	60	100	100	100	100	100	100	43	41
19	180	75	100	100	100	100	100	100	55	55
20	180	90	100	100	100	100	100	100	66	67
21	180	120	100	100	100	100	100	100	100	81
22	180	150	100	100	100	100	100	100	100	93
23	180	180	100	100	100	100	100	100	100	>99

Table S8 Sequential chemical recycling of PLA, BPA-PC and PET with Zn(**3**)$_2$.

[a] Conditions (PLA methanolysis): 0.1 g of PLLA cup (M_n = 45,510 g mol^{-1}), 0.1 g BPA-PC (Pellets: M_n ≈ 45,000 g mol^{-1}), 0.1 g of bottle-grade PET (M_n ≈ 40 000 g mol^{-1}), 8 wt% cat. loading with respect to PLA (8 mg, 0.63 mol% relative to PLA ester linkages), MeOH (1.6 mL, n_{MeOH}:n_{ester} = 14 : 1 w.r.t PLA), 70 °C, 0 – 120 min. [b] Conditions (BPA-PC methanolysis): PLA methanolysis conditions for 120 min then 100 °C for 5 – 60 min. [c] Conditions (PET glycolysis): PLA methanolysis conditions for 120 min, BPA-PC methanolysis conditions for 60 min then volatiles removed, EG (1.2 mL, n_{MeOH}:n_{ester} = 20.6 : 1) added, 180 °C, 15 – 180 min. [d] After each reaction, remaining polymer was dried, separated and weighed to determine mass loss. [e] X_{int}, Y_{MeLA} and Y_{BPA} were calculated from ^{1}H NMR spectroscopy and mass loss. [f] ^{1}H NMR spectroscopy with an internal standard used was for BHET quantification.

6. Green metrics calculations

Process Mass Intensity (PMI) was calculated through summing the total mass of chemicals used in the reaction and dividing by the total mass of products. Methanol is included as a product as it was collected by distillation during the work up.

E-factor was calculated by dividing the total mass of waste produced by the total mass of products. In this case, only methanol that was not collected by distillation or used up in the reaction is included as waste.

Process Mass Intensity				
**Input**	**Mass [g]**		**Output**	**Mass [g]**
Polymer	15.0		MeLA	7.1
Catalyst	1.2		BPA	4.0
Methanol	31.7		BHET	6.2
Ethylene Glycol	33.3		Methanol	21
Water	40			
Total	121.2		Total	38.3

$$PMI = \frac{mass\ of\ inputs}{mass\ of\ products}$$

PMI	3.2

E-Factor				
**Waste**	**Mass (g)**		**Output**	**Mass (g)**
Ethylene Glycol	31.8		MeLA	7.1
Catalyst	1.2		BPA	4.0
Methanol lost from dist. 1	2.1		BHET	6.2
Methanol lost from dist. 2	7.4		Total	17.3
Water	40			
Total	82.5			

$$E-Factor = \frac{mass\ of\ waste}{mass\ of\ product}$$

E-Factor	4.8

Figure S30 Green metrics calculations for PMI and E-Factor.

7. Crystallography data

Table S9 X-ray crystallographic parameters.

8. Comparison with zinc (II) imino-pyrrole catalysts

Compound reference	Zn(**1**)$_2$	Zn(**2**)$_2$
Chemical formula	$C_{26}H_{32}N_6Zn$	$C_{28}H_{36}N_6Zn$
Formula Mass	493.94	522.00
Crystal system	Orthorhombic	Monoclinic
a/Å	14.2943(2)	12.27060(10)
b/Å	15.07500(10)	10.4330(10)
c/Å	22.6105(3)	20.83390(10)
α/°	90	90
β/°	90	90.950(10)
γ/°	90	90
Unit cell volume/Å^3	4872.26(10)	2666.80(3)
Temperature/K	150(2)	150(2)
Space group	*Pbca*	$P2_1/c$
No. of formula units per unit cell, Z	8	4
No. of reflections measured	142497	52718
No. of independent reflections	5180	5324
R_{int}	0.1140	0.0339
Final R_1 values ($I > 2\sigma(I)$)	0.0376	0.0257
Final $wR(F^2)$ values ($I > 2\sigma(I)$)	0.1156	0.0729
Final R_1 values (all data)	0.0419	0.0261
Final $wR(F^2)$ values (all data)	0.1196	0.0733
CCDC deposition number	2556631	2556632

	**Zn Pyrrole**	**Zn Indole**
**Activity**	High	Moderate
**Catalyst loading**	4 – 8 wt%	2 – 8 wt%
**Temperature**	80 –130 °C	70 –130 °C
**PLA/BPA-PC selectivity**	94%	>99%
**Mixed Plastic**	2-component	3-component
**Scale**	Up to 10 g	Up to 15 g
**Solvent recovery**	Yes	Yes
**Catalyst reuse**	15 cycles tested	10 cycles tested
**Reference**	*Angew. Chem. Int. Ed.* 2025 e202502845	This work

Table S10 Comparison of Zn (II) indole and Zn(II) pyrrole catalysts

9. Catalyst reuse

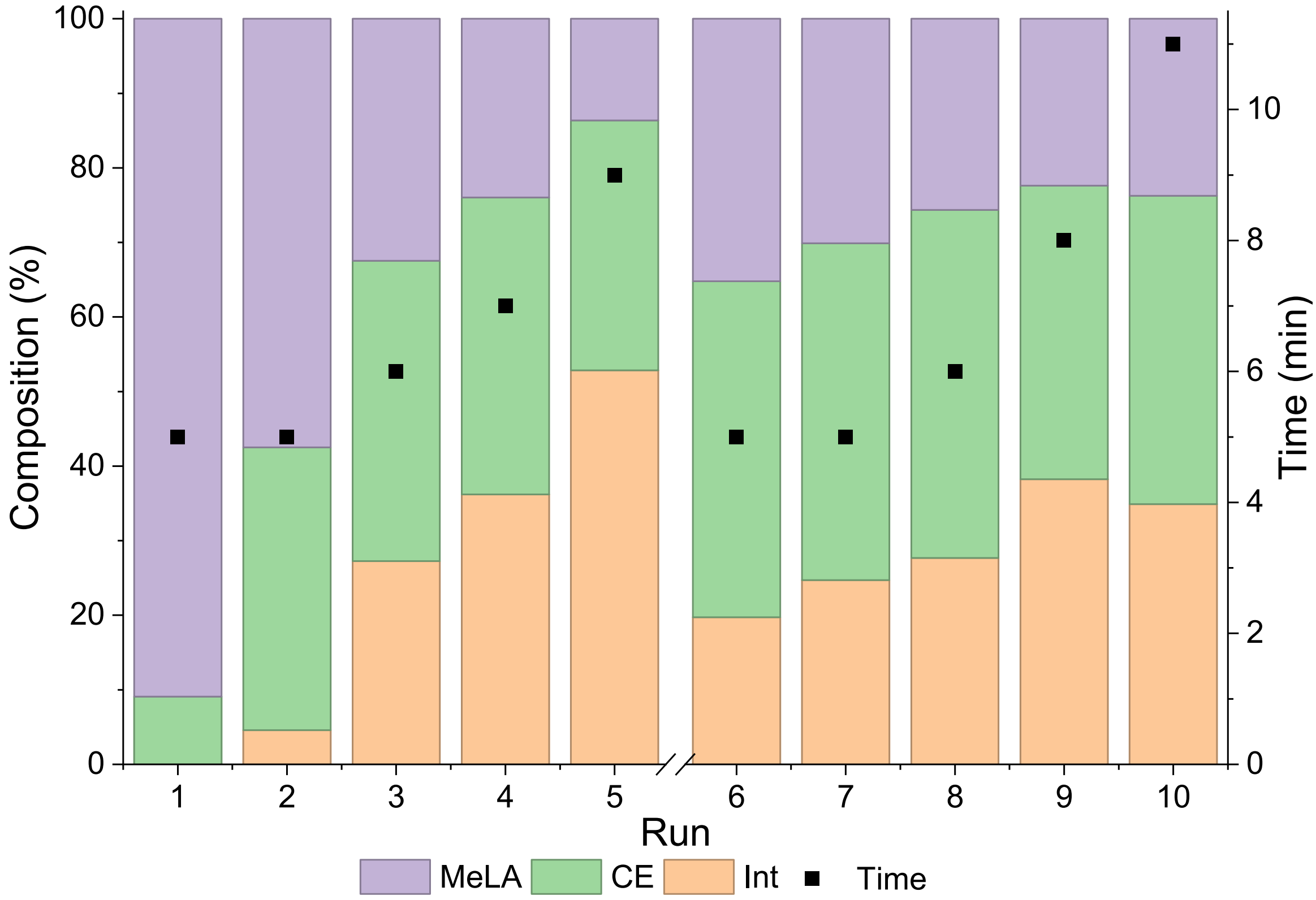

Figure S31 Catalyst recycling test. Reaction conditions: 0.25 g of PLLA cup (M_n = 45,510 g mol^{-1}), MeOH (2 mL, n_{MeOH}/n_{ester} = 14 : 1), 4 wt% cat. loading (10 mg, 0.78 mol% relative to ester linkages). ^{1}H NMR (400 MHz, 298 K, $CDCl_3$) spectroscopy used to calculate Y_{MeLA}, S_{MeLA} and X_{Int}. After each run, volatiles were removed and a fresh portion of PLLA cup (0.25 g) and methanol (2 mL) were added. Zn(**3**)$_2$ (10 mg) was added after run 5.
